# High contrast fluorescence polarization microscopy through double tagged photoswitchable fluorescent proteins

**DOI:** 10.1038/s44303-025-00094-y

**Published:** 2025-07-02

**Authors:** Lukas J. Münker, Manuel Hohgardt, Andreas Albrecht, Dominik Pfennig, Jan S. Tegtmeier, Andreas Holz, Marta Zagrebelsky, Martin Korte, Peter J. Walla

**Affiliations:** 1 Technical University of Braunschweig, Braunschweig, Germany; 2 Helmholtz Centre for Infection Research, Braunschweig, Germany

**Keywords:** Microscopy, Polarization microscopy, Wide-field fluorescence microscopy, Fluorescence imaging, Cellular imaging

## Abstract

We demonstrate that rigid anchoring of fluorescent proteins through double tagging (FPs) in living cells can significantly enhance the contrast in fluorescence polarization microscopy (FPM) by locking the transition dipole moment orientations to the sample’s structures. We applied double tagging of reversibly photoswitchable FPs (dt-rsFPs) to membranes and present a novel camera frame-separated switching pulse scheme that allows effective narrowing of the angle range of excited dt-FP also in living cells (frame-separated excitation polarization angle narrowing, FrExPAN). The principle of rigid anchoring allows specific selection of signals from different structural cell parts with slightly different orientations and is broadly applicable. FrExPAN imaging with dt-rsFPs double-tagged to membranes of living HeLa cells and living hippocampal neurons is demonstrated. We discuss potential implications for orientational contrast imaging as well as super-resolution by polarization demodulation (SPoD) methods.

## Introduction

In recent years, fluorescence microscopy with polarization contrast has increasingly become the subject for advanced imaging methods and other purposes^1–14^. Very often, additional details about the underlying structure can be gained through the extra information provided by the polarization of fluorescence molecules attached to membranes, cytoskeleton or other parts of the cell. FPM provides orientational information, orientation contrast and insights into orientational changes on different time scales in the underlying structures or allows investigating changes in phospholipid membrane curvature^15–17^. It has also been attempted to gain additional sub-diffraction-limited structural information about the samples, taking advantage of the different orientations of the various structural components on the nanoscale^2,18,19^.

**Fig. 1:**
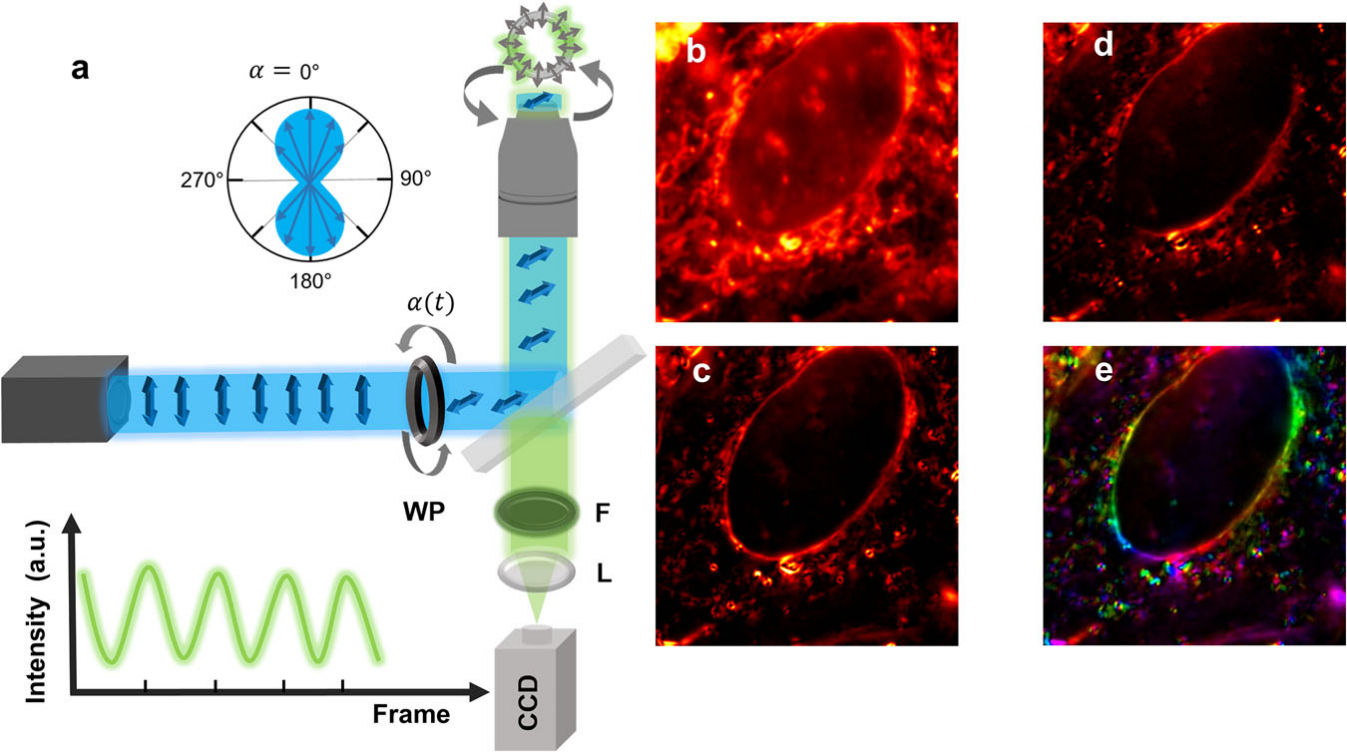
Example of additional information provided by fluorescence polarization microscopy (FPM). **a** Setup for fluorescence polarization microscopy by rotation of the excitation polarization^35^. **b** Conventional image of A549 lung cancer cells labeled by LAURDAN. **c** Image of only the modulation amplitude, A (c.f Eq. (1)), observed in FPM. **d** Image of the amplitude, A, of only one selected frame excited with a distinct polarization angle, α. **e** Modulation amplitude image of c, color-coded by the observed modulation phases (x, cf. Equation (2)) of different membrane parts.

In general, methods based on fluorescence polarization microscopy (FPM) take advantage of the fact that fluorophores are preferably excited by polarized light with polarization vector orientations (blue arrows in Fig. 1a) parallel to the orientation of their transition dipole moment (TDM, gray arrows in Fig. 1a), or that the emission of the fluorophores is preferentially polarized parallel to their TDM. In Fig. 1b–e, the potential of this additional information is illustrated using A549 lung cancer cells labeled by LAURDAN. When rotating the excitation-polarization angle, α, different parts of the cell show up at different times. This provides additional structural and orientation information of the labeled structures and allows dissection of different parts of the sample that cannot be distinguished when observed without the polarization information^20^. If the structures move, important orientational changes during mechanistic processes can also be detected. An extension is possible when using FPM techniques that allow detection of the full 3D-orientation, thereby increasing the distinguishable orientation/polarization space significantly^11,21–27^.

However, the gain of information from FPM techniques like LC-PolSCope^28^, two-photon polarization microscopy^4^, excitation polarization-resolved confocal fluorescence microscopy^5^, total internal reflection fluorescence (TIRF), time-resolved single-molecule fluorescence polarization^6^ or super-resolution by polarization demodulation/excitation polarization angle narrowing (SPoD/SPoD-ExPAN)^18^ and methods such as super-resolution dipole orientation mapping (SDOM)^29^, polarized dSTORM (P-dSTORM)^30^ as well as polarized structured illumination microscopy (pSIM)^19^ and single molecule orientation and localization microscopy (SMOLM)^31^ is often limited by the flexibility of the fluorophores linker during the acquisition time^18,19,29^.

Thus, a general limitation in all these approaches is the presence of potential orientational fluctuations of the fluorescent tags attached to the structures. There are examples of very rigid relations between the label’s orientation—and thus their corresponding transition dipole moments—and the structures^32,33^. For example, it has been shown that the transition dipole moments of phalloidin-Atto 590 in actin filaments are always oriented perpendicular to the fiber direction, thereby providing very strong orientation and polarization contrast even in raw data of such samples^34–39^. Another example is the labeling of membranes by LAURDAN, such as shown in Fig. 1^15,40–42^. The color-coded image reflects the different orientations of the fluorophores attached to different parts of the membrane.

Unfortunately, however, often FPs or any other fluorescence labels unilaterally attached (st-FPs) to samples, such as membranes, are capable of changing their orientation due to movement and diffusion along the membrane^5,43,44^. Since FPM methods achieve contrast by analyzing the orientation of the transition dipole moment, the occurrence of orientation changes during the measurement leads to deteriorated polarization contrast or localization precision. In these cases, the fluorescence labels have no direct structure-orientation relationship, and orientation signals are very difficult to interpret. Therefore, the application range of such methods has so far been limited to special cases, in particular, for live-cell imaging.

**Fig. 2:**
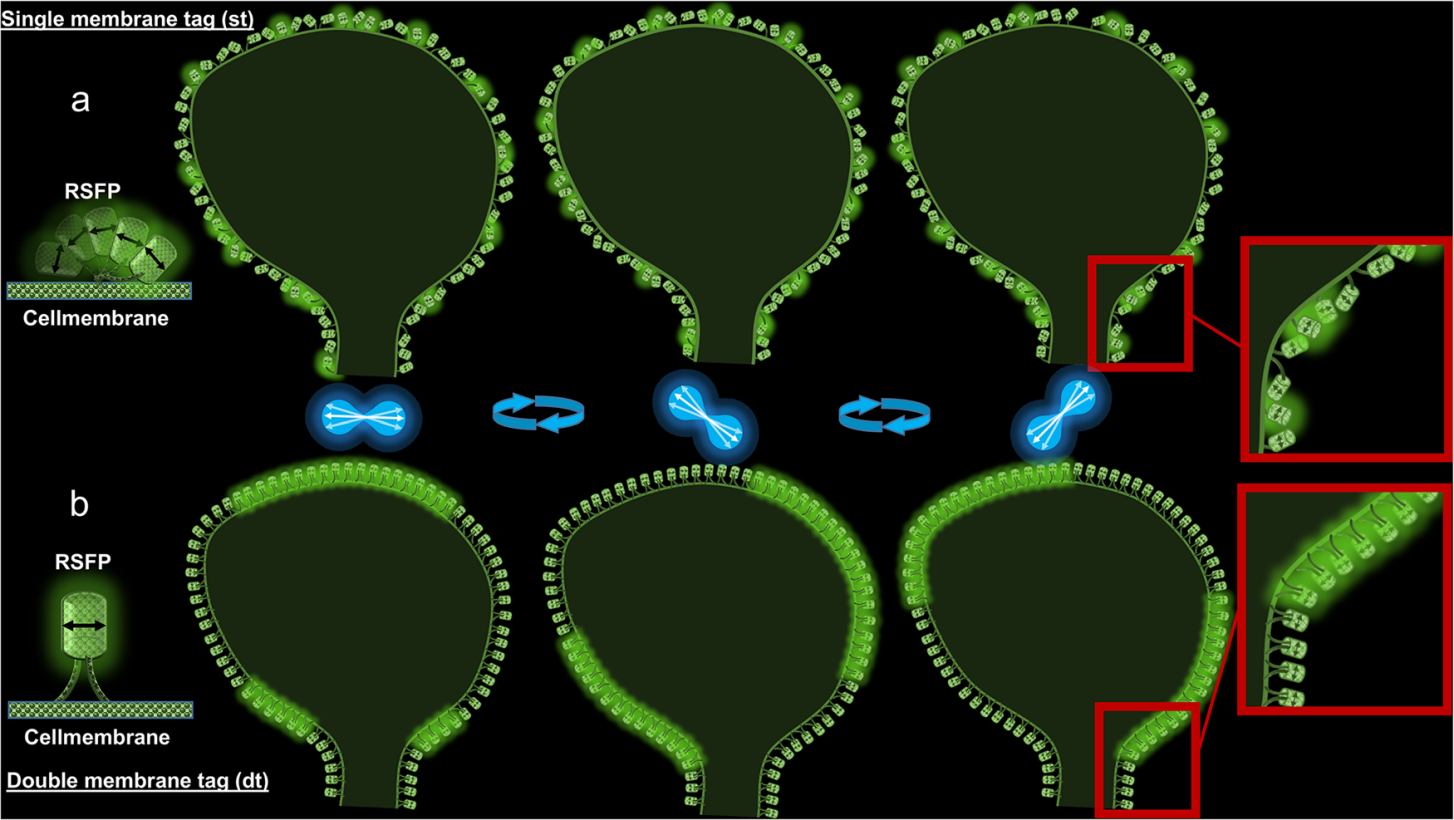
Principle of fluorescence polarisation microscopy (FPM) with fluorescent proteins double tagged to membranes. Illustration of the difference between single- (**a**) and double- (**b**) membrane-tagged rsFPs during constant polarization modulation of the excitation beam in an FPM setup such as shown in Fig. 1a. The rsFPs are preferentially excited by the light if the orientation of their transition dipole moment (shown as black arrows inside the barrel structure) is nearly parallel to the polarization vector (white arrow inside blue probability distribution of excitation). While st-FPs with one membrane tag are still flexible in the rotation around their anchor (**a**), the double-membrane tag restricts the free movement of the dt-FPs and leads to an orientation dependence on the shape of the tagged structure (**b**). This results in a much higher, better-defined modulation contrast of adjacent proteins in fluorescence polarization microscopy (FPM).

To circumvent such difficulties in orientation/polarization based microscopy methods, we present here an approach to anchor fluorescence markers rigidly by double tagging and demonstrate this approach with fluorescent proteins tagged to membranes of living cells (dt-FPs, Fig. 2). Such rigid anchoring of fluorescence proteins is not limited to structures such as membranes and can also be applied to the cytoskeleton or other cell parts to improve contrast in FPM methods. Here, double tagging was obtained by using both farnesylation and palmitoylation of fluorescent proteins. We demonstrate a significantly improved orientation contrast in living HeLa cells as well as in living neurons, but double tagging will be useful for any polarization/label orientation-based fluorescence method mentioned above.

In addition, we present different double-tagged photo-switchable fluorescent proteins (dt-rsFPs) and developed a novel laser pulse scheme that enabled excitation polarization angle narrowing much more effectively than previously possible in living cells (FrExPAN). We observed an angle narrowing of excited dt-rsFPs with high ExPAN factors in living HeLa cells and living hippocampal neurons, thereby significantly increasing the polarization contrast in FPM methods.

## Results

To improve the rigidity of fluorophore alignment at the cell membrane, we added both a farnesylation and palmitoylation membrane-targeting sequence, respectively, at the C- and N-terminus of the RSFPs Kohinoor^45^ and rsGreenF^46^ by cloning. This resulted in the expression of dt-FPs (p-Kohinoor-F, p-rsGreenF-F), providing a significantly higher transition dipole alignment when compared to st-FPs (p-rsGreenF, Kohinoor-F) with only one membrane-anchoring sequence (Fig. 2).

### Contrast enhancement in fluorescence polarization microscopy (FPM) by dt-FPs

To demonstrate the differences between single- and double-membrane tags, we first expressed farnesylated Kohinoor (Kohinoor-F) and farnesylated and palmitoylated Kohinoor (p-Kohinoor-F), palmitoylated rsGreenF (p-rsGreenF) and farnesylated and palmitoylated rsGreenF (p-rsGreenF-F) in HeLa cells and primary hippocampal neurons. The cell samples were first investigated by polarization modulation (Fig. 1a), followed by analysis of the resulting modulation amplitude, A, and the phase, x, of the fluorescence observed in each pixel.

**Fig. 3:**
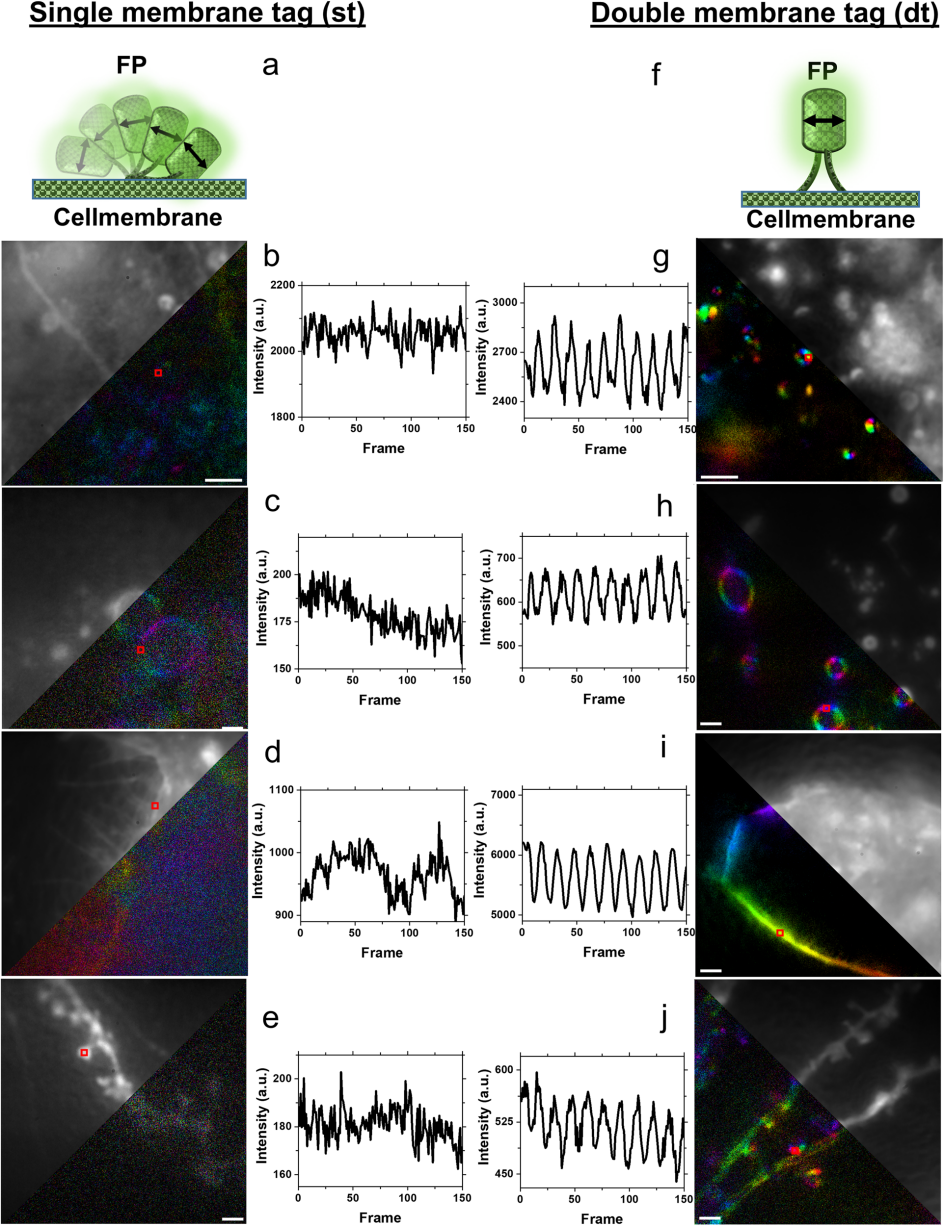
Fluorescence polarisation microscopy (FPM) data of single tagged (st) and double tagged (dt) fluorescent proteins. Principle of single (**a**) and double (**f**) membrane-tagged RSFPs. FPM results from **b** living HeLa cell samples expressing the single-tagged Kohinoor-F, **c** HeLa cell samples expressing p-rsGreenF, **d** HeLa cells expressing the single-tagged rsGreenF-F, **e** Dendritic branch of a hippocampal neuron expressing the single-tagged rsGreenF-F, **g** HeLa cells expressing the double-tagged p-Kohinoor-F, **h** HeLa cell expressing the double-tagged p-rsGreenF-F, **i** HeLa cell expressing the double-tagged p-rsGreenF-F, **j** Dendritic branch of a hippocampal neuron expressing the double-tagged p-rsGreenF-F. Shown is the average and phase colored FFT image of the different structures with the corresponding background-corrected raw data along with fluorescence polarization modulation data from selected ROIs(indicated in the images by red squares). Excitation wavelengths were 488 nm for Kohinoor and 405 and 488 nm for rsGreenF samples. Scale bars: 2 µm, a.u. arbitrary units. ROIs are highlighted with a red box and have a size of 495×495 nm for rsGreenF and 286 × 286 nm for Kohinoor results. The corresponding modulation signals are shown in Supplementary Movie 1. Very similar data have been observed with more biological replicates, and further examples can be found in Supplementary Movie 1b-d.

The different phases are a direct readout for the different orientations of the FPs (see also supplementary Movie 1). The determination of the phase was done by fast Fourier transformation (FFT) of the average of several periods of the modulation signals observed in each pixel, followed by visualization of the results through color-coding of the modulation amplitudes depending on their phase (Fig. 3 and Supplementary Movie 1a-d).

As can be seen by the false color images encoding the detected polarization/orientation along the membranes and the modulation signals, the structure-dependent fluorescence polarization contrast and amplitudes are significantly higher when using dt-FPs (Fig. 3f–j) compared to the single-tagged ones (Fig. 3a–i) under otherwise identical conditions. dt-FPs located next to each other exhibit a similar fluorescence phase compared to a more random fluorescence phase distribution in samples expressing st-FPs. Frame-dependent intensity plots derived from small regions of interest (ROIs; 11 × 11pixels, indicated by red squares in Fig. 3b–j) show strong differences in the modulation amplitude and thus polarization in both the cell membrane and in cytoplasmic vesicles of HeLa cells expressing single- versus double-tagged FPs (compare Fig. 3b, c with Fig. 3g, h). Also, in dendritic spine heads of primary hippocampal neurons, the expression of dt-FPs results in an improved polarization contrast and modulation (compare Fig. 3e with Fig. 3j).

Membrane double tagging for polarization and ExPAN contrast enhancement is applicable to all membrane parts of all cellular organelles. But the principle of double tagging for polarization and ExPAN contrast enhancement is not restricted to membranes. Other structural elements—such as the cytoskeleton—could be targeted in future studies to improve polarization and ExPAN contrast using different tags such as SNAP or Halo tags, for example.

### Double tagging of reversibly switching rsFPs and a novel laser pulse scheme for highly improved polarization contrast through frame-separated excitation polarization angle narrowing (FrExPAN)

In 2014, we demonstrated^18^ that the modulation contrast in FPM methods can be enhanced by significantly narrowing the angle range of fluorophore orientations excited by linearly polarized light (excitation polarization angle narrowing, ExPAN).

In conventional FPM, the modulation signals such as shown in Fig. 3, ideally follow a function

1 $${I}_{{Fl}}\left(\alpha \right)={y}_{0}+A{\cos }^{2}\left(\alpha -x\right)$$

with ${I}_{{Fl}}\left(\alpha \right)$, being the excitation polarization angle-dependent fluorescence intensity, α being the polarization rotation angle (see Fig. 1a), ${y}_{0}$ being a potential signal offset, $A$ the observed modulation amplitude, and x an orientation-dependent phase shift of the modulation signals. These signals are broad and unspecific, making the distinction of different structure/label orientations difficult, in particular when many different orientations are present. Several ${\cos }^{2}\alpha +x$ signals stemming from different orientations x result in summary in another ${\cos }^{2}\alpha +x$ function with an averaged orientation x that does not allow to disentangle any of the originally contributing orientations.

**Fig. 4:**
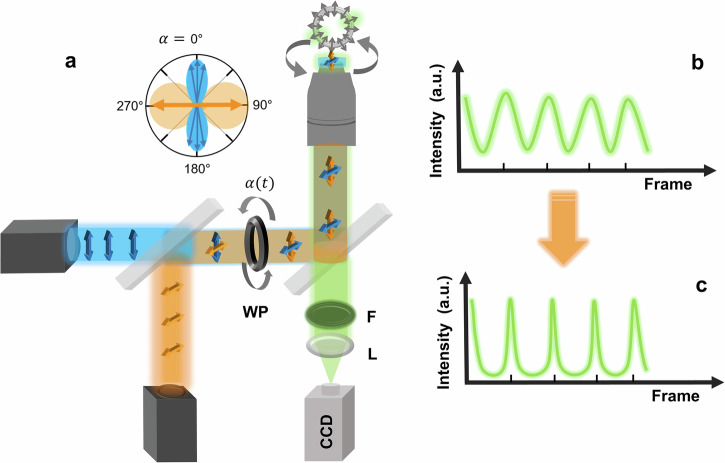
Setup and principle of ExPAN. **a** Setup. Like in conventional FPM (Fig. 1), the excitation polarization in a wide-field fluorescence microscope (blue beam with blue arrows) is rotated at a fixed frequency using, for example, a λ/2-wave plate (WP). In ExPAN, however, an additional wide-field deexcitation beam (orange beam) is rotated with a polarization always perpendicular to the excitation beam (orange arrows), resulting in much narrower modulation signals. **b**, **c** Modulation signals from a single molecule without (**b**) and with (**c**) ExPAN beam^18^.

Therefore, we previously introduced ExPAN by combining a traditional FPM with a second laser beam for deexcitation of all undesired orientations (For details see ref. ^18^). Briefly, the second beam is rotating with the same speed as the excitation beam but always has a polarization perpendicular (orange arrows in Fig. 4a) to the original excitation beam (blue arrows in Fig. 4a). The second, perpendicular deexciting beam suppresses emission from all molecules, that are not exactly parallel to the polarization of the initial excitation beam. Thereby, we were able to demonstrate a significant narrowing of the angle range of excited fluorophores in the sample (compare Fig. 4b that shows how a signal from a single fixed fluorophore observed in conventional FPM looks like (Eq. (1)), with a signal from the same molecule when using ExPAN, Fig. 4c and Eq. (2)).

The off-switch-beam results in a change of the periodic signal of the fluorophores from a cosine-squared function (Eq. (1)) to a narrowed function described by (Eq. (2), Fig. 4c)^47,48^:

2 $${I}_{{Fl}}\left(\alpha \right)={y}_{0}+A\cdot \frac{{\cos }^{2}\left(\alpha -x\right)}{1+{f}_{\mathrm{ExPAN}}{\sin }^{2}\left(\alpha -x\right)}$$

Here, α, is the polarization rotation angle, ${y}_{0}$ is the signal offset, $A$ the observed modulation amplitude, x the phase shift and *f*_ExPAN_ corresponds to the ExPAN factor, which quantifies the extent of narrowing of the cosine-squared function.

However, although ExPAN is in principle able to greatly improve the differentiation of transition dipole moment angles of fluorophores with different orientations and contrast in FPM methods, it is still very susceptible to label orientation fluctuations during the measurement and quickly weakened by any wobbling or rotation of the fluorescent label^2,18,19^.

Thus, in living samples that often contain fluorescent labels that are not rigidly fixed, very little contrast can be observed in FPM even when additionally using ExPAN, simply because the fluorophores undergo orientation fluctuations that are too fast for the detecting cameras. For example, a free FP has a rotational correlation time of about 20 ns in water. If it is attached to a membrane there are still orientational fluctuations faster than the camera frame rate that depend—among other factors—on the linker length but the angle range of possible orientational fluctuations is already more restricted leading to some average orientation persisting on time scales longer than the camera frame rate. However, with double tagging, the angle range of the free orientation fluctuations is drastically reduced, thus resulting in large polarization contrast enhancement.

Therefore, we also tested whether the use of dt-FPs can further improve the orientation and polarization contrast when using ExPAN. The effect of excitation angle narrowing was first demonstrated using stimulated emission depletion^18,49^. However, ExPAN is not restricted to the type of deexcitation mechanism. Recently developed reversibly switchable fluorescent proteins (rsFPs) with improved brightness, fluorescence yield and switching speed, like Kohinoor and Kohinoor 2.0^45^, rsGreenF^46^, or Padron2^50^ can potentially also be used for the switching mechanism in ExPAN with significantly less intense off-switch beams^13,14^.

In fact, the data shown in Fig. 3 already show signals obtained with both, positively switching dt-rsFPs such as Kohinoor (Fig. 3b, g) as well as negatively switching dt-rsFPs such as rsGreenF (Fig. 3h–j).

Positively switching rsFPs have the advantage of not requiring additional illumination steps, but many more negatively switching rsFPs were described to this day and recent advances even resulted in the development of a switchable Ca^2+^ indicator rsGCaMP showing negative-switching behavior similar RSFPs like rsEGFP^51^.

In general, ideal photophysical properties of membrane-double-tagged rsFPs for ExPAN applications are not only fluorescence brightness, on/off contrast ratio, and photostability, but it is also particularly important that their switching is not too slow. Different parts of membranes of slightly different orientation can be better differentiated, the narrower angle range can be achieved by ExPAN. When using rsFPs double-tagged to membranes, smaller fluorescence brightnesses can be compensated by larger numbers of rsFPs of the same orientation in individual membrane parts, leading to strong signals, and lower photostability can be partially compensated by diffusion of fresh rsFPs into individual membrane parts. So, poor fluorescence brightness and photostabilities are slightly less severe in our method. The on/off contrast ratio is not unimportant, as it heavily influences the possible angle-narrowing contrast. However, in the very moment of observing individual membrane parts by high angle resolution, it is not only very important that the dt-rsFPs do not change their orientation but also that they do not diffuse into other membrane parts with different membrane angles before they can be observed after switching off rsFPs with the “wrong” orientation. Thus, fast switching is at least as important as the other photophysical properties, and therefore, the specific rsFPs for our study were selected because they have fast switching times.

In a first test, we expressed ns-RSFPs (negative-switching reversible switchable fluorescent Proteins) for live-cell-imaging with ExPAN and took advantage of the improved modulation dependency of the double-membrane-tagged RSFP p-rsGreenF-F in primary hippocampal neurons and HeLa cells.

**Fig. 5:**
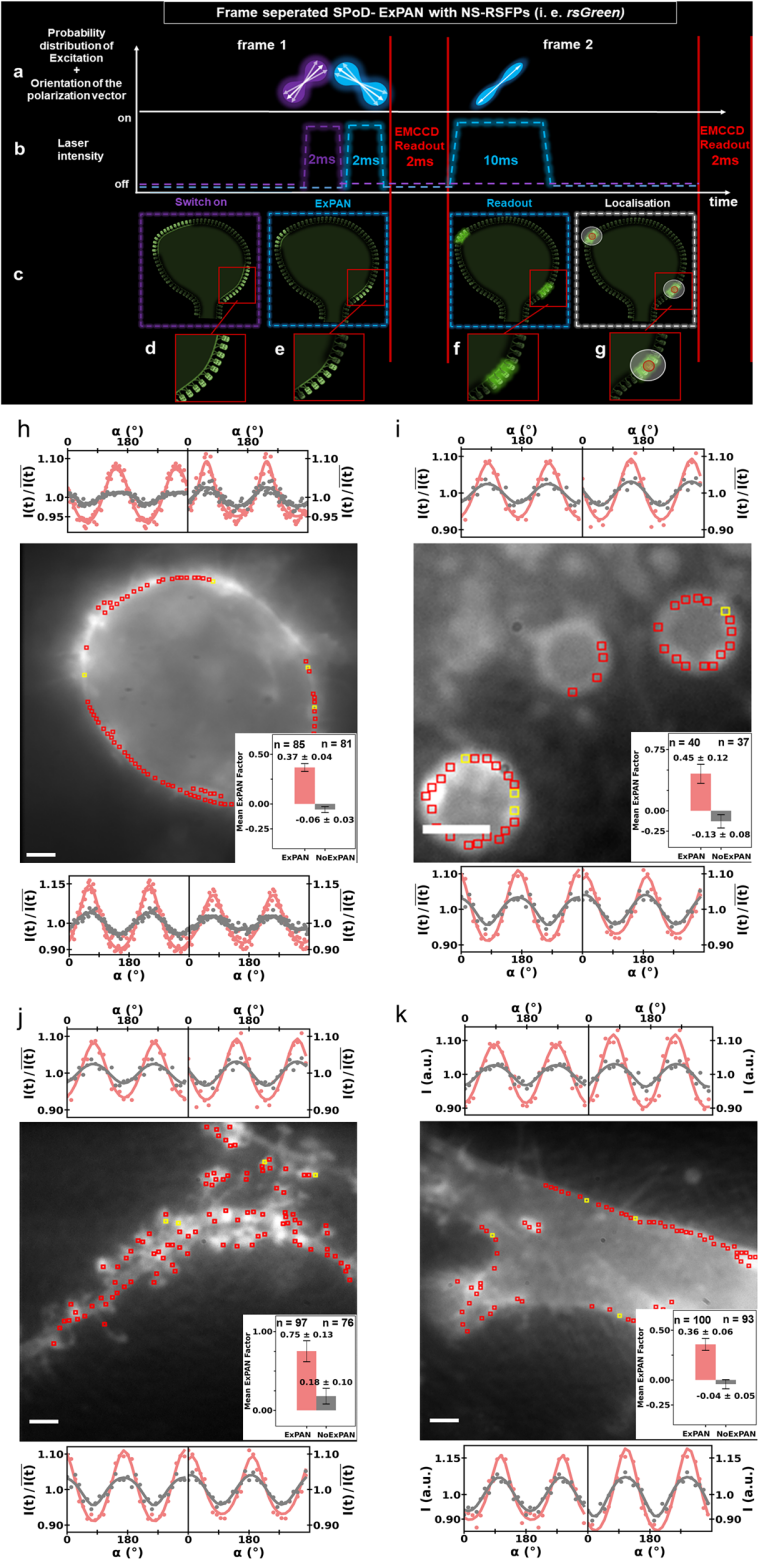
Principle of frame-separated excitation polarization angle narrowing (FrExPAN) for the usage with negative-switching dt-rsFPs. Shown is **a** the polarization orientation of the laser pulses and **b** the pulse duration, together with **c** a schematic spinehead-like structure with double membrane-tagged dt-rsFPs. At the end of the first frame, the dt-rsFPs get switched on by a short 2-ms pulse of 405 nm (**d**) directly followed by 2 ms of 488 nm with a polarization vector perpendicular to the 405 nm laser to achieve the excitation angle narrowing (**e**). The following readout step is moved into the next frame to enable fluorescence detection of dt-rsFPs with a transition dipole moment exactly parallel to the polarization vector of the switch-on and readout laser without detecting the intense fluorescence from the perpendicular polarized switch-off beam in the previous frame (**f**). Due to the camera readout time, there is a delay of 2 ms before the next pulse to read out the activated FPs. The readout pulse is 10 ms long and is positioned directly at the beginning of the second frame, with consideration of the 1 ms readout time of the camera between two frames to keep the time available for the diffusion of the proteins along the membrane as low as possible. In the future, the data can be analyzed by advanced algorithms, for example, localization of regions with multiple dt-rsFPs of narrowed and similar orientation angles (**g**). For details, see the text. **h**–**k** Experimental frame-separated FrExPAN from double membrane-anchored dt-p-rsGreenF-F HeLa cell and hippocampal neuron samples. The images show the average intensity of modulation averages observed with 50 frames (**h**) or 15 frames (**i**–**k**) per period. (The corresponding modulation signals are shown in Supplementary Movie 2). In addition, modulation signals of four selected ROIs are shown for each image as observed with (red, ExPAN) and without a second FrExPAN pulse (black, noExPAN). The inset in the lower right of each image (**h**-k) shows averaged ExPAN factors along with error bars from data with (red, ExPAN) and without second FrExPAN pulse (black, noExPAN) observed in at least 99 ROIs for FrExPAN and for noExPAN, respectively. **h** Experiments were conducted with a 5 ms switch-on with 405 nm, a 1 ms FrExPAN and 50 ms readout pulse and 50 frames per period. **i**–**k** were done with a 2 ms switch-on with 405 nm, a 2 ms FrExPAN and a 10 ms readout pulse and 15 frames per period. **h** Data from HeLa cell membranes (**i**), vesicles of HeLa cells (**j**), primary hippocampal neurons (**k**), and another HeLa cell membrane example. Scale bars 2 µm. For the plotting of the modulations signals a phase shift was applied to match the position of the maxima and the modulation signals were plotted twice for visual clarification of the narrowing effect. (for details see text).

To obtain an ExPAN effect with negative-switching RSFPs, we developed a measurement sequence that consists of three consecutive steps and frame separation (FrExPAN, Fig. 5a–g) and that has been optimized to find the highest possible ExPAN factors, *f*_ExPAN_, in various living cells. In the first step, the fluorophores are switched on by a first linearly polarized, short pulse of 405-nm light, resulting in a distribution of excited angles as in conventional FPM (Fig. 5d). Immediately thereafter the samples are illuminated by a second short pulse from a high intensity 488 nm beam polarized perpendicular to the first switch-on Laser in the second step. The second switch-off beam is necessary to ensure that preferentially only fluorophores with a transition dipole moment oriented parallel to the polarization vector of the first switch-on will be readout in the next step (ExPAN effect, Fig. 5e). The short pulses and the short times between the pulses are necessary to keep potential orientation changes during all steps to a minimum. The first and second pulses were configured to be sequential in order to prevent the occurrence of multiple switching events during a single localization sequence. The third step in the measurement sequence is the readout of the RSFPs excitation angle narrowed using another, third pulse of 488 nm with a moderate intensity and a polarization parallel to the one of the original excitation (Fig. 5f). Again, short times between the second and third pulses are necessary to keep potential orientation changes during all steps to a minimum. In addition, to avoid the detection of strong fluorescence from the RSFPs during the high-intensity second 488 nm switch-off beam, it is necessary to separate this second high-intensity beam from the third 488 nm readout beam into different camera frames (Fig. 5a, b). In our setup, the EMCCD camera has a readout time of around 1 ms between two frames when using full frame transfer. Regarding the pulse durations during the optimization process, we used for the first 405 nm switch-on pulse short 2–6 ms activation pulses, while for the second switch-off and third readout 488 nm laser pulses, we used pulse durations of 1 and 2 ms, respectively, and increasing them up to 100 ms. In the last step, data processing of the observed signals is done (Fig.5g).

In Fig. 5h–k, we applied this frame-separated ExPAN detection scheme (Fig. 5a–g) to various live-cell samples labeled with double membrane-anchored dt-p-rsGreenF-F and compared the observed results for a multitude of ROIs with the results observed without the second ExPAN beam under otherwise identical conditions. In Fig. 5h, data from membranes of living HeLa cells observed with a polarization modulation resolution of 50 frames per period, corresponding to 50 different angle orientations of the polarization, are shown. In Fig. 5i, data from vesicles in living HeLa cell samples, in Fig. 5j, data from living primary hippocampal neurons, and in Fig. 5k, data from living HeLa cells all labeled by dt-p-rsGreenF-F are shown, all observed with a polarization modulation resolution of 15 frames per period. The corresponding modulation signals are shown in Supplementary Movie 2.

We developed an automated algorithm that identifies regions in an image with modulation contrast (see Material and Methods section) and fits Eq. (2) to all ROIs in order to determine the ExPAN Factor, *f*_ExPAN_, (Eq. (2).) for all ROIs. The ROIs selected by the algorithm for this statistical analysis are indicated by red squares in Fig. 5. We then optimized the timing and intensities of all three beams to maximize the globally fitted ExPAN factor in the living cells. For nearly all ROIs, an increase of the modulation amplitude, A (Eqs. (1, 2)) and a significant narrowing of the signals can be observed when using FrExPAN. This is exemplified by the individual modulation signals from four selected ROIs observed with (red) and without second FrExPAN pulse (black), which are shown for each sample (the entire fit results for all ROIs of Fig. 5h can be found as additional data in the supplementary Data 1) and is further confirmed by the average of the ExPAN factors of all ROIs indicated by red in the figures shown in the lower right of each image (Fig. 5h–k) for the data with (black) and without second FrExPAN pulse (black). Similar to the FPM results described above (3), we focused on vesicles within the labeled HeLa cell samples, the HeLa cell membrane and the membrane of dendritic spine heads. The experiments were done with living HeLa cells and primary hippocampal neurons expressing dt-p-rsGreenF-F, and for each measurement, 700 frames were recorded. The rotation speed of the polarization vector by the lambda half-wave-plate was set to 50 (Fig. 5h) or 15 (Fig. 5i–k) frames per period. When more frames were recorded per period, the narrowing effect of FrExPAN could be better observed, as the peak region is better resolved (compare the modulation signals in Fig. 5h with those of Fig. 5i–k).

In the future, algorithms could be developed to localize, for example, the position of structures with a narrow angle range, as in single-molecule localization microscopy and without complex deconvolution steps. Since double tagging results in a fixed relationship between membrane orientation and the orientation of the FPs (Fig. 2), many FPs can have similar orientations in nanoscopic regions of the sample with similar membrane orientation, resulting in a strong orientation signal from very small areas. The great advantage over methods localizing single molecules would be the much higher signal intensities stemming from many molecules in a small area of similar orientation and position (Fig. 5g). This would allow much higher localization speeds in living cells.

Overall the presented data obtained from imaging HeLa cells and hippocampal neurons show that independently of the size of the membrane structure combining frame-separated excitation polarization angle narrowing (FrExPAN) with expression of negative-switching, double-tagged fluorescent proteins (dt-rsFPs) results in a significant narrowing of the angles of excited molecules and thus in an increase in the modulation depth, signal-to-noise ratio and polarization contrast.

### Application of dt-rsFPs and FrExPAN enhancement for super-resolution by polarization demodulation (SPoD)

**Fig. 6:**
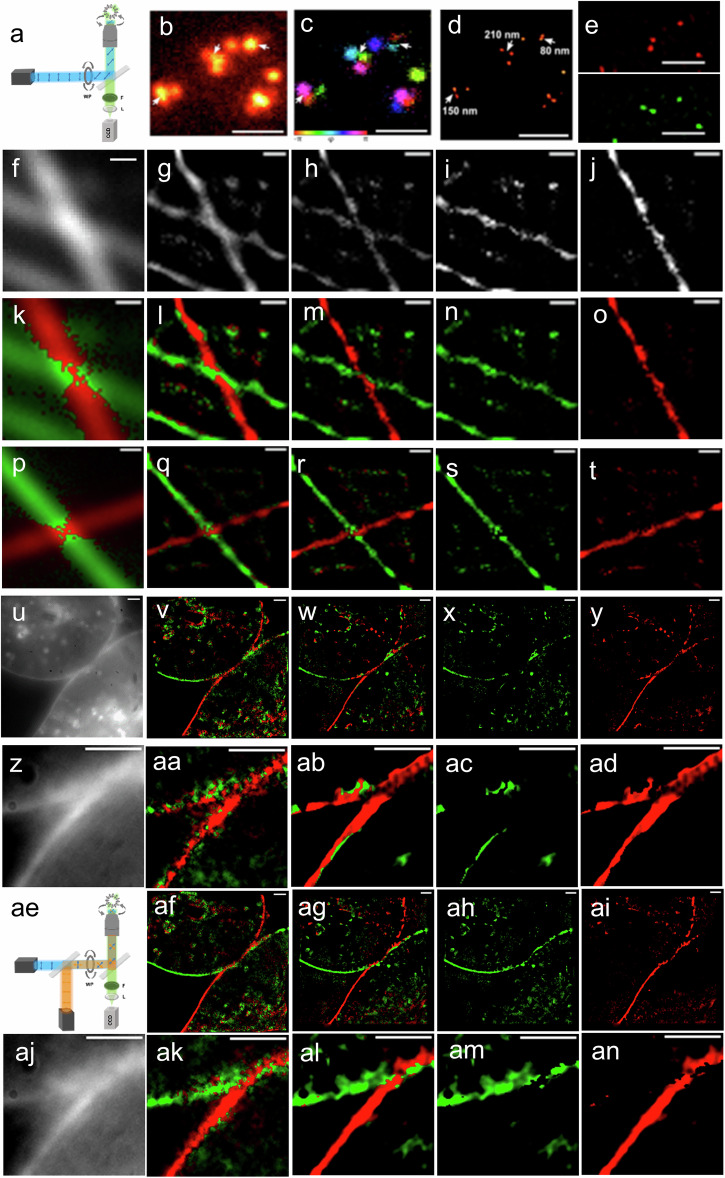
Subdiffraction image analysis of fluoresence polarisation microscopy (FPM) and frame-separated fluorescence excitation polarization angle narrowing polarization microscopy (FrExPAN) data. **a** Setup for fluorescence polarization microscopy by rotation of the excitation polarization (FPM). **b** Diffraction-limited image of single molecules and **c** false color version of the diffraction-limited image, color-coded based on the different orientations. **d** Results of the deconvolution with the SPEED algorithm to locate the molecules at sub-diffraction-limited resolution (single molecule Data previously published in Hafi, N. et al., Nature methods **13**, 8–9 (2016)^34^). **e** Two selected phase ranges from this analysis, separating molecules in different orientation ranges. **f–t** Shows different examples of linear actin filaments on a coverslip labeled with phalloidin-Atto 590 (averaged raw data previously published in Hafi, N. et al., Nature methods **13**, 8–9 (2016)^34^)^35^. **f** Averaged raw data with (**k**, **p**) orientation color-coded images of the diffraction-limited images. **g**, **l**, **q** Deconvolved images based on the entire signal and **h**, **m**, **r** deconvolved images based on only the modulating part (A in eq. 1,2) and **i**, **j**, **n**, **o**, **s**, **t** selected phase/orientation ranges using the ALPA algorithm (for details see methods section). **u**–**ad** FPM results of data from a double membrane-anchored dt-p-rsGreenF-F living HeLa cell without second FrExPAN pulse (NoExpan). **u**, **z** Averaged diffraction-limited raw data. **v**, **aa** FPM data deconvolved and orientation color-coded results after 500 iterations with Richardson–Lucy. **w**–**y**, **ab**–**ad** FPM data deconvolved and orientation color-coded images after 500 iterations with SPEED algorithm and with selected phase/orientation ranges (for details see text). **ae** Set-up for frame-separated fluorescence excitation polarization angle narrowing polarization microscopy (FrExPAN, for details see Fig. 5 and corresponding text). **af**–**an** Results of the FrExPAN data obtained under identical conditions as in (**u**–**ad**) but with second ExPAN pulse. **aj** Averaged diffraction-limited raw data. **af**, **ak** Deconvolved images after 500 iterations with a Richardson–Lucy algorithm. **ag**–**ai**, **al**–**an** Deconvolved images after 500 iterations with the SPEED algorithm. Scale bars **b**–**e** 1 µm, **f**–**t** 0.25 µm, and **u**–**an** 2 µm. (The corresponding modulation signals are shown in Supplementary Movies 3, 4) For details, see text.

Super-resolution by polarization demodulation (SPoD) is a method that uses different orientations of fluorescence markers to disentangle structures at distances below the diffraction limit. For example, the single molecule data shown in Fig. 6b–e and Supplementary Movie 3 demonstrate how close-by molecules of different orientation can be differentiated during fluorescence polarization modulation based on their different blinking phases x (Eqs. (1, 2)). Figure 6b shows a diffraction-limited image of these molecules, while Fig. 6c is a false color version of the diffraction-limited image where the different phases, x, and thus orientations, are color-coded. Figure 6d shows an image processed by an algorithm that can disentangle space and phase simultaneously (SPEED algorithm) to locate the molecules at sub-diffraction-limited resolution, and Fig. 6e shows two selected phase ranges from this analysis, separating molecules in different orientation ranges.

Figure 6f–t shows different examples of linear actin filaments on a coverslip labeled with phalloidin. In Figure 6f–t diffraction-limited images (Fig. 6f, k, p), phase color-coded images of the diffraction-limited images (Fig. 6k, p) and deconvolved images either based on the entire signal (y_0_+A in Eq. (1), Fig. 6g, l, q) or only the modulating part (A in Eq. (1), Fig. 6h, m, r) or selected phase/orientation ranges (Fig. 6i, j, n, o, s, t) are shown. The data clearly show, how the filaments can be separated in the crossing regions at sub-diffraction limited distances (see, for example, Fig. 6m) in a way that is not possible in the diffraction-limited images without polarization information (Fig. 6f). The actin filaments labeled with phalloidin (Fig. 6f–t) represent examples of a very close relationship between structure and label orientation as the phalloidin molecules always insert perpendicularly into the filament structure. Also, the example shown in Fig. 1 shows an example of a very close relationship between structure and label orientation. However, as mentioned already in the introduction, particularly in most living samples, the label orientation is rather arbitrary and fluctuating, resulting in FPM images such as in Fig. 3a–e that do not contain much useful information. This can fundamentally change using double-tagged fluorescence markers such as the rs-dt-FPs presented here since they enable in principle a close structure and label orientation relationship for any structural element of a biological sample.

In order to initially assess whether increased polarization contrast by FrExPAN using rs-dt-FPs in combination, can help to gain improved structural insights, we analyzed the dataset from two double-membrane-anchored dt-p-rsGreenF-F living HeLa cells once without the second FrExPAN pulse (“NoExPAN”, Fig. 6u–ad and Supplementary Movie 3u–ad) and analogously with FrExPAN (Fig. 6ae–an and supplementary Movie 3ae–an). Figure 6u, z, aj shows corresponding diffraction-limited images. Figure 6v, aa shows orientation color-coded versions of the modulation data deconvolved by a simple Richardson–Lucy algorithm (500 Iterations). Figure 6w, ab shows orientation color-coded versions of the modulation data deconvolved by the SPEED Algorithm (500 Iterations, λ_1_ = 0.20 and λ_2_ = 5.00). Figure 6x, y, ac, ad shows the same regions analyzed by the SPEED algorithm but only for a selected phase x and orientation range. In particular, Fig. 6ac, ad demonstrate that using FPM without additional ExPAN does not allow to properly separate the two membranes by their orientation neither in the sub-diffraction-limited contact region nor in other diffraction-limited regions and regardless of the selected label orientation range or algorithm used (see also Supplementary Movie 4a, b).

However, when applying FrExPAN under otherwise identical conditions (Fig. 6ae–an), does clearly allow to separate the two membrane parts even when they are very close (Fig. 6ak–an) and in regions in which they clearly cannot be separated by the diffraction-limited wide-field imaging (compare Fig. 6aj with Fig. 6ak–an and SPEED analysis in Supplementary Fig. 4b, compare panel g, h (FrExPAN) with panel o, p (noExPAN)). Also, a direct comparison of the modulation raw data obtained with FrExPAN (Supplementary Movie 3aj) demonstrates much better separation than without the second ExPAN pulse (Supplementary Movie 3z).

This provides evidence that the polarization information, only available through FrExPAN with dt-rsFPs, is essential to see these structural details in the living HeLa cells.

A detailed analysis of the capabilities at what sub-diffraction-limited distances we were able to distinguish membrane parts in living cells by their double-tagged label orientation (red, green) and FrExPAN under the distinct experimental conditions can be seen in Supplementary Fig. 1. It is difficult to tell what the highest possible resolution is with a certain set of experimental data as this depends on many factors in the experimental conditions an analysis. However, the analysis shown in Supplementary Fig. 1 clearly demonstrates that with double-tagged RSFPs, resolutions well below the diffraction limit are possible in wide-field images of living cells and that structures can be clearly resolved by FrExPAN (Supplementary Fig. 1n–q and Fig. 6ak–an) that are impossible to dissect in standard wide-field microscopy (Fig. 6aj and Supplementary Fig. 1m). We expect that in the future development of advanced algorithms will allow to extract such information in a more generic way than currently presented here.

## Discussion

Our results can be summarized as follows: (1) We demonstrated that the expression of double-tagged fluorescent proteins, increasing the rigidity of the anchoring of the FPs to structures of living cells, yields a much higher polarization contrast and signal-to-noise ratio in any FPM method due to significantly improved fluorescence polarization and modulation amplitude compared to conventional one-linker tagging^4–6,8,18,19,28,29,31^. (Figs. 2, 3 and Supplementary Movie 1a-d). (2) Double tagging of different fluorescent proteins (dt-FPs) by farnesylation and palmitoylation allows to lock their orientation to the membrane curvature in living cells. This enables dissecting image signals from different structures with high contrast and is broadly applicable (Figs. 3, 4, 6, Supplementary Movies 1, 2, 4). Likely, the double membrane anchor also results in a decrease in the diffusion speed along the membrane, which further helps to investigate highly dynamic, living structures with higher polarization precision. (3) Conventional excitation with linearly polarized light does always excite a broad, quite unspecific distribution of orientations around the excitation polarization vector (Fig. 2b, 5d). In contrast, rigidly attaching reversibly photo-switchable fluorescent proteins (dt-rsFPs) to the membrane by double tagging allows to detect specific signals from a narrow range of angles around the excitation polarization (Figs. 2c, 5e,f and Supplementary Movies 2–4) when applying a novel camera frame-separated switching scheme that switches off undesired orientations not parallel to the excitation polarization (Fig. 5a–f, frame-separated excitation polarization angle narrowing, FrExPAN, Figs. 5, 6). So far, very effective excitation polarization angle narrowing (ExPAN) was only shown for isolated molecules fixed on a surface^18^, but FrExPAN of dt-rsFPs also allows quite effective excitation angle narrowing in living cells (Fig. 5 and Supplementary Movie 2). Thus, this further development of ExPAN microscopy enables even higher polarization contrast and signal-to-noise ratio, also in living cell FPM methods. It also allows performing ExPAN at much lower intensities than previously, thereby enabling high ExPAN effects in very sensitive living cell types such as hippocampal neurons (Fig. 5). (4) The high angle-dependent selectivity of FrExPAN with dt-rsFPs also allows separating membrane parts in living cells that cannot be separated in any other conventional FPM method and even below diffraction-limited distances (super-resolution by polarization demodulation (SPoD) (Fig. 6ae–an, Supplementary Movies 2, 4, and Supplementary Fig. 1).

In future developments, the use of faster switching rsFP combined with further reduced pulse durations and improved rigidity of the orientation will allow for further narrowing of the angle range. Ultimately, this could result in very small areas of excited fluorophores in living samples, also at structures other than membranes. These small areas could be localized at nanometric resolutions similar to single molecule localization super-resolution methods (Fig. 5g) but with much stronger signals and thus, localization precision and frame rates since many more molecules contribute to the signals than in single molecule localization super-resolution methods.

If the goal of exclusive signals from many more than one chromophore of a very narrow angle range restricted to very small subdiffractional areas of membranes is achieved, such signals can be localized with the same algorithms as used for single molecule localization but with significantly higher precision and frame rate because they arise from multiple chromophores and not just one chromophore (Fig. 6g). This will then avoid the use of any sophisticated algorithm deconvolving the combined transition dipole angle and location space used for SPoD so far and replace it by a direct, straightforward localization algorithm. The new approaches of double anchoring switchable FPs to a certain cellular structure, here the membrane in combination with the new FrExPAN sequence, are an important step forward to this goal. However, certainly further steps are necessary, for example, extending the available orientation space for differentiation of different structures to 3D-orientation instead of just 2D orientation, tagging the FPs even more rigidly to the structures or developing faster switching rsFPs.

In any case, the orientation-rigid, double-anchored switchable dt-rsFPs presented here in combination with the low intensity FrExPAN pulse and observation sequences (Figs. 4–6 and Supplementary Movies 2, 4) enable a significant improvement in polarization contrast and excitation angle narrowing for any FPM method^4–6,8,18,19,28,29,31^ and this was exemplified with membranes of living cells. We demonstrated that low intensity FrExPAN with orientation-rigid, double-tagged switchable FPs is possible with membranes of living HeLa cells and even highly sensitive hippocampal neurons with significantly increased ExPAN factors (Figs. 5, 6 and Supplementary Movies 2). In the future, we expect further improvements with the development of more sophisticated orientational pulse and observation sequences and orientation-sensitive labels.

## Methods

### Preparation of single- and double-membrane-anchored fluorescent proteins

The single and double-tagged fluorescent proteins used in this study were produced by cloning a palmitoylation sequence at the N-terminus or a farnesylation sequence at the C-terminus or both to the DNA sequence coding for Kohinoor and rsGreenF. Farnesylation can only happen at the C-terminus of the protein and palmitoylation only at the N-terminus.

Kohinoor and rsGreenF were selected based on the following considerations. When using a photochemical photoswitching effect as an angle-narrowing deexcitation mechanism, the deexcitation tends to occur on longer time scales than when using the physical mechanism of stimulated emission. If the duration of angle narrowing by the deexcitation beam takes too long, FPs from other membrane areas and thus angles could diffuse into currently observed membrane parts, thereby decreasing the specificity, contrast and potential localization precision of the polarization. For example, it has been measured by FCS, FRAP and other methods that a single membrane-tagged GFP diffuses about 90 nm in 30 ms^52^. Kohinoor and rsGreenF have significantly faster switching half times than most other RSFPs. Kohinoor has a 54% increased half-time switching speed compared to the positively switching RSFP Padron^50^. rsGreenF, on the other hand, is an enhanced version of rsEGFP that shows a 31% faster off-switching halftime than rsEGFP^46^. Therefore, we originally decided to use these RSFP that have among the fastest switching times. In future studies, more rsFPs with potentially favorable properties for FrExPan can be tested. For example, rsEGFP2 has very similar properties to rsGreenF but has also quite good switching fatigue values^46^. In any case, it can be expected that the double tagging further reduces the membrane diffusion time and thus helps to further improve polarization contrast and the structural relation of orientation and membrane position.

The cloning resulted in the following constructs: Kohinoor farnesylated at the C-terminus, pc-DNA3-Kohinoor-F; Kohinoor palmitoylated at the N-terminus and farnesylated at the C-terminus, pc-DNA3-p-Kohinoor-F; rsGreenF palmitoylated at the N-terminus, pc-DNA3-p-rsGreenF; rsGreenF palmitoylated at the N-terminus and farnesylated at the C-terminus, pc-DNA3-p-rsGreenF-F. The constructs were inserted into a pc-DNA3 mammalian expression vector under a CMV promoter and used for transfection of HeLa cells and primary hippocampal neurons. The original expression plasmid for Kohinoor was provided by Takeharu Nagai and his research group, and rsGreenF was commercially purchased from Addgene (#78181)^45,46^. Upon receiving the plasmid, the cell is only able to express the given construct of the FP (in this case, the single-tagged or double-tagged). Since the cell is only given one plasmid at a time, the labeling efficiency is 100%, and there is no possibility that a double-tagged version does partially contain only one modification.

We cannot exclude that double tagging does artificially alter the shape of or physiological localization of different lipid components within the membrane. However, we saw no indication that the shape was different when using single- or double tagging. Also, when using unpolarized excitation light or averaging the signal from all different excitation polarization orientations, no major difference was visible when using single- or double-tagging.

While the current data do also not show with absolute certainty that 100 percent of the expressed FPs are double-tagged, any shortfall in the double-tagged fraction relative to the single-tagged fraction would only result in underestimating the true effect, i.e. such considerations do even further support the conclusion that increasing the ratio of double- to single-tagged fluorescent proteins enhances polarization contrast and the ability to narrow the excitation angle in fluorescence polarization microscopy.

### Cultivation and transfection of HeLa cells

The HeLa cells were obtained from the American Type Culture Collection (ATCC, CCL-2™). The cells were plated at a density of 9.3 × 10^4^ cells/cm^2^ on poly-L-lysine-coated coverslips (30 mm) cultivated in a DMEM+ (Dulbecco’s Modified Eagle’s Medium) supplemented with 4,5 g/L D-glucose and 10% FCS and in an incubator with 10% CO_2_ at 37 °C. After 1 day, the HeLa cells were transfected with the corresponding plasmids for the membrane-anchored Kohinoor-F,p-Kohinoor-F, rsGreenF-F, p-rsGreenF, and p-rsGreenF-F using polyethylenimine (PEI). Per well with 2 mL DMEM+ medium 2 µg of DNA were diluted in 200 µL DMEM- (without FCS) and 6 µL of PEI (1 mg/mL) were added. The mixture was vortexed and incubated at room temperature for 15 to 30 min followed by the dropwise addition of 200 µL of the DNA-PEI mixture to the HeLa cells.

The cells were then kept in the incubator for 4 to 6 h and afterwards washed at least six times with phosphate-buffered saline, followed by the addition of 2 mL DMEM+. After 12–36 h in the incubator, the HeLa cells were used for imaging in prewarmed phenol-red-free DMEM imaging medium. The imaging of the cells was performed at room temperature within the next few hours without any additional warming, medium exchange, or CO_2_ control.

### Preparation and transfection of primary hippocampal neurons

Primary hippocampal neuronal cultures were prepared from C57Bl/6 wild-type (WT) mice. The mice were bred and kept under standard housing conditions on a 12-h light-dark cycle with ad libitum access to water and food at the animal facility of the Technical University (TU) of Braunschweig, Germany. All experimental procedures were approved by the animal welfare representative of the TU Braunschweig and the LAVES (Oldenburg, Germany, Az. §4 (02.05) TSchB TU BS und Az.33.19-42,502-04-22/00099). The embryos of both sexes were removed from the amniotic sac at embryonic day E17.5 and rapidly decapitated. The heads were stored in ice-cold Gey’s balanced salt solution (GBSS) supplemented with 50% glucose and adjusted to pH 7.2. The hippocampi were dissected and put into 1 mL trypsin/EDTA solution and incubated for 25 min at 37 °C in a water bath. The Trypsin/EDTA solution was carefully removed, and serum medium (DMEM with 2% FCS) was added to stop the digestion.

After washing the hippocampi five times with serum medium, the tissue was carefully triturated through a Pasteur pipette and centrifuged in serum medium at 1500 rpm for 5 min the supernatant was removed and the cells were resuspended in 50 mL Neurobasal medium supplemented with 125 µL 200 mM L-glutamine, 1% N2 supplement and 2% B27 supplement (“NB+”). The cells were plated at a density of 5.6 × 10^4^ cells/cm^2^ on poly-L-lysine-coated coverslips (30 mm) in NB+. The plates were incubated for 2 weeks at 37 °C, 5% CO_2_ and 99% humidity.

The transfection of the neurons with the different rsGreenF and Kohinoor expression DNA plasmids was done using Lipofectamine2000 following the protocol proposed by ThermoFischer. For transfection, 8 µg of DNA and 10 µL of Lipofectamine2000 per well were both diluted in 500 µL Neurobasal medium without supplements (NB-) for 5 min at RT and mixed together afterward. The Neurobasal medium with supplements (NB+) of the cells was exchanged with NB- before the addition of the DNA/Lipofectamine2000-mixture. After incubation at RT for 20 min, the mixture was added dropwise to the cells. After 40–60 min in the incubator the medium of the cells was changed back to the conditioned NB+. The cells were left in the incubator for 24 to 48 h before imaging. For imaging, the cells were placed in a petri dish with prewarmed HBSS-medium (2.26 mM CaCl_2_, 5.33 mM KCl, 137.93 mM NaCl, 0.34 mM Na_2_HPO_4_, 13.1 mM D-Glucose, 0.44 mM KH_2_PO_4_, 0.49 mM MgCL_2_, 0.41 mM MgSO_4_, and 20.5 mM NaHCO_3)_. The imaging of the cells was done at room temperature in the next hours without any additional warming and medium exchange. All experiments were carried out in accordance with the applicable European and National regulations (Tierschutzgesetz) and were authorized by the LAVES (Oldenburg, Germany, Az. §4 (02.05) TSchB TU BS, 33.19-42502-04-22/00099) and the animal welfare representative of the TU Braunschweig.

### Preparation of linear actin filaments

The data for the linear actin filaments was taken from ref. ^34^, so the preparation procedure is identical as described therein: 250 µg of rabbit skeletal muscle G-actin (AKL99-A; Cytoskeleton, Inc., Denver, CO) were resuspended to 1 µg/µL using 250 µL of General Actin Buffer (BSA01; Cytoskeleton, Inc., Denver, CO) containing 0.2 mM ATP (New England Biolabs Inc., Ipswitch, MA) as well as 1.0 mM DTT (Carl Roth, Karlsruhe, Germany). After stirring, the mixture was cooled on ice for 1 h. About 5 µL of 10x polymerization buffer solution (BSA02; Cytoskeleton, Inc., Denver, CO) mixed with 45 µL of the previously produced solution were left for 1 h at room temperature to polymerize. Afterwards, the solution was diluted 100-fold using 1x polymerization buffer containing 70 nM Atto 590-phalloidin (ATTO-TEC, Siegen, Germany). A microscope coverslip was spotted with 10 µL of the solution containing the actin filaments. After the solution spread out, an additional 15 µL of ProLong Gold Antifade Mountant (Life Technologies, Darmstadt, Germany) were spotted in the center of the coverslip. Before measurement, the sample was left alone for more than 30 min.

### Experimental setup for single-molecule and linear actin filaments experiments

The two-dimensional polarization measurements were performed as previously described by Hafi et al.^18^. The Atto 590 labeled actin fibers are illuminated using a modelocked Ti:Sa laser (Chameleon Ultra II, 80 MHz, Coherent) and an optical parametric oscillator (OPO, APE) transforming the 800 nm laser output to 590 nm light. The beam is expanded using a telescope system. Afterward, the linearly polarized light passes a constantly rotating *λ*2-wave plate that rotates the polarization plane. The plate is rotated using a chopper system (Optical Chopper System, Thorlabs) that is synchronized to the EMCCD camera used for detection. A lens is used to focus the beam on the back aperture of the objective lens (NA = 1.35 oil immersion, UPlanSApo, 60x Olympus). The objective lens is mounted in an inverted microscope body (IX 71, Olympus). A dichroic mirror (dual-line beamsplitter zt 488/594 RDC, AHF Analysentechnik, Tuebingen, Germany) is used to separate the emission from the excitation. After passing through another telescope, the light is detected by the EMCCD camera (iXonEM+897 back-illuminated, Andor Technology).

### FPM setup and FrExPAN setup

Two configurations of a wide-field microscope setup were used to measure the samples. For the comparison of the single-tagged and double-tagged samples with FPM microscopy, configuration 1 was used. The excitation of the FPs was done with a linearly polarized 488-nm-continuous-wave (CW) laser (OXX-488-060, Oxxius) for the excitation of Kohinoor and, in the case of rsGreenF, combined with a linearly polarized 405 nm CW laser (OXX-405-060, Oxxius) for the on switching of rsGreenF. The polarization vector of the 488 nm CW laser was rotated with a λ-2-wave plate (λ-2 plate. 400–800 nm, Thorlabs) in order to rotate the polarization (Fig. 1a). The polarization vector of the 405 nm CW laser was rotated with an additional λ-2-wave plate (λ/2 plate. 405 nm, Thorlabs) in order to be parallel to the polarization vector of the 488 nm Laser. Both beams were passed through separate telescope systems. The two beams were then combined through a dichroic mirror and passed a rotating λ-2-wave plate (achromatic λ-2 plate, 340–560 nm, Edmund Optics) mounted on a motorized rotation stage (8MRU, Standa) which enables the continuous rotation of the polarization vector with one period of excitation polarization rotation corresponding to 15 or 30 frames. The motorized rotation stage is synchronized to the electron-multiplying charge-coupled device (EMCCD) camera (iXonEM+897 back-illuminated, Andor Technology). After passing through the λ-2-wave plates, the beams are focused onto the back aperture of the microscope objective (NA = 1.4 oil immersion, UPlanSApo, 100x, Olympus).

The microscope objective is mounted on a modified inverted microscope body (IX73, Olympus) in which the excitation light is transmitted through the beamsplitter (T495dcspxr, AHF) in the microscope body onto the sample to avoid changes of the polarization vector due to reflection. The emission light is reflected by the beamsplitter and directed through an additional lens system of achromatic doublets to magnify the image before reaching the detector of the EMCCD camera. Two emission filters (ET band pass 525/50, AHF and BrightLine HC 525/30, AHF) were positioned in front of the camera to clean up the fluorescence. All FPM experiments were conducted with an intensity of 2.44 W/cm^2^ for 488 nm for the Kohinoor tagged samples and 0.1 W/cm^2^ for 405 nm and 1.12 W/cm^2^ for 488 nm for the rsGreenF samples.

Configuration 2 was used for the FrExPAN experiments. It consists of all the components of Configuration 1 but is supplemented by an additional 488 nm CW laser (OXX-488-100, Oxxius) for the off-switch beam. The polarization vector of the off-switch beam is rotated with another λ-2-wave plate (λ-2 plate. 488 nm, Thorlabs) resulting in a polarization vector that is always perpendicular to the polarization vectors of the lasers from configuration 1 (Fig. 4a). The switch-off beam was passed through a telescope system and combined with the other 488 nm beam using a polarizing beamsplitter cube. In order to use the SPoD-ExPAN measurement scheme for negative-switching rsFPs described in this paper, the pulses of the lasers are generated with the help of function generators (4125, Peaktech), which are synchronized with the EMCCD camera. The settings for the λ-2-wave plate were set to take 30 (Fig. 5i–k), 60 (Fig. 6v–ad), and 100 (Fig. 5h) frames for one period of excitation polarization rotation to yield 15 ((Fig. 5i–k), 30 (Fig. 6v–ad), and 50 frames (Fig. 5h) for one period after cutting out the frames consisting only of the switch-on and ExPAN beam (Fig. 5a–c). For Fig. 6v–ad, only 240 frames of the background-corrected readout data were used to yield an even number of six averaged frames with an applied rotation speed of 30 frames.

For the FrExPAN experiments, the lasers were set to the following pulse durations and power densities. For Fig. 5i–k the 405 nm on-switch beam was set to 2 ms with a power density of 0.74 W/cm^2^, the 488 nm switch-off beam was set to 2 ms with a power density of 42.51 W/cm^2^, with the readout beam being 10 ms long with 6.16 W/ cm^2^. For the experiment with 50 frames per period (Fig. 5h), the settings were 6 ms for the 405 nm switch-on with 0.20 W/cm^2^, 1 ms for the 488 nm switch-off with 63 W/cm^2^ and 50 ms 488 nm readout with 1.08 W/cm^2^. For the experiments of Fig. 6v–ad, longer puls durations were tested with a 6 ms switch-on beam of 0.20 W/cm^2^, 72 ms switch-off beam of 5.31 W/cm^2^ and a 96 ms long readout duration with 0.64 W/cm^2^.

For the FrExPAN measurements, 700 frames were recorded. The ExPAN beam was added after 30–50 frames to be able to distinguish the ExPAN and the readout frame. For further data processing, only the camera frames recorded during the third readout laser pulses were taken, so the first 100 frames of raw data were cut out, resulting in 300 frames for the readout frames only. The FPM reference measurements were taken immediately after the FrExPAN acquisition was done with the same settings and intensities, except for the second ExPAN beam, which was shuttered.

The FrExPAN pulse scheme is more complicated than our original ExPAN approach^18^. However, one disadvantage of the deexcitation mechanism originally used for ExPAN are high intensities for efficient deexcitation and thus effective narrowing of the angle range of excited chromophores. This resulted in very fast photobleaching of the used dyes. Therefore, we did here use the photoswitching mechanism of RSFPs for deexcitation as this allows using much lower intensities. This allows for the observation of signals for longer periods of time, for example, to observe how structures of living cells change. In general, lower intensities also result in less phototoxicity.

### Software for data acquisition and image processing

Images were acquired using µManager software. Data processing was done with ImageJ. The FPM data were background-corrected using the plugin “stack mean normalization,” which normalizes the data by the mean of every single pixel over the length of the stack, followed by the usage of the plugin “avg periodic stack” to yield the phase average. The plugin averages all frames of the corresponding phase throughout the measurement, resulting in a 15-frame-long movie where each frame corresponds to a single averaged period of polarization orientation. The FFTs of the FPM data were done using a Python script which transformed each pixel along the temporal axis. Using the ImageJ plugin “frequency domain visualization,” each image pixel was assigned a color depending on the phase value of the FFT transformed image^35^.

For the data processing of the FPM vs FrExPAN measurements, the first 50 (Fig. 3 frames without the second ExPAN beam were cut out. Due to the cut, the absolute frame number for the polarization orientation during the FPM and FrExPAN measurements were not identical, and therefore the obtained data was phase corrected. After the cut, only the camera frame data observed during the third readout laser pulse were taken for further data processing. The images were background-corrected using the plugin “stack mean normalization”. The phase average stack was obtained using the plugin “avg periodic stack”. For the data of FrExPAN and NoExPAN in Fig. 6, again FFTs were done, followed by two-color-coding where the assigned color is a specified one (here: red or green) depending on a threshold value for the phase^35^.

### Software for statistical evaluation of ExPAN-Factors

The background-corrected raw data was analyzed by a Python script that was developed with the assistance of ChatGPT in order to evaluate the angle narrowing effect using two datasets of the same sample one with the ExPAN beam (ExPAN-stack) without the additional ExPAN beam (NoExPAN-stack).

The script first picks a specified number of ROIs of a quadratic size (x and y dimensions can be manually defined within the script) for the ExPAN-stack and using the fit function (Eq. (2)) in order to extract the angle narrowing or rather the ExPAN factor of the modulated fluorescence signal. The process is applied to the background-corrected and phase-averaged data which is additionally normalized by its intensity average before the start of ROI picking. The automatic ROI picking is then divided into two sequential steps, which happen based on a generated grid that starts the search for potential ROIs in the middle of the image and continues expanding outwards while ensuring that there is no overlap between chosen ROIs. After the grid has been determined, the script picks ROI candidates based on an intensity threshold and the integration of the Allen cell and structure segmenter^53^ to target primarily bright fluorescing structures of the cell membrane. In the next step, the script attempts a fit based on the intensity values using Eq. (2), and then checks the results against a defined *R*-squared threshold. That process is implemented to ensure sufficient fit quality and that the analysis of angle narrowing focuses primarily on regions with high fluorescence modulation, or, respectively, on regions with a significant dependency on the modulated polarization vector of the excitation light. The script continues to look for ROI candidates until the defined maximum number is reached or the whole image has been scanned. When the ROI picking process is completed the script saves a list of all ROIs with the coordinates (results for the shown datasets from Fig. 5 can be found in the supplementary) followed by the analyzation of the ExPAN-factors for both data stacks by applying the fit function and extracting the resulting factors for both datasets for each ROI. This is again applied to the background-corrected, phase-averaged and normalized stacks for ExPAN and NoExPAN. For the fitting of the NoExPAN-stack, there is another separate lower *R*-squared threshold applied, which is needed due to the circumstance that the modulation signal of the structures is much weaker and in order to prevent the calculation of unrealistic ExPAN factors as a result of poor fit quality. After all fits for the picked ROIs within both datasets have been carried out the script goes on to statistically evaluate the effect of the ExPAN narrowing by determining the Cohen’s d effect size and performing a two-sample independent *T*-test calculating the *T*-statistic and *P* value in order to show whether there is a statistically significant angle narrowing between the two stacks. For the final plotting of all results, the script offers the function to apply an automatic phase shift to the raw data and fit curves plot comparison between the two stacks. For the curve plots shown in Fig. 5, the phase shift has been applied. The resulting fits for all ROIs with comparison of the ExPAN and the NoExPAN Dataset can be exemplarily found for Fig. 5h in Supplementary Data 1.

### Deconvolution analysis by local polarization amplitude (ALPA) algorithm of actin fibers

The algorithm used for evaluation and deconvolution of the actin filament datasets uses the following equation to describe the fluctuating intensity I(t) in each pixel.

$$I(t)=I_0+A\cos (t)-B\sin (t)+\sqrt{ A^{2}+B^{2} }$$

The offset values *I*_0_ are set to zero if they would take negative values in any iteration. The individual pixels described by this three-parameter approach are then spatially blurred by the PSF to generate the recovered blurred data that is then compared to the measured raw data in a least-squares functional. This function is then minimized by the BFGS algorithm from Python’s scipy library17. The starting parameters are determined from the Fourier transforms (also from the scipy library) of the raw data in each pixel. Similarly, the amplitudes presented in this paper were generated by Fourier transforms. For the deconvolved data shown in this paper, ALPA 2400 iterations were done. We note that we could observe small differences in the obtained results when running the algorithm on Linux and Windows machines, probably due to numerical noise^35^.

### Deconvolution analysis by SPEED and Richardson–Lucy

For deconvolution of the FrExPAN and NoExPAN results displayed in Fig. 6 and in the Supplementary Movies 3, 4a, b, the SPEED and Richardson–Lucy algorithms were applied to the normalized and background-corrected phase averages. The SPEED algorithm was applied as described in previous publications^18,34^ with values for lambda parameters of λ_1_ = 0.20 and λ_2_ = 5.00 and 500 iterations. The Richardson–Lucy algorithm was applied using the DeconvolutionLab2 Plugin in ImageJ for 500 iterations.

## Supplementary information

Supplementary Information

Supplementary Movie 1a

Supplementary Movie 1b

Supplementary Movie 1c

Supplementary Movie 1d

Supplementary Movie 2

Supplementary Movie 3

Supplementary Movie 4a

Supplementary Movie 4b

## Data Availability

The data supporting the findings of this study are available within the article or its supplementary materials. Raw data of this study and software for statistical evaluation of the ExPAN-factors are available from the corresponding author, P.J.W., on request.

## Code availability

The data supporting the findings of this study are available within the article or its supplementary materials. Raw data of this study and software for statistical evaluation of the ExPAN-factors are available from the corresponding author, P.J.W., on request.

## References

[1] Camacho, R., Täuber, D. & Scheblykin, I. G. Fluorescence Anisotropy Reloaded-Emerging Polarization Microscopy Methods for Assessing Chromophores’ Organization and Excitation Energy Transfer in Single Molecules, Particles, Films, and Beyond. *Adv. Mater.* **31**, e1805671 (2019). 30721532 10.1002/adma.201805671

[2] Brasselet, S. Fluorescence polarization modulation super-resolution imaging provides refined dynamics orientation processes in biological samples. *Light Sci. Appl.* **11**, 322 (2022). 36336677 10.1038/s41377-022-01018-w PMC9637731

[3] Chen, L. et al. Advances of super-resolution fluorescence polarization microscopy and its applications in life sciences. *Comput. Struct. Biotechnol. J.* **18**, 2209–2216 (2020). 32952935 10.1016/j.csbj.2020.06.038 PMC7476067

[4] Lazar, J., Bondar, A., Timr, S. & Firestein, S. J. Two-photon polarization microscopy reveals protein structure and function. *Nat. Methods* **8**, 684–690 (2011). 21725301 10.1038/nmeth.1643

[5] Kress, A. et al. Mapping the local organization of cell membranes using excitation-polarization-resolved confocal fluorescence microscopy. *Biophys. J.* **105**, 127–136 (2013). 23823231 10.1016/j.bpj.2013.05.043 PMC3699755

[6] Forkey, J. N., Quinlan, M. E., Shaw, M. A., Corrie, J. E. T. & Goldman, Y. E. Three-dimensional structural dynamics of myosin V by single-molecule fluorescence polarization. *Nature* **422**, 399–404 (2003). 12660775 10.1038/nature01529

[7] Mehta, S. B. et al. Dissection of molecular assembly dynamics by tracking orientation and position of single molecules in live cells. *Proc. Natl Acad. Sci. USA* **113**, E6352–E6361 (2016). 27679846 10.1073/pnas.1607674113 PMC5081662

[8] Valades Cruz, C. A. et al. Quantitative nanoscale imaging of orientational order in biological filaments by polarized superresolution microscopy. *Proc. Natl Acad. Sci. USA* **113**, E820–8 (2016). 26831082 10.1073/pnas.1516811113 PMC4763786

[9] Piston, D. W. & Rizzo, M. A. FRET by fluorescence polarization microscopy. *Methods Cell Biol.* **85**, 415–430 (2008). 10.1016/S0091-679X(08)85018-2 18155473

[10] Brasselet, S. Polarization-resolved microscopy in the life sciences. *Opt. Photonics News* **30**, 34–41 (2019).

[11] Brasselet, S. & Alonso, M. A. Polarization microscopy: from ensemble structural imaging to single-molecule 3D orientation and localization microscopy. *Optica* **10**, 1486 (2023).

[12] Thomsson, D., Lin, H. & Scheblykin, I. G. Correlation analysis of fluorescence intensity and fluorescence anisotropy fluctuations in single-molecule spectroscopy of conjugated polymers. *ChemPhysChem* **11**, 897–904 (2010). 20087921 10.1002/cphc.200900724

[13] Wazawa, T. et al. Highly biocompatible super-resolution fluorescence imaging using the fast photoswitching fluorescent protein Kohinoor and SPoD-ExPAN with Lp-regularized image reconstruction. *Microscopy* **67**, 89–98 (2018). 29409007 10.1093/jmicro/dfy004

[14] Wazawa, T. et al. A photoswitchable fluorescent protein for hours-time-lapse and sub-second-resolved super-resolution imaging. *Microscopy* **70**, 340–352 (2021). 33481018 10.1093/jmicro/dfab001 PMC8350982

[15] Parasassi, T., de Stasio, G., d’Ubaldo, A. & Gratton, E. Phase fluctuation in phospholipid membranes revealed by Laurdan fluorescence. *Biophys. J.* **57**, 1179–1186 (1990). 2393703 10.1016/S0006-3495(90)82637-0 PMC1280828

[16] Parasassi, T., Stasio, G., de, Ravagnan, G., Rusch, R. M. & Gratton, E. Quantitation of lipid phases in phospholipid vesicles by the generalized polarization of Laurdan fluorescence. *Biophys. J.* **60**, 179–189 (1991). 1883937 10.1016/S0006-3495(91)82041-0 PMC1260049

[17] Jha, K. K. et al. A BODIPY-based molecular rotor in giant unilamellar vesicles: a case study by polarization-resolved time-resolved emission and transient absorption spectroscopy. *ChemPhotoChem* **7**, e202300091 (2023).

[18] Hafi, N. et al. Fluorescence nanoscopy by polarization modulation and polarization angle narrowing. *Nat. Methods* **11**, 579–584 (2014). 24705472 10.1038/nmeth.2919

[19] Zhanghao, K. et al. Super-resolution imaging of fluorescent dipoles via polarized structured illumination microscopy. *Nat. Commun.* **10**, 4694 (2019). 31619676 10.1038/s41467-019-12681-w PMC6795901

[20] Kabbani, A. M. & Kelly, C. V. The detection of nanoscale membrane bending with polarized localization microscopy. *Biophys. J.* **113**, 1782–1794 (2017). 29045872 10.1016/j.bpj.2017.07.034 PMC5647545

[21] Liu, X. et al. Perpendicular alignment of 2D nanoplatelet emitters in electrospun fibers: a result of the Barus effect?. *Macro Mater. Eng.* **308**, 2300027 (2023).

[22] Liu, X., Gädeke, F., Hohgardt, M. & Walla, P. J. Highly efficient and stable luminescent solar concentrator based on light-harvesting and energy-funneling nanodot pools feeding aligned, light-redirecting nanorods. *Sol. RRL* **8**, 2400273 (2024).

[23] Pieper, A. et al. Biomimetic light-harvesting funnels for re-directioning of diffuse light. *Nat. Commun.* **9**, 666 (2018). 29445168 10.1038/s41467-018-03103-4 PMC5812990

[24] Willich, M. M. et al. A new ultrafast energy funneling material harvests three times more diffusive solar energy for GaInP photovoltaics. *Proc. Natl Acad. Sci. USA* **117**, 32929–32938 (2020). 33318220 10.1073/pnas.2019198117 PMC7776598

[25] Abrahamsson, S. et al. MultiFocus polarization microscope (MF-PolScope) for 3D polarization imaging of up to 25 focal planes simultaneously. *Opt. Express, OE* **23**, 7734–7754 (2015). 10.1364/OE.23.007734 PMC5802244 25837112

[26] Loman, A., Gregor, I., Stutz, C., Mund, M. & Enderlein, J. Measuring rotational diffusion of macromolecules by fluorescence correlation spectroscopy. *Photochem. Photobio. Sci.* **9**, 627–636 (2010). 10.1039/b9pp00029a 20442920

[27] Backlund, M. P. et al. Simultaneous, accurate measurement of the 3D position and orientation of single molecules. *Proc. Natl Acad. Sci. USA* **109**, 19087–19092 (2012). 23129640 10.1073/pnas.1216687109 PMC3511094

[28] DeMay, B. S., Noda, N., Gladfelter, A. S. & Oldenbourg, R. Rapid and quantitative imaging of excitation polarized fluorescence reveals ordered septin dynamics in live yeast. *Biophys. J.* **101**, 985–994 (2011). 21843491 10.1016/j.bpj.2011.07.008 PMC3175061

[29] Zhanghao, K. et al. Super-resolution dipole orientation mapping via polarization demodulation. *Light Sci. Appl.* **5**, e16166 (2016). 30167126 10.1038/lsa.2016.166 PMC6059828

[30] Shaban, H. A., Valades-Cruz, C. A., Savatier, J. & Brasselet, S. Polarized super-resolution structural imaging inside amyloid fibrils using thioflavine T. *Sci. Rep.* **7**, 12482 (2017). 28970520 10.1038/s41598-017-12864-9 PMC5624930

[31] Curcio, V., Alemán-Castañeda, L. A., Brown, T. G., Brasselet, S. & Alonso, M. A. Birefringent Fourier filtering for single molecule coordinate and height super-resolution imaging with dithering and orientation. *Nat. Commun.* **11**, 5307 (2020). 33082309 10.1038/s41467-020-19064-6 PMC7576605

[32] Benninger, R. K. P., Onfelt, B., Neil, M. A. A., Davis, D. M. & French, P. M. W. Fluorescence imaging of two-photon linear dichroism: cholesterol depletion disrupts molecular orientation in cell membranes. *Biophys. J.* **88**, 609–622 (2005). 15520272 10.1529/biophysj.104.050096 PMC1305038

[33] Liu, Q. et al. Self-alignment of dye molecules in micelles and lamellae for three-dimensional imaging of lyotropic liquid crystals. *Langmuir* **27**, 7446–7452 (2011). 21598933 10.1021/la200842z

[34] Hafi, N. et al. Reply to “Polarization modulation adds little additional information to super-resolution fluorescence microscopy”. *Nat. Methods* **13**, 8–9 (2016). 26716557 10.1038/nmeth.3721

[35] Albrecht, A. et al. Amplitude analysis of polarization modulation data and 3D-polarization demodulation (3D-SPoD). Preprint at *bioRxiv*10.1101/2020.03.10.986034 (2020).

[36] Wulf, E., Deboben, A., Bautz, F. A., Faulstich, H. & Wieland, T. Fluorescent phallotoxin, a tool for the visualization of cellular actin. *Proc. Natl Acad. Sci. USA* **76**, 4498–4502 (1979). 291981 10.1073/pnas.76.9.4498 PMC411604

[37] Kage, F. et al. FMNL formins boost lamellipodial force generation. *Nat. Commun.* **8**, 14832 (2017). 28327544 10.1038/ncomms14832 PMC5364437

[38] Oda, T., Namba, K. & Maéda, Y. Position and orientation of phalloidin in F-actin determined by X-ray fiber diffraction analysis. *Biophys. J.* **88**, 2727–2736 (2005). 15653738 10.1529/biophysj.104.047753 PMC1305368

[39] Kinosita, K. et al. in *Mechanism of Myofilament Sliding in Muscle Contraction* (ed. Sugi, H.) (Springer, 1993).

[40] Parasassi, T., Gratton, E., Yu, W. M., Wilson, P. & Levi, M. Two-photon fluorescence microscopy of laurdan generalized polarization domains in model and natural membranes. *Biophys. J.* **72**, 2413–2429 (1997). 9168019 10.1016/S0006-3495(97)78887-8 PMC1184441

[41] Sanchez, S. A., Tricerri, M. A. & Gratton, E. Laurdan generalized polarization fluctuations measures membrane packing micro-heterogeneity in vivo. *Proc. Natl Acad. Sci. USA* **109**, 7314–7319 (2012). 22529342 10.1073/pnas.1118288109 PMC3358851

[42] Gunther, G., Malacrida, L., Jameson, D. M., Gratton, E. & Sánchez, S. A. LAURDAN since Weber: the quest for visualizing membrane heterogeneity. *Acc. Chem. Res.* **54**, 976–987 (2021). 33513300 10.1021/acs.accounts.0c00687 PMC8552415

[43] Badley, R. A., Martin, W. G. & Schneider, H. Dynamic behavior of fluorescent probes in lipid bilayer model membranes. *Biochemistry* **12**, 268–275 (1973). 4683001 10.1021/bi00726a015

[44] Kawato, S., Kinosita, K. & Ikegami, A. Dynamic structure of lipid bilayers studied by nanosecond fluorescence techniques. *Biochemistry* **16**, 2319–2324 (1977). 577184 10.1021/bi00630a002

[45] Tiwari, D. K. et al. A fast- and positively photoswitchable fluorescent protein for ultralow-laser-power RESOLFT nanoscopy. *Nat. Methods* **12**, 515–518 (2015). 25894946 10.1038/nmeth.3362

[46] Duwé, S. et al. Expression-enhanced fluorescent proteins based on enhanced green fluorescent protein for super-resolution microscopy. *ACS Nano* **9**, 9528–9541 (2015). 26308583 10.1021/acsnano.5b04129

[47] Grunwald, M. *Molecular Orientation as Contrast Mechanism for Fluorescence Microcopy and Confocal Multidetector-Scanning-Microscopy*. Dissertation, Universität Göttingen (2015).

[48] Jess, L. S. *Fluorescence Microscopy for Interference Lithography: Set-up Design and Pattern Characterization by Fluorescence Modulation*. Dissertation, Technische Universität Braunschweig (2017).

[49] Hell, S. W. & Wichmann, J. Breaking the diffraction resolution limit by stimulated emission: stimulated-emission-depletion fluorescence microscopy. *Opt. Lett.* **19**, 780–782 (1994). 19844443 10.1364/ol.19.000780

[50] Konen, T. et al. The positive switching fluorescent protein padron2 enables live-cell reversible saturable optical linear fluorescence transitions (RESOLFT) nanoscopy without sequential illumination steps. *ACS Nano* **15**, 9509–9521 (2021). 34019380 10.1021/acsnano.0c08207 PMC8291764

[51] Mishra, K. et al. Genetically encoded photo-switchable molecular sensors for optoacoustic and super-resolution imaging. *Nat. Biotechnol.* **40**, 598–605 (2022). 34845372 10.1038/s41587-021-01100-5 PMC9005348

[52] Mullineaux, C. W., Nenninger, A., Ray, N. & Robinson, C. Diffusion of green fluorescent protein in three cell environments in *Escherichia coli*. *J. Bacteriol.* **188**, 3442–3448 (2006). 16672597 10.1128/JB.188.10.3442-3448.2006 PMC1482841

[53] Chen, J. et al. The Allen cell and structure segmenter: a new open source toolkit for segmenting 3D intracellular structures in fluorescence microscopy images. Preprint at *bioRxiv*10.1101/491035 (2018).

