# Supplementary material for: High contrast fluorescence polarization microscopy through double tagged photoswitchable fluorescent proteins
Source: Npj Imaging. 2025 Jul 2;3:31. doi: 10.1038/s44303-025-00094-y (PMC12222721; doi:10.1038/s44303-025-00094-y)
Supplement: Supplementary file 1 — Supplementary Information [file 44303_2025_94_MOESM1_ESM.pdf]

Supplementary Figure 1

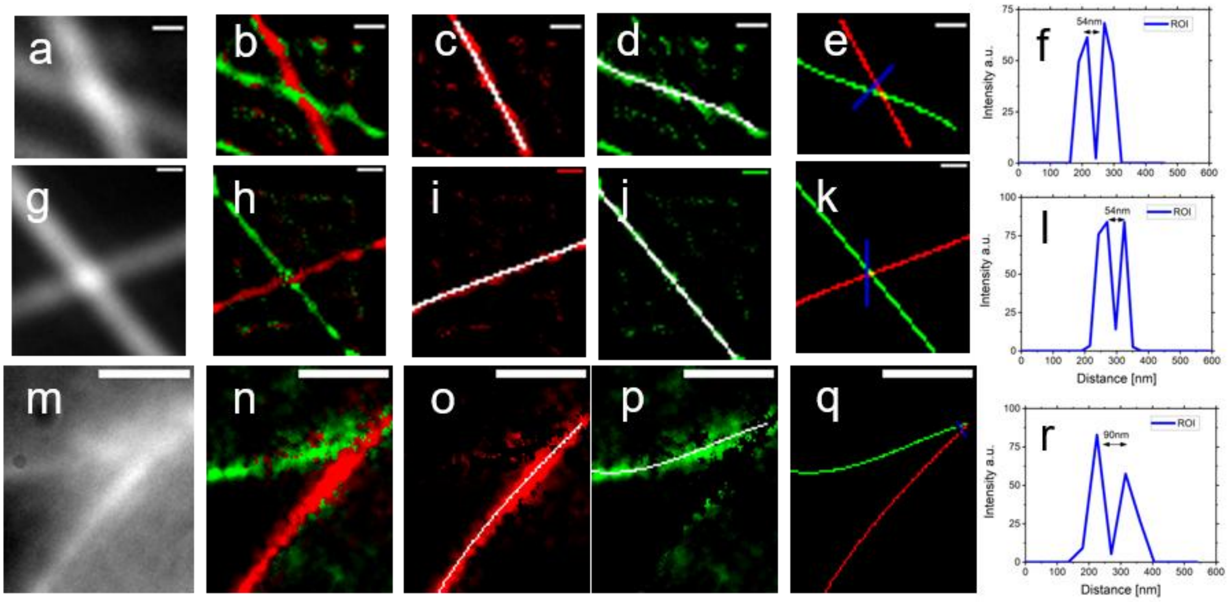

Supplementary Figure 1: a), g), m) Diffraction limited Images. b), h), n) deconvolved and reconstructed phase seperation with color coding of selected phase/orientation ranges from the amplitude and phase data. c)-d), i)-j), o)-p) Seperate depiciption of the red and green colored phase with the fit results after spline interpolation (white line). Based on the intensity values from the seperate colour coded image a local thickness analysis was applied first followed by spline interpolation and fitting of a polynomial degree 3 function. e), k), q) Colour coded results of the spline interpolation fit with located ROI (blue). f), l), r) Intensity profiles of the along the corresponding ROIs. Scale bars a)-k) 0,25 µm, m)-q) 2 µm.

Supplementary Movie 1a

Supplementary Movie 1a Comparison of single (a-e) and double (f-j) membrane tagged RSFPs. FPM results from (b) the st-Kohinoor-F, (g) dt-p-Kohinoor-F, , (c) st-p-rsGreenF, (d) st-rsGreenF-F (h-j) dt-p-rsGreenF-F. Shown is the background corrected phase averaged modulation signal and the corresponding background corrected modulation data. Scale bars 2µm.

Supplementary Movie 1b

Principle of single (a) and double (d) membrane tagged RSFPs. FPM results from (b) living HeLa-cell samples expressing the st-Kohinoor-F, (c) HeLa cell samples expressing p-rsGreenF, (e) Hela cells expressing the dt-p-Kohinoor-F, (f) HeLa cell expressing the dt-p-rsGreenF-F. Shown is the background corrected phase averaged colored FFT image (right) with the corresponding background corrected phase averaged modulation signal (left). The phase averaged data consists of 15 frames with each resulting from an average of 10 (c and f) and 20 (b and e) images with the same phase. Excitation wavelength were 488nm with 2.44 W/cm^2 for Kohinoor and 405 nm with 0.1 W/cm^2 and 488 nm with 1.12 W/cm^2 for rsGreenF samples Scale bars 2µm.

Supplementary Movie 1c

Principle of single (a) and double (d) membrane tagged RSFPs. FPM results from (b) living HeLa-cell samples expressing the st-p-rsGreenF, (c) HeLa cell samples expressing rsGreenF-F, (e) Hela cells expressing the dt-p-Kohinoor-F, (f) HeLa cell expressing the dt-p-rsGreenF-. Shown is the background corrected phase averaged colored FFT image (right) phase averaged modulation signal (left). The phase averaged data consists of 15 (b and e) and 30 frames (c and f) with each resulting from an average of 20 (b and e) and 23 (c and f) images with the same phase. Excitation wavelengths were and 405 nm with 0.1 W/cm^2 and 488 nm with 1.12 W/cm^2 for rsGreenF samples Scale bars 2µm

Supplementary Movie 1d

Principle of single (a) and double (d) membrane tagged RSFPs. FPM results from living hippocampal neuron (b, c) expressing the st-rsGreenF-F, (e, f) expressing the dt-p-rsGreenF-F.Shown is the background corrected phase averaged colored FFT image (right) with the corresponding background corrected phase averaged modulation signal (left). The phase averaged data consists of 15 frames with each resulting from an average of 10 (c) and 20 (b, e-f) images with the same phase. Excitation wavelengths were and 405 nm with 0.1 W/cm^2 and 488 nm with 1.12 W/cm^2 for rsGreenF samples Scale bars 2µm.

Supplementary Movie 2

(a-g) Principle of frame separated excitation polarization angle narrowing (FrExPAN) for the usage with negative switching dt-rsFPs. (h-k) experimental FrExPAN Data of dt-p-rsGreenF-F expressing HeLa Cells (h, I, k) and hippocampal Neurons (j). (red, ExPAN) and without 2nd FrExPAN pulse (black, noExPAN) with displayed phase averaged background corrected modulation data. Scale bars 2µm.

Supplementary Movie 3

(a) FPM -Setup. (b) Diffraction limited data of single molecules and (c) false color version based on the different orientations. (d) Deconvolved results with SPEED algorithm. (e) Two selected phase ranges (f-t) of linear phalloidin-Atto 590 labelled actin filaments. (f) averaged raw data (k, p) orientation color coded (g, l, q) Deconvolved images based on the entire signal and (h, m, r) based on only the modulating part and (I, j, n, o, s, t) selected phase/ orientatiton ranges using ALPA algorithm. (u – ad). Data of a double membrane anchored dt-p rsGreenF-F transfected living HeLa cell without 2nd FrExPAN pulse (NoExpan) and (af – an) with FrExPAN (u, z, aj) averaged diffraction limited raw data. (v,aa, af, ak) deconvolved and orientation color coded results after 500 iterations with Richardson-Lucy. (w-y, ab-ad, ag-ai, al-an) deconvolved and orientation color coded images after 500 iterations with SPEED algorithm (ae) FrExPAN Set-up. Scale bars (b-e) 1µm, (f-t) 0,25µm and (u-an) 2µm. (b-d) and (f, k, p) previously published Hafi, N. et al. Reply to "Polarization modulation adds little additional information to super-resolution fluorescence microscopy". *Nature methods* 13, *8–9* (2016*)*

Supplementary Movie 4a

Results of experimental FrExPAN (a-h) and NoExPAN (i-p, without 2nd FrExPAN pulse) Data of HeLa Cells expressing dt-p-rsGreenF-F. Displayed is the whole Image (a-d and i-l) and a smaller region of interest where the cellmembranes come closer together (e-h, m-p) Shown is the background corrected and phase averaged raw data (a, e, i, m), only the modulation signal of the phase averaged data (b, f, j, n), deonconvolved results using the Richardson Lucy algorithm after 500 iterations (c, g, k, o) and reconstructed phase seperation with color coding of selected phase/orientation ranges from the amplitude and phase data (d, h , l, p). The reconstruction was done by shifting the absoluted selected phase equally across 60 images. Scale bars 2µm.

Supplementary Movie 4b

Results of experimental FrExPAN (a-h) and NoExPAN (i-p, without 2nd FrExPAN pulse) Data of HeLa Cells expressing dt-p-rsGreenF-F. Displayed is the whole Image (a-d and i-l) and a smaller region of interest where the cellmembranes come closer together (e-h, m-p) Shown is the background corrected and phase averaged raw data (a, e, i, m), only the modulation signal of the phase averaged data (b, f, j, n), deonconvolved results using the SPEED algorithm after 500 iterations with λ_1 = 0,20 and λ_2 = 5,00 (c, g, k, o) and reconstructed phase seperation with color coding of selected phase/orientation ranges from the amplitude and phase data (d, h , l, p). The reconstruction was done by shifting the absoluted selected phase equally across 30 images. Scale bars 2µm.

Supplementary Data 1

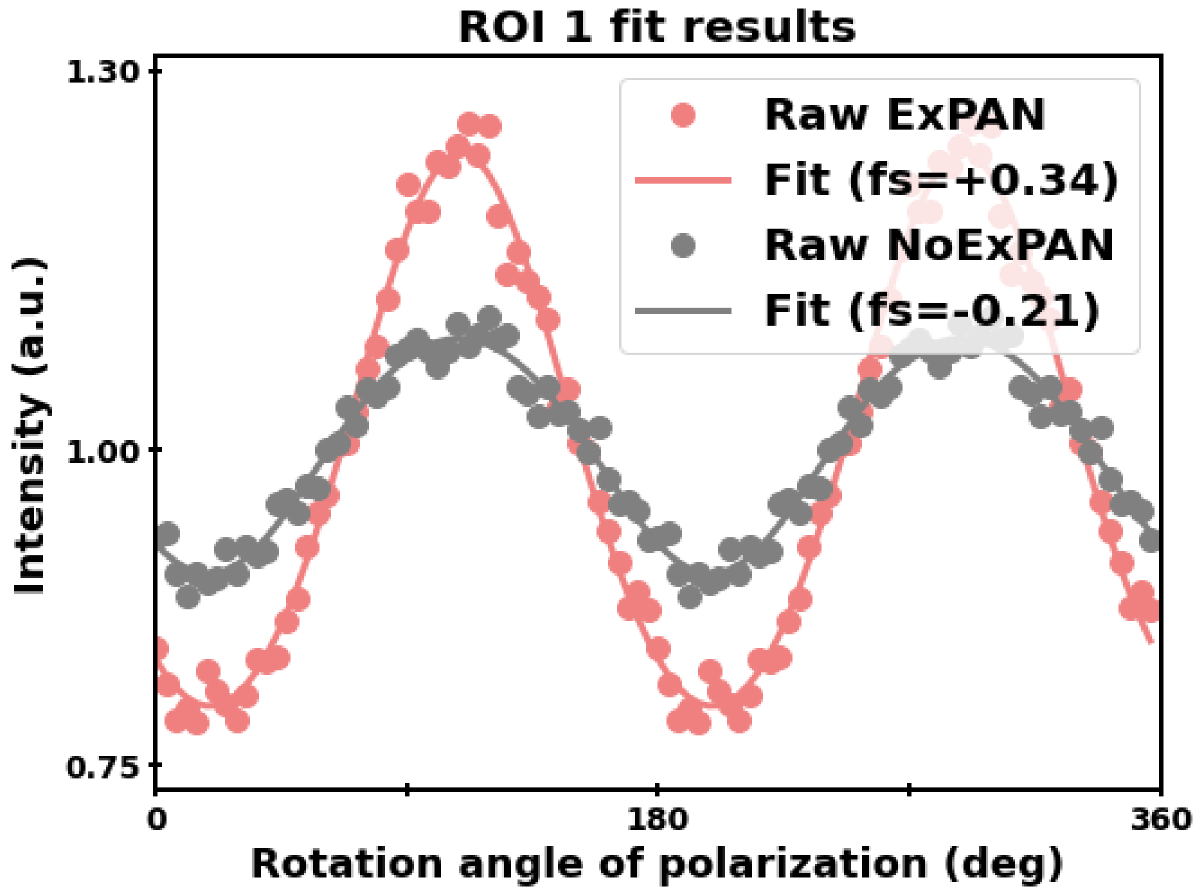

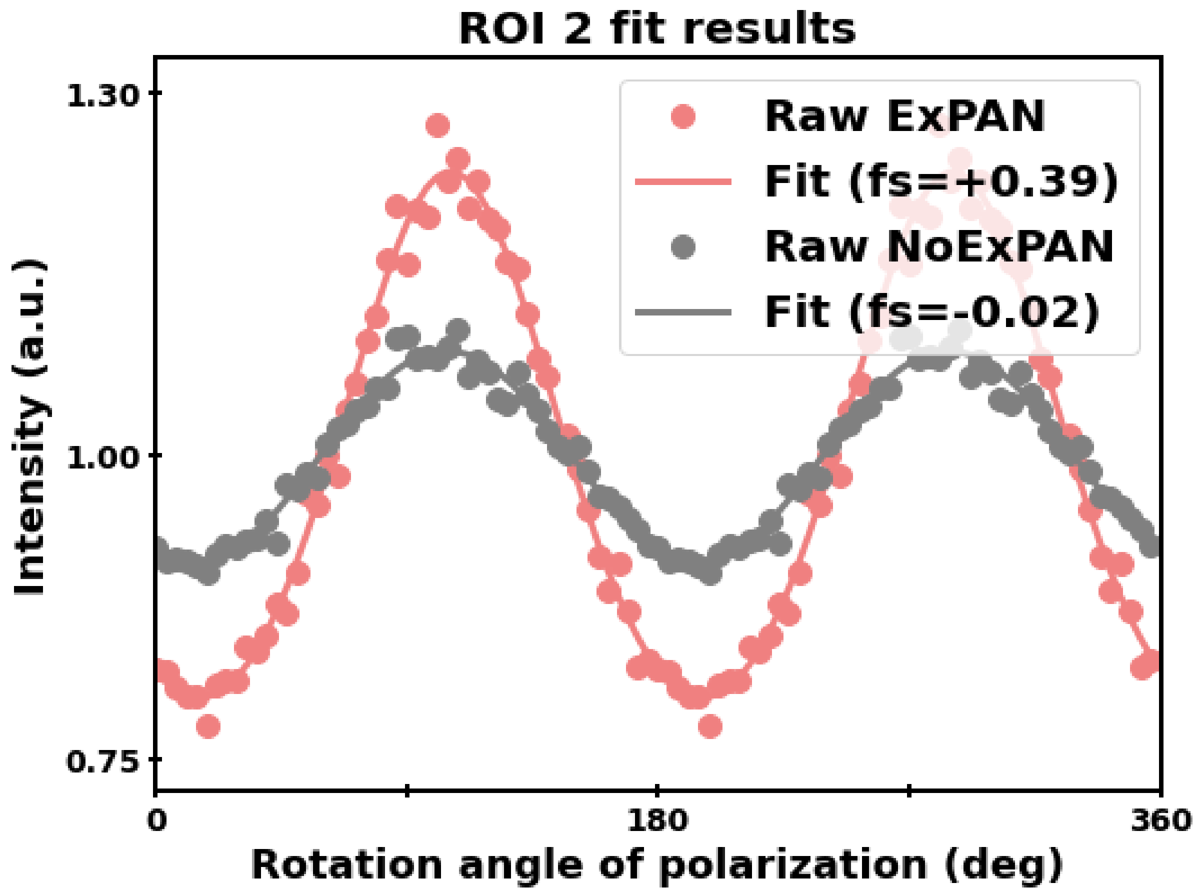

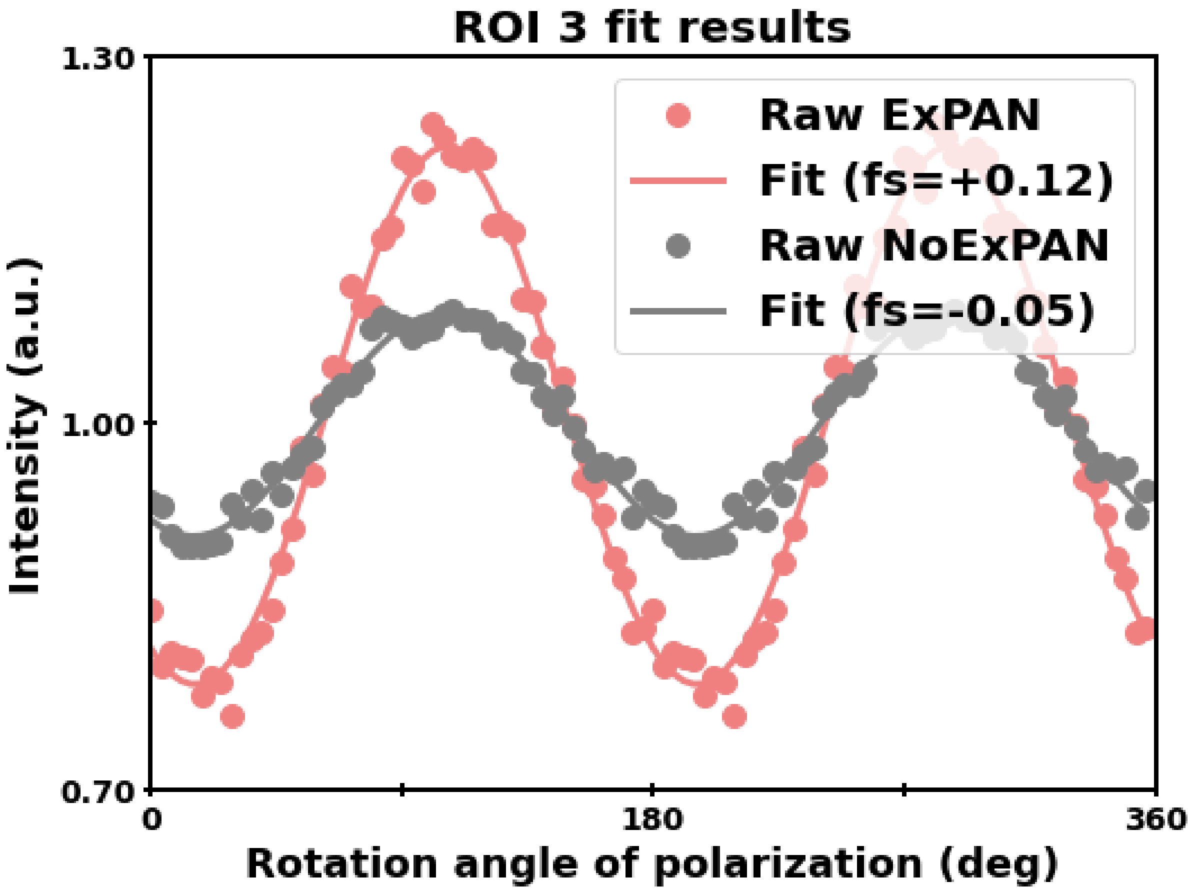
ROI 3 fit results
1.30
1.00
0.70
0
180
360
Intensity (a.u.)
Rotation angle of polarization (deg)
Raw ExPAN
Fit (fs=+0.12)
Raw NoExPAN
Fit (fs=-0.05)

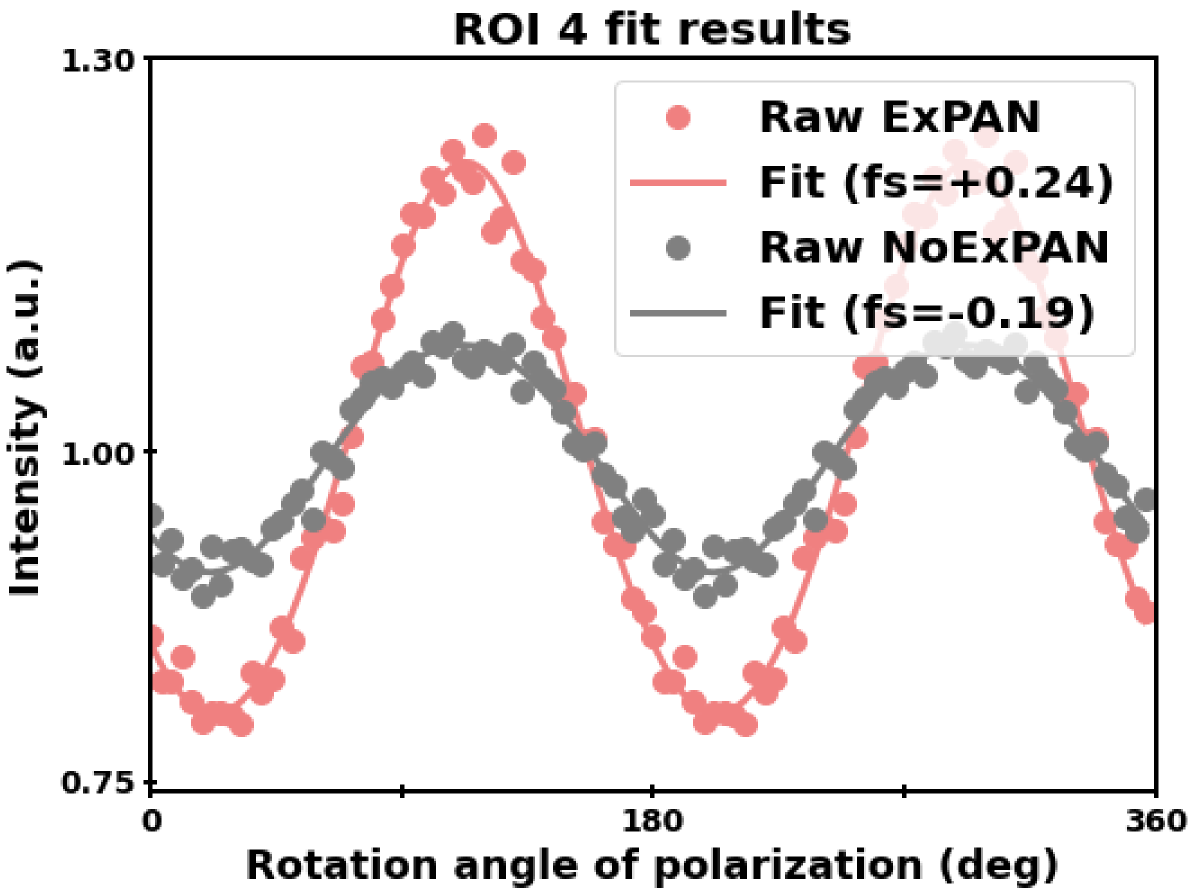
ROI 4 fit results
1.30
1.00
0.75
0
180
360
Intensity (a.u.)
Rotation angle of polarization (deg)
Raw ExPAN
Fit (fs=+0.24)
Raw NoExPAN
Fit (fs=-0.19)

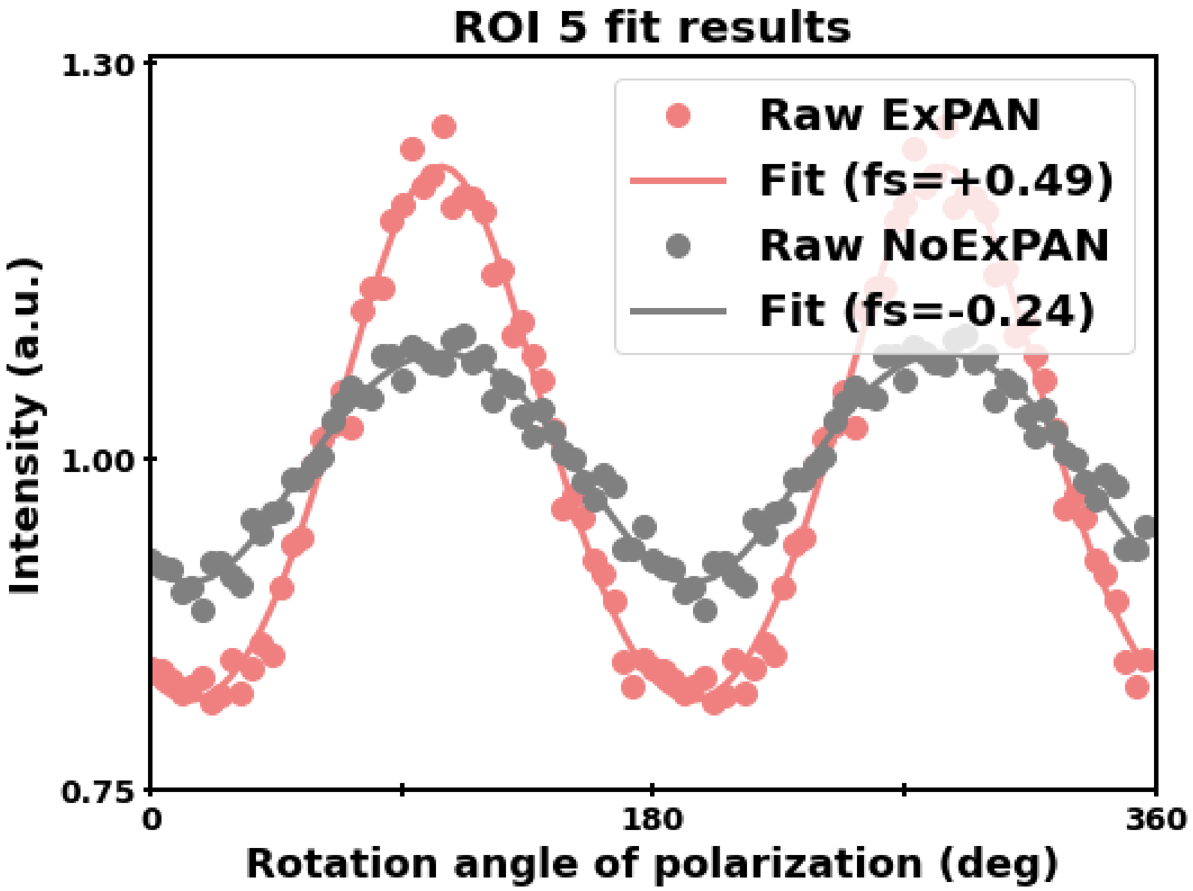
ROI 5 fit results
Raw ExPAN
Fit (fs=+0.49)
Raw NoExPAN
Fit (fs=-0.24)
Intensity (a.u.)
1.30
1.00
0.75
0
180
360
Rotation angle of polarization (deg)

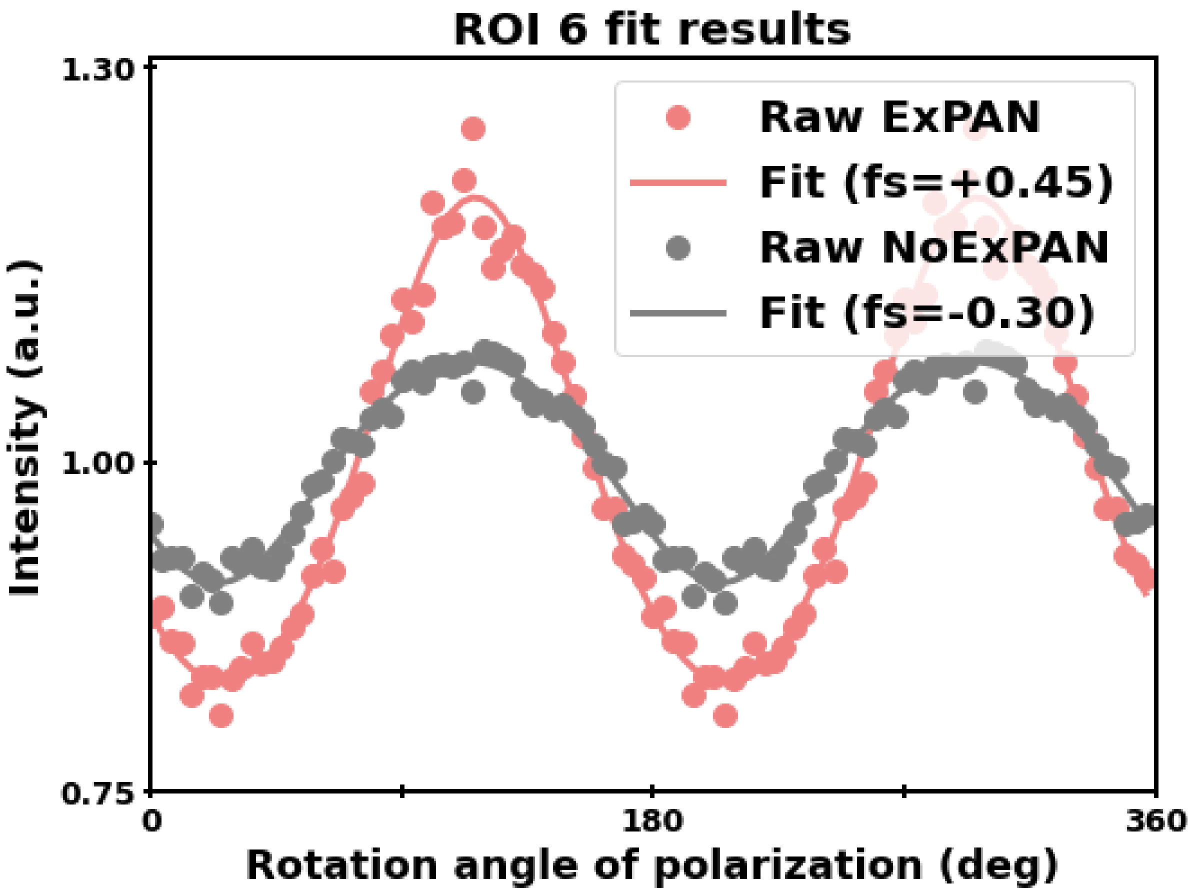
ROI 6 fit results
Raw ExPAN
Fit (fs=+0.45)
Raw NoExPAN
Fit (fs=-0.30)
Intensity (a.u.)
1.30
1.00
0.75
0
180
360
Rotation angle of polarization (deg)

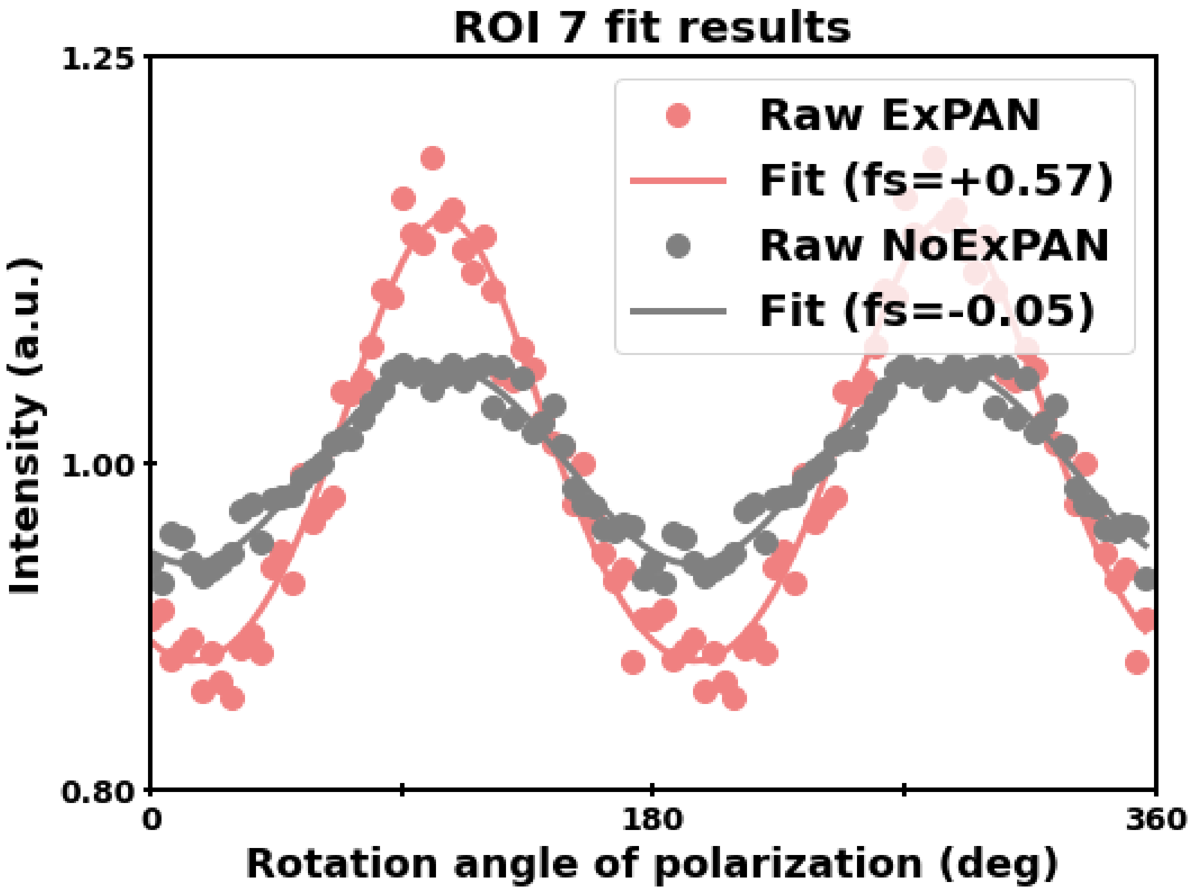
ROI 7 fit results
1.25
1.00
0.80
0
180
360
Intensity (a.u.)
Rotation angle of polarization (deg)
Raw ExPAN
Fit (fs=+0.57)
Raw NoExPAN
Fit (fs=-0.05)

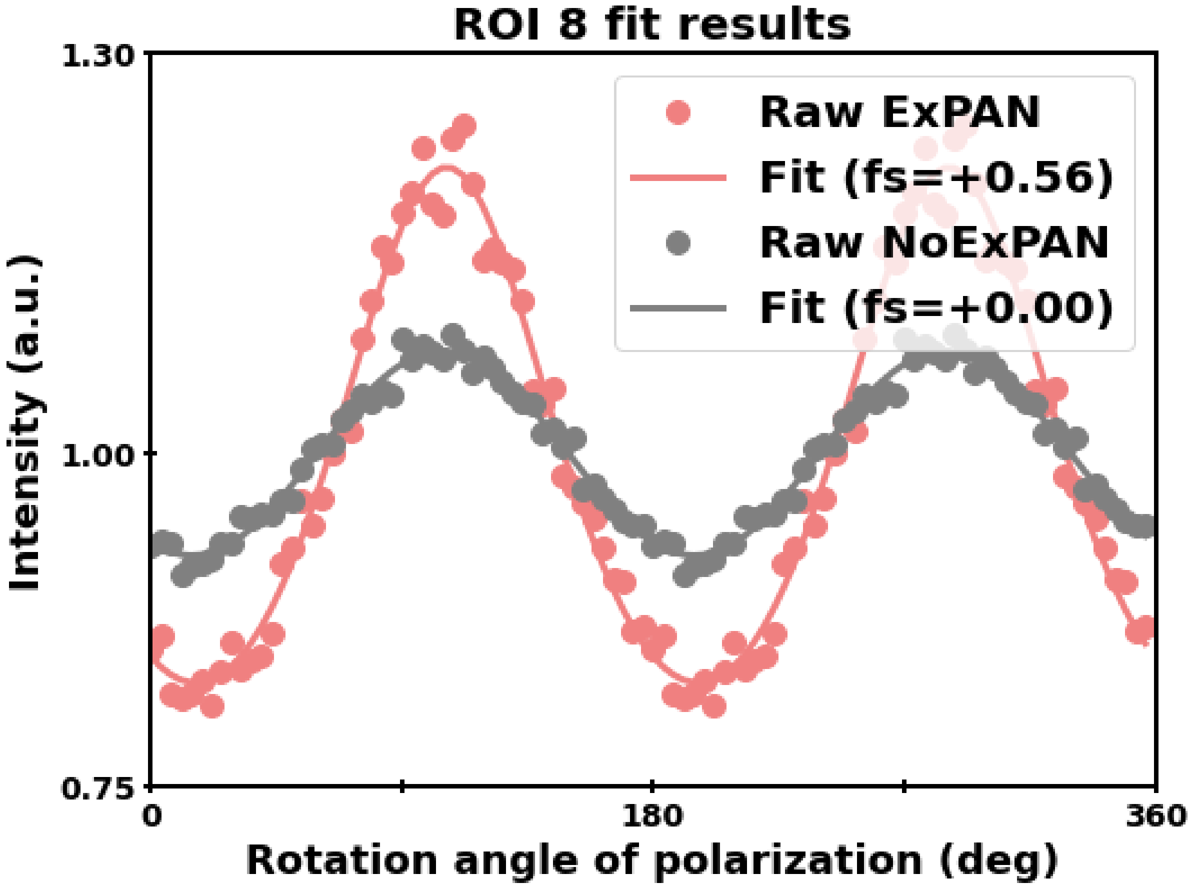
ROI 8 fit results
1.30
1.00
0.75
0
180
360
Intensity (a.u.)
Rotation angle of polarization (deg)
Raw ExPAN
Fit (fs=+0.56)
Raw NoExPAN
Fit (fs=+0.00)

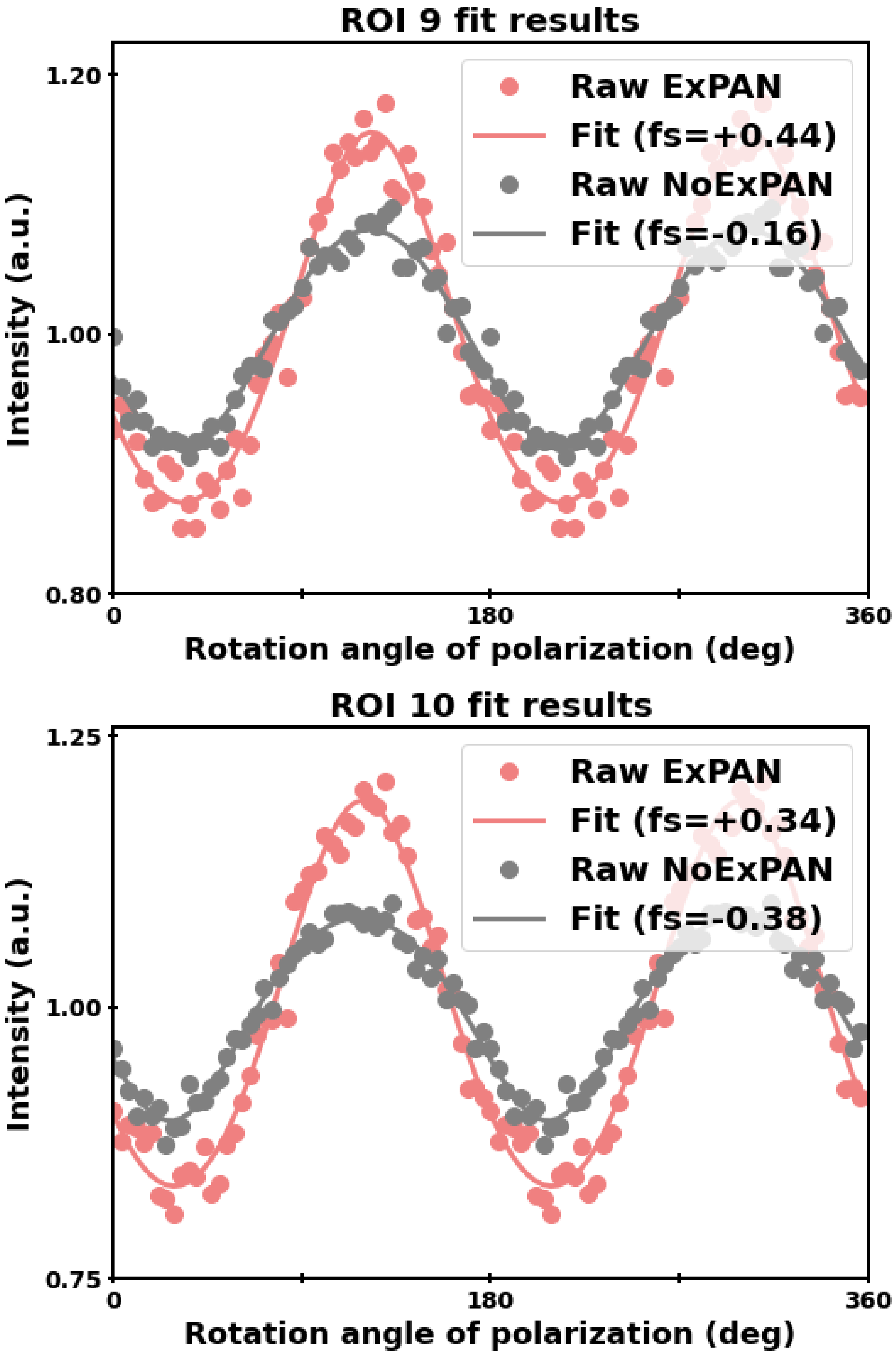
ROI 9 fit results
Raw ExPAN
Fit (fs=+0.44)
Raw NoExPAN
Fit (fs=-0.16)
1.20
1.00
0.80
Intensity (a.u.)
0
180
360
Rotation angle of polarization (deg)
ROI 10 fit results
Raw ExPAN
Fit (fs=+0.34)
Raw NoExPAN
Fit (fs=-0.38)
1.25
1.00
0.75
Intensity (a.u.)
0
180
360
Rotation angle of polarization (deg)

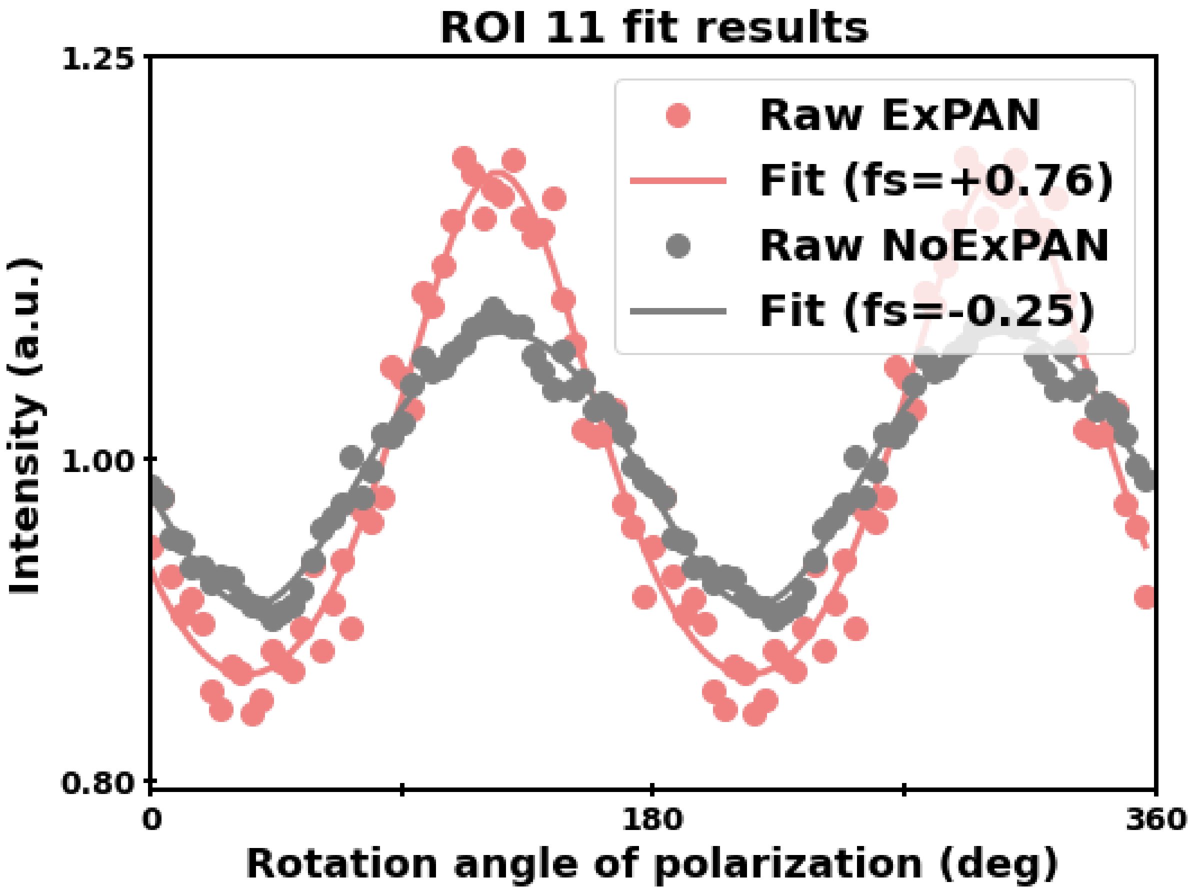

ROI 11 fit results
1.25
1.00
0.80
Intensity (a.u.)
0
180
360
Rotation angle of polarization (deg)
Raw ExPAN
Fit (fs=+0.76)
Raw NoExPAN
Fit (fs=-0.25)

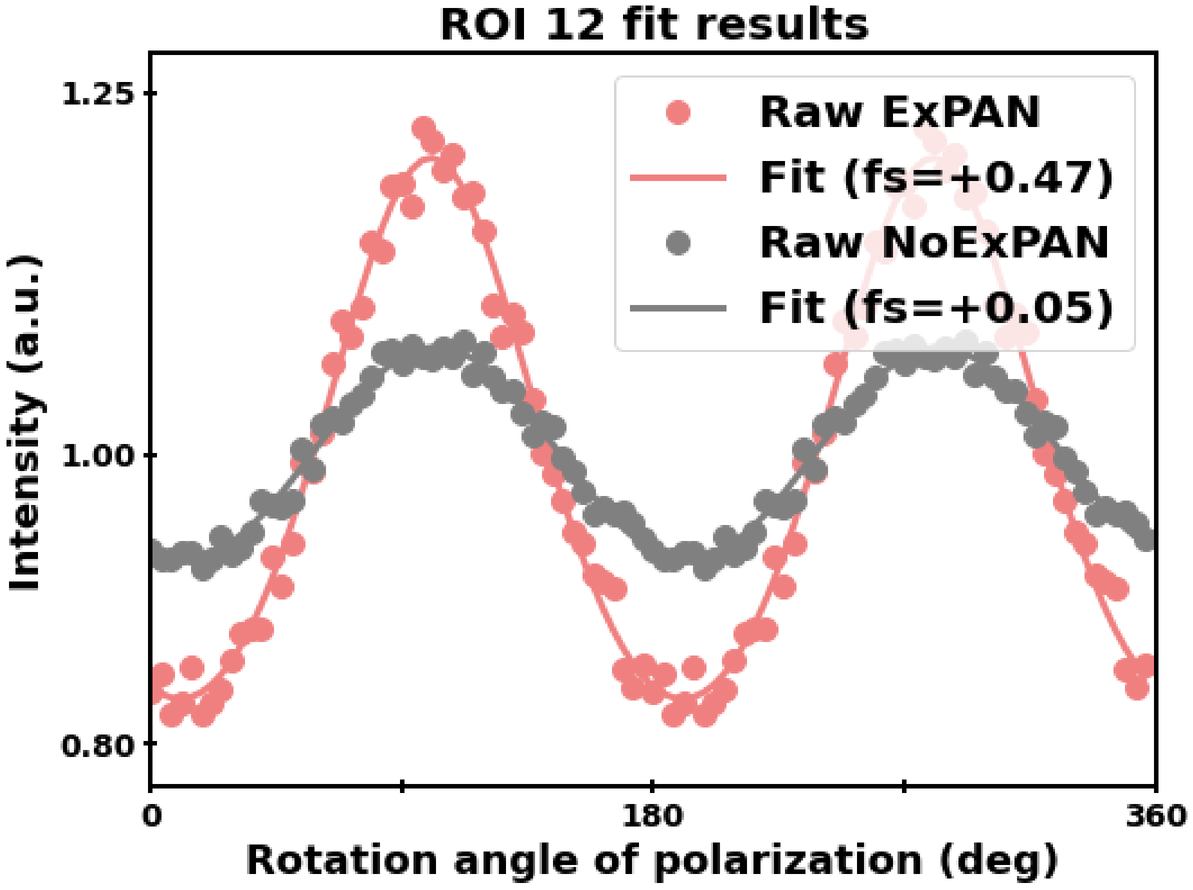

ROI 12 fit results
1.25
1.00
0.80
Intensity (a.u.)
0
180
360
Rotation angle of polarization (deg)
Raw ExPAN
Fit (fs=+0.47)
Raw NoExPAN
Fit (fs=+0.05)

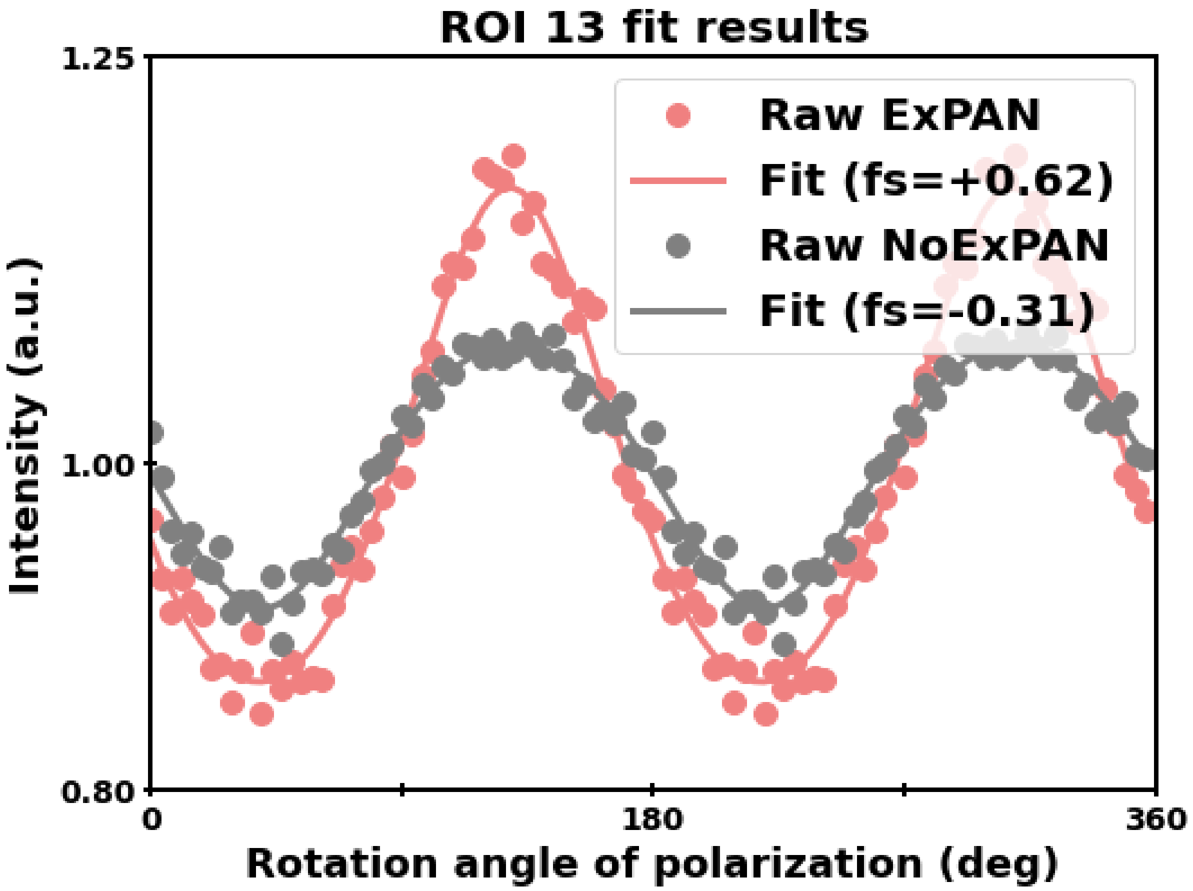
ROI 13 fit results
Raw ExPAN
Fit (fs=+0.62)
Raw NoExPAN
Fit (fs=-0.31)
Intensity (a.u.)
1.25
1.00
0.80
0
180
360
Rotation angle of polarization (deg)

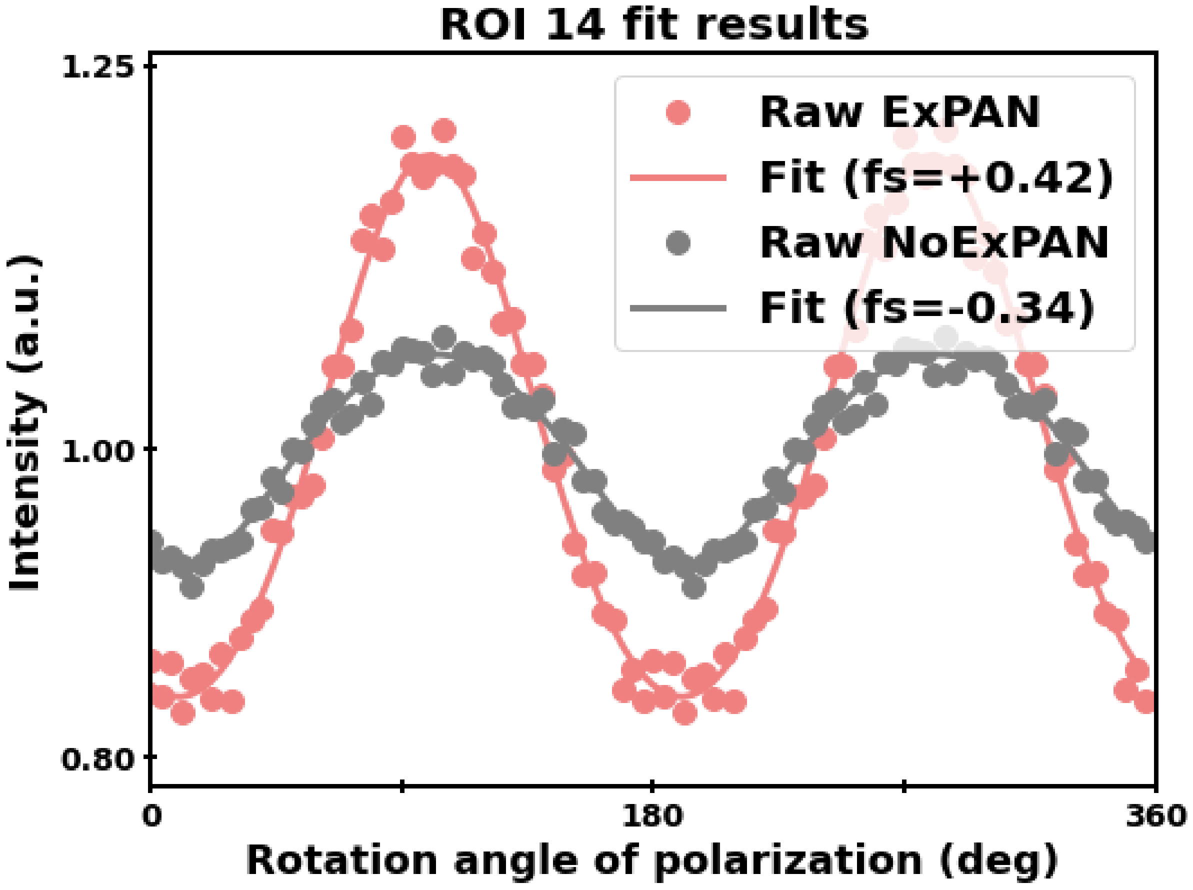
ROI 14 fit results
Raw ExPAN
Fit (fs=+0.42)
Raw NoExPAN
Fit (fs=-0.34)
Intensity (a.u.)
1.25
1.00
0.80
0
180
360
Rotation angle of polarization (deg)

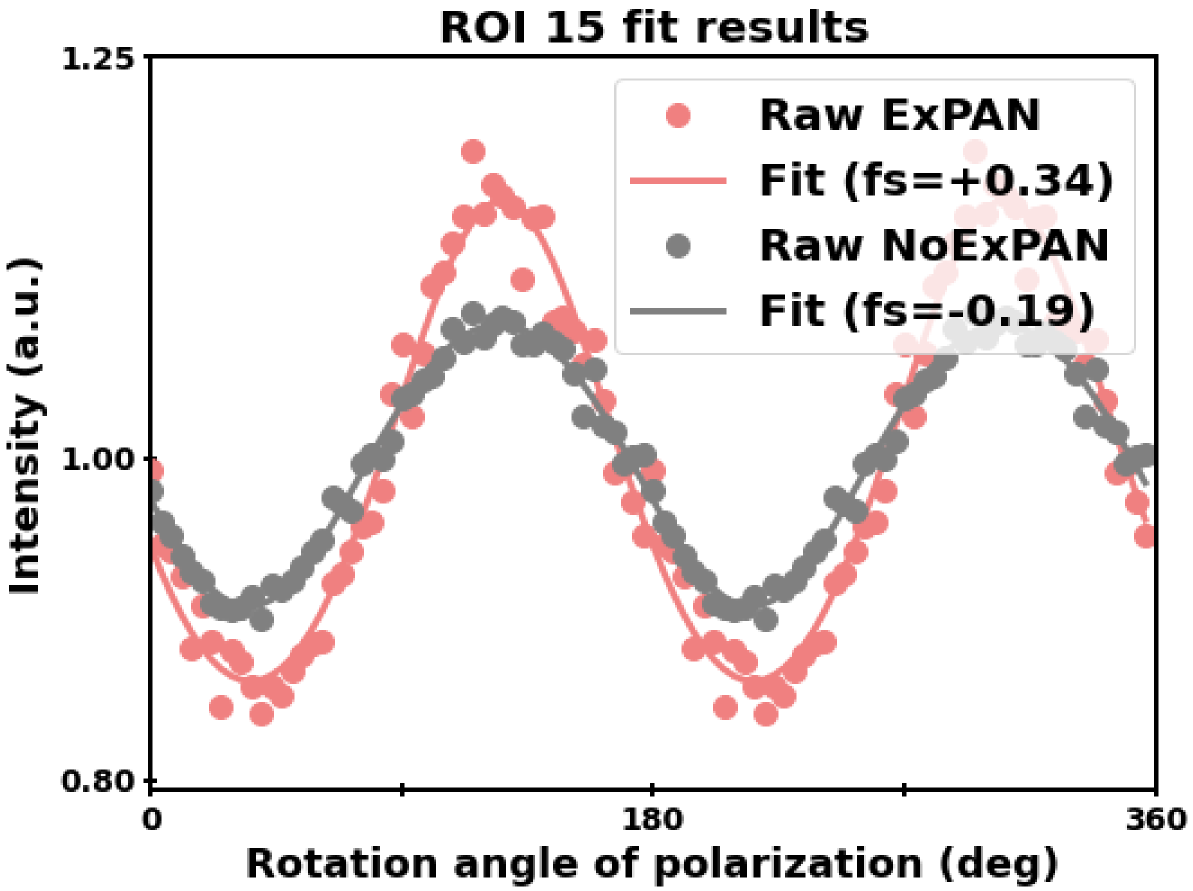
ROI 15 fit results
1.25
1.00
0.80
Intensity (a.u.)
0
180
360
Rotation angle of polarization (deg)
Raw ExPAN
Fit (fs=+0.34)
Raw NoExPAN
Fit (fs=-0.19)

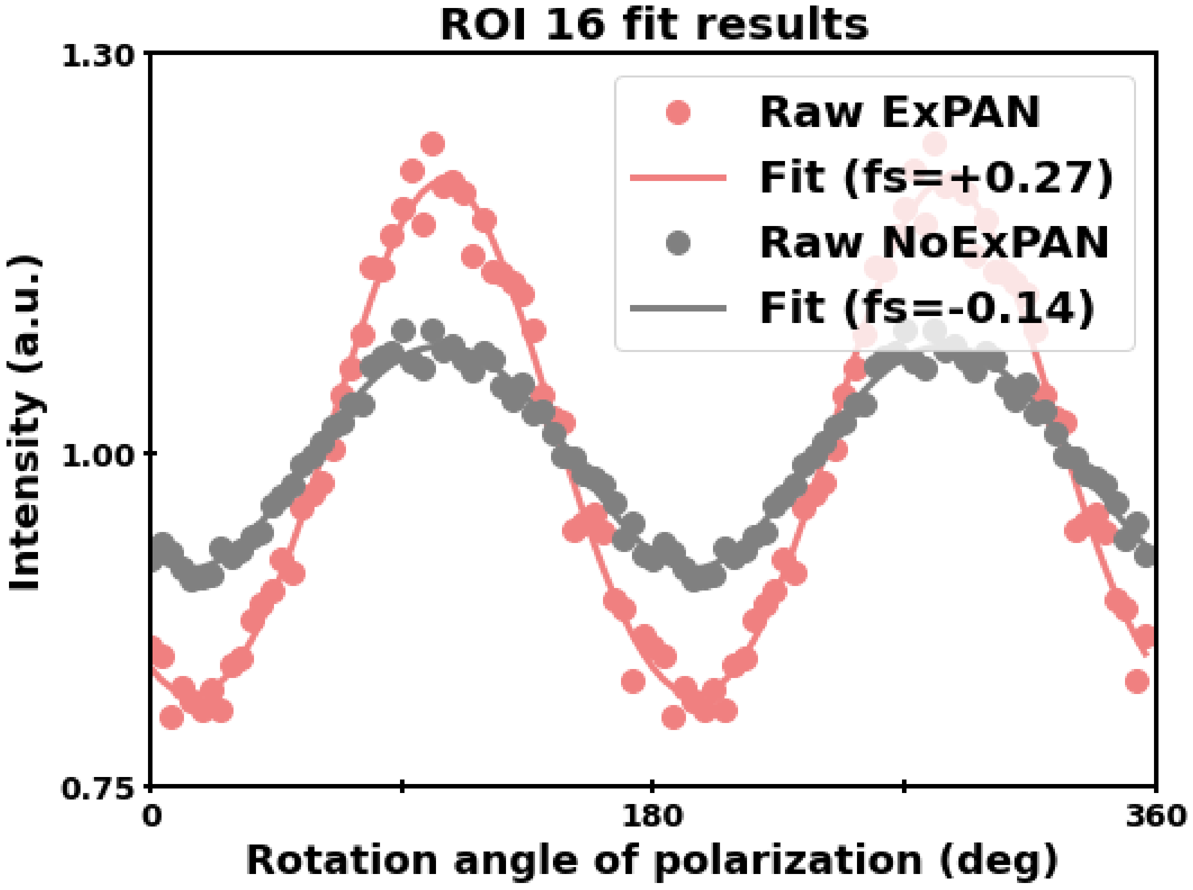
ROI 16 fit results
1.30
1.00
0.75
Intensity (a.u.)
0
180
360
Rotation angle of polarization (deg)
Raw ExPAN
Fit (fs=+0.27)
Raw NoExPAN
Fit (fs=-0.14)

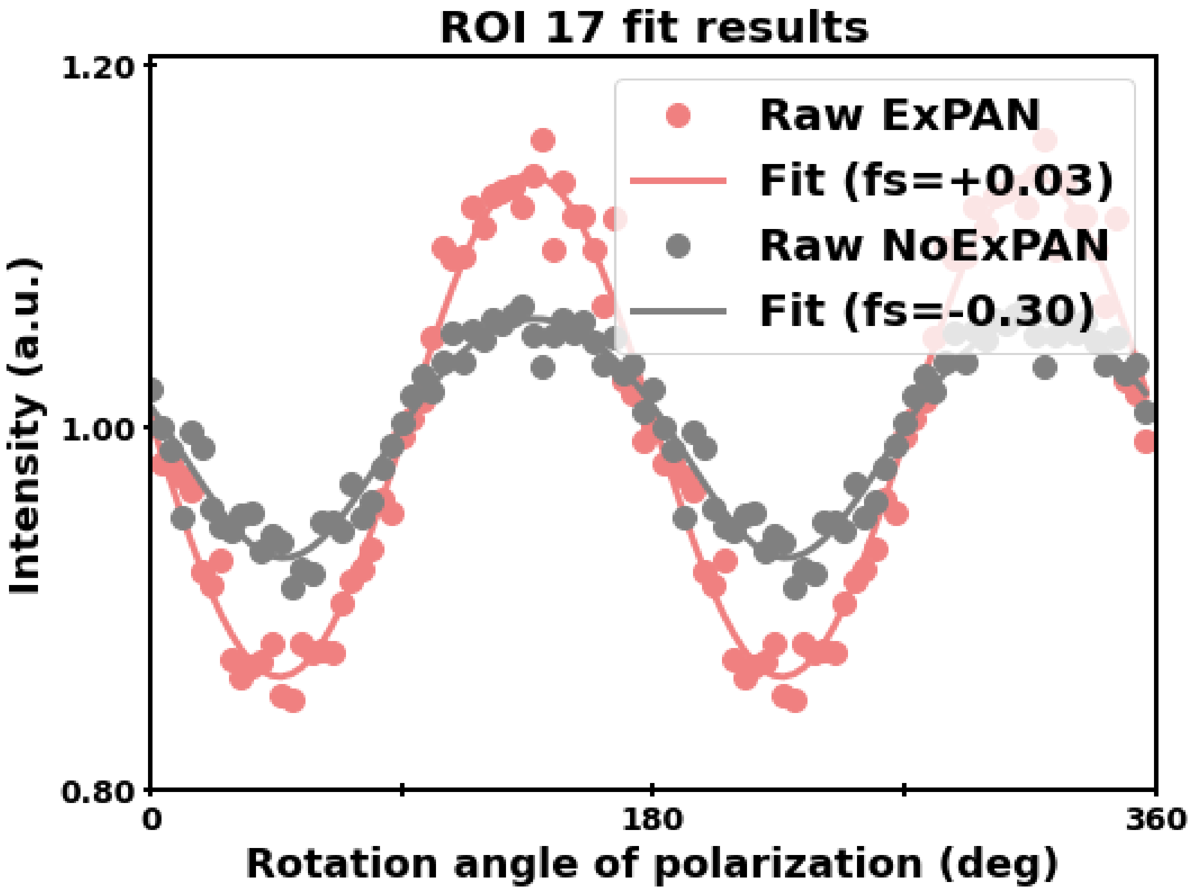
ROI 17 fit results
1.20
1.00
0.80
Intensity (a.u.)
0
180
360
Rotation angle of polarization (deg)
Raw ExPAN
Fit (fs=+0.03)
Raw NoExPAN
Fit (fs=-0.30)

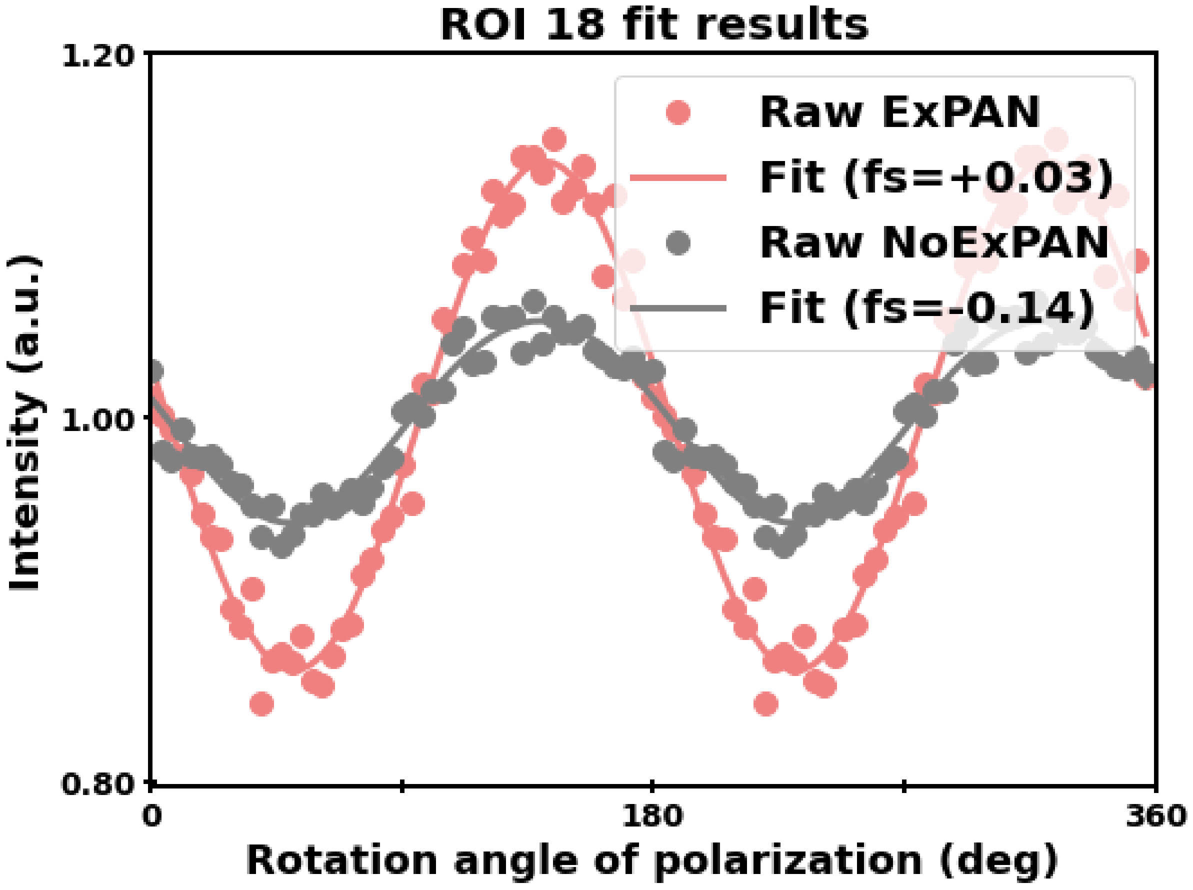
ROI 18 fit results
1.20
1.00
0.80
Intensity (a.u.)
0
180
360
Rotation angle of polarization (deg)
Raw ExPAN
Fit (fs=+0.03)
Raw NoExPAN
Fit (fs=-0.14)

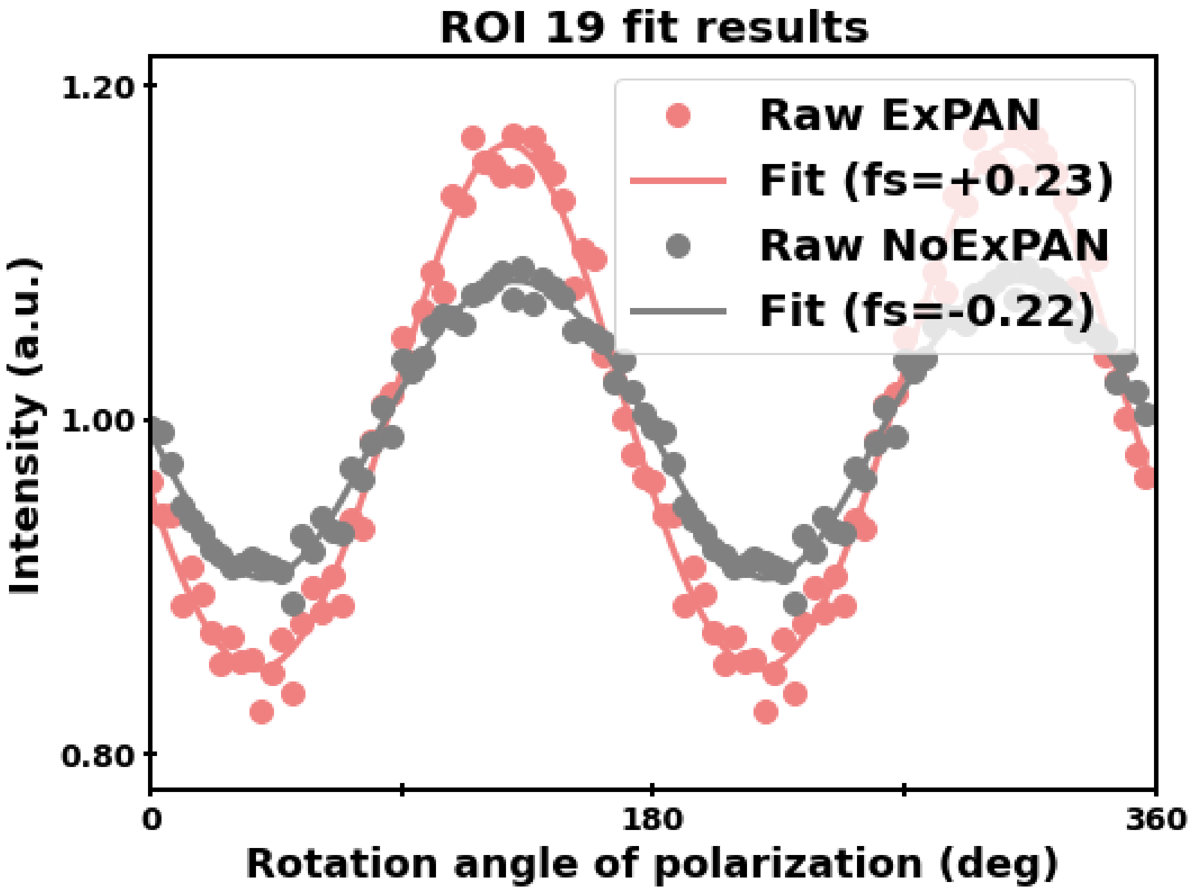
ROI 19 fit results
Raw ExPAN
Fit (fs=+0.23)
Raw NoExPAN
Fit (fs=-0.22)
Intensity (a.u.)
1.20
1.00
0.80
0
180
360
Rotation angle of polarization (deg)

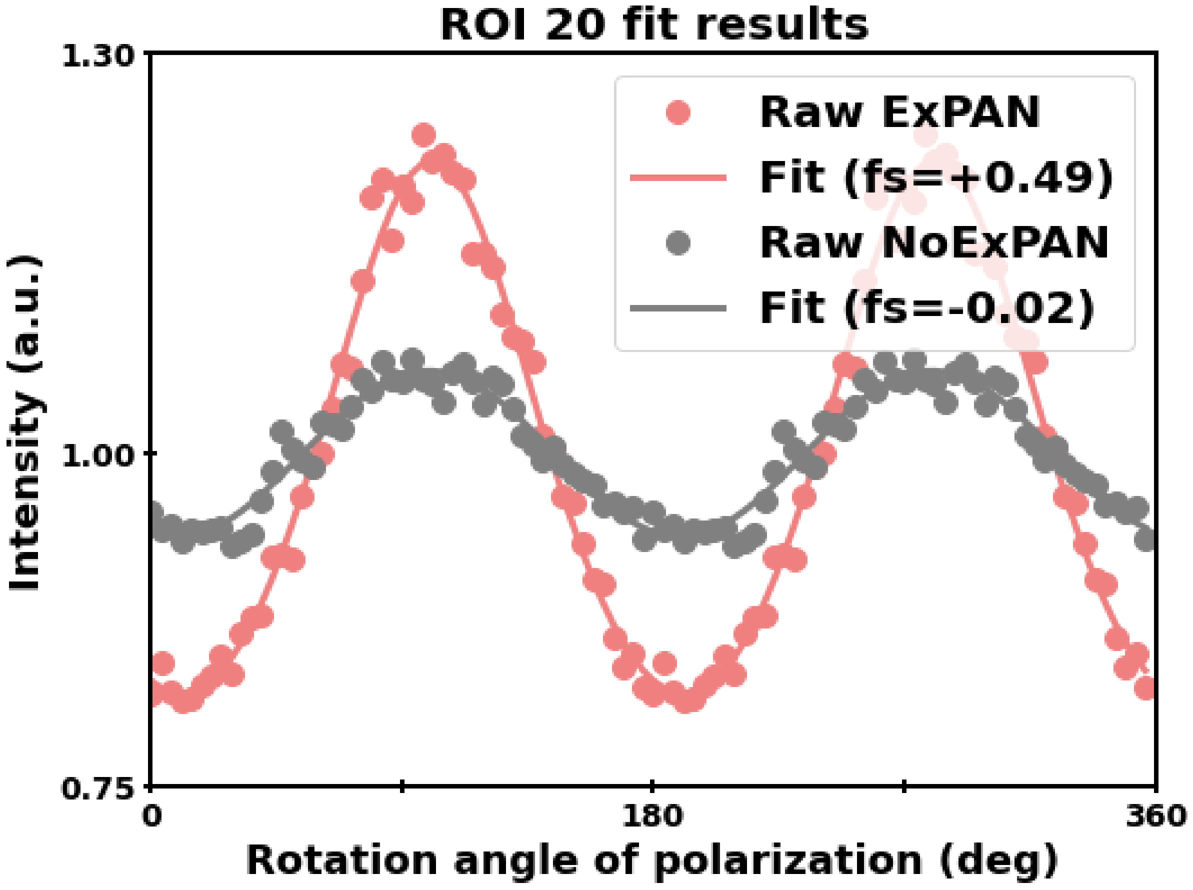
ROI 20 fit results
Raw ExPAN
Fit (fs=+0.49)
Raw NoExPAN
Fit (fs=-0.02)
Intensity (a.u.)
1.30
1.00
0.75
0
180
360
Rotation angle of polarization (deg)

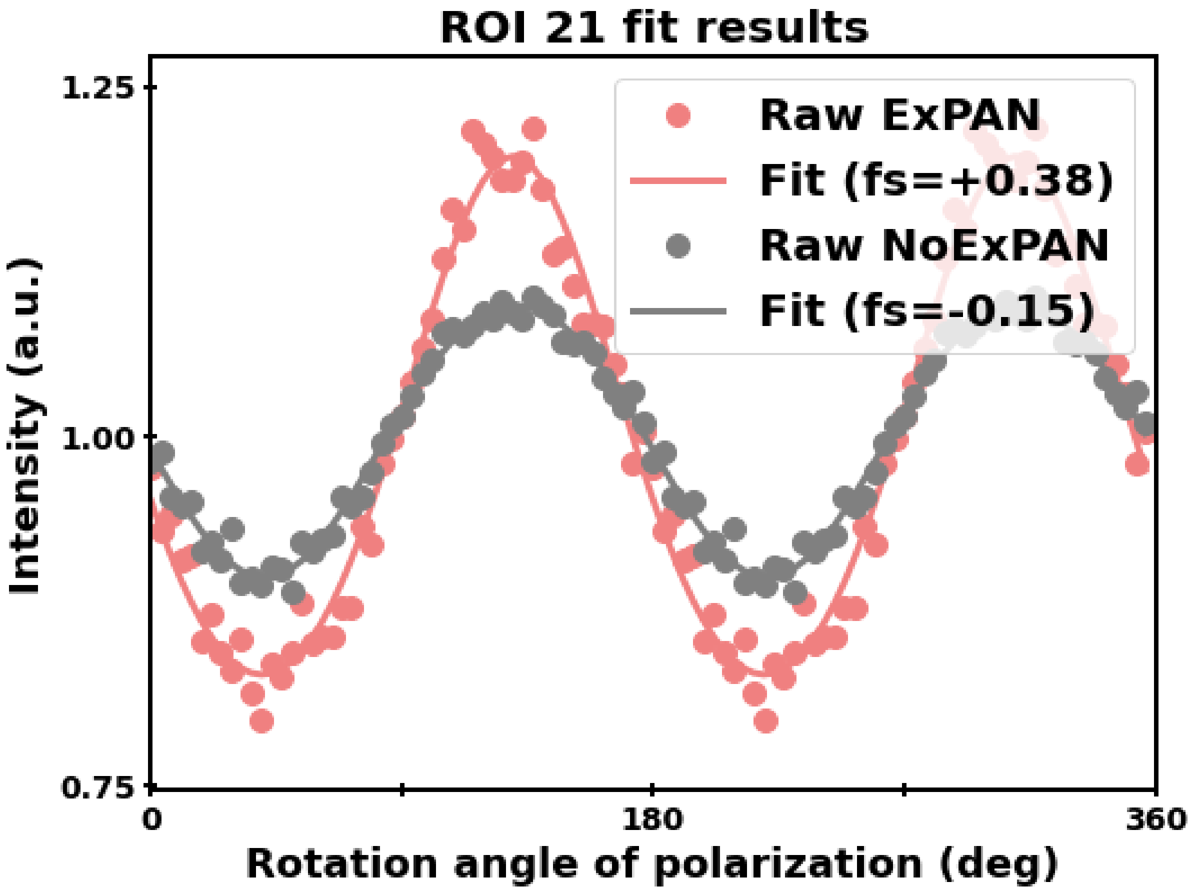
ROI 21 fit results
Raw ExPAN
Fit (fs=+0.38)
Raw NoExPAN
Fit (fs=-0.15)
Intensity (a.u.)
1.25
1.00
0.75
0
180
360
Rotation angle of polarization (deg)

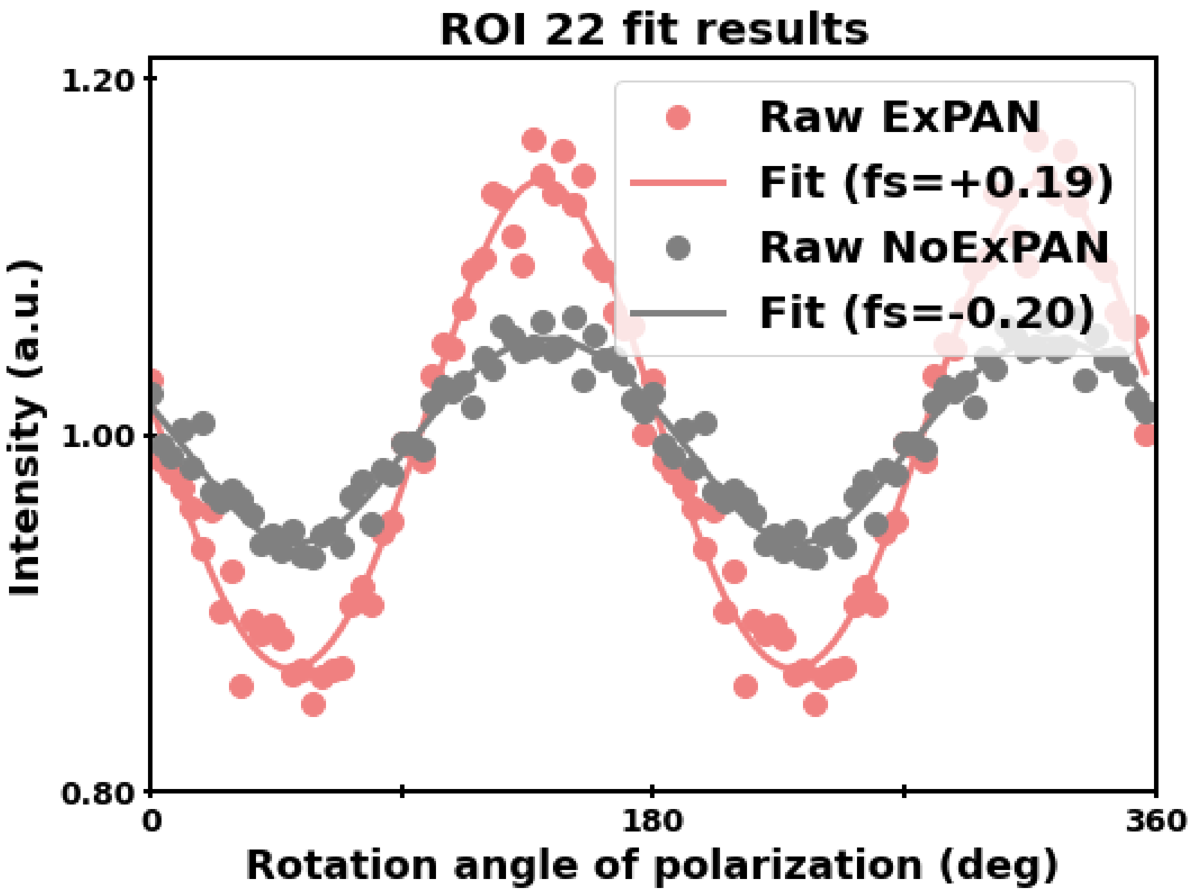
ROI 22 fit results
Raw ExPAN
Fit (fs=+0.19)
Raw NoExPAN
Fit (fs=-0.20)
Intensity (a.u.)
1.20
1.00
0.80
0
180
360
Rotation angle of polarization (deg)

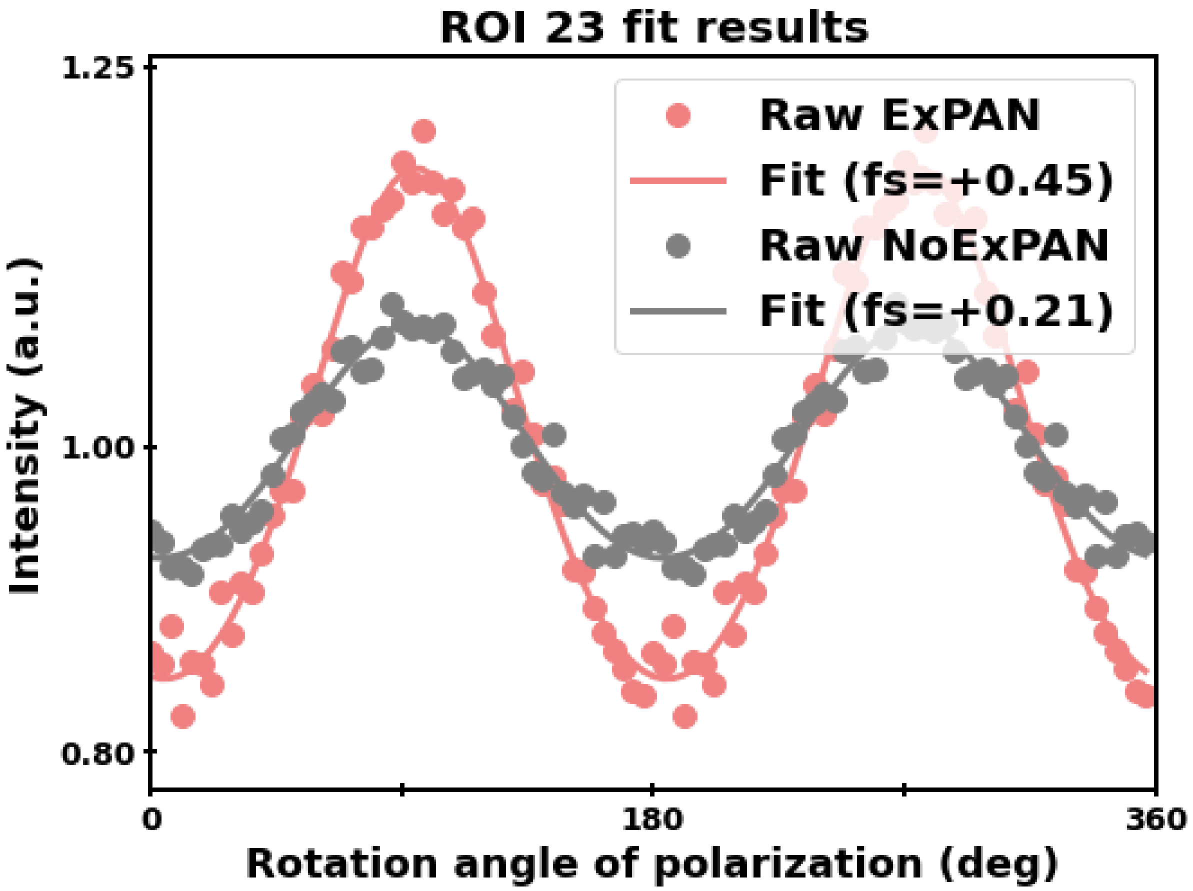
ROI 23 fit results
1.25
1.00
0.80
Intensity (a.u.)
0
180
360
Rotation angle of polarization (deg)
Raw ExPAN
Fit (fs=+0.45)
Raw NoExPAN
Fit (fs=+0.21)

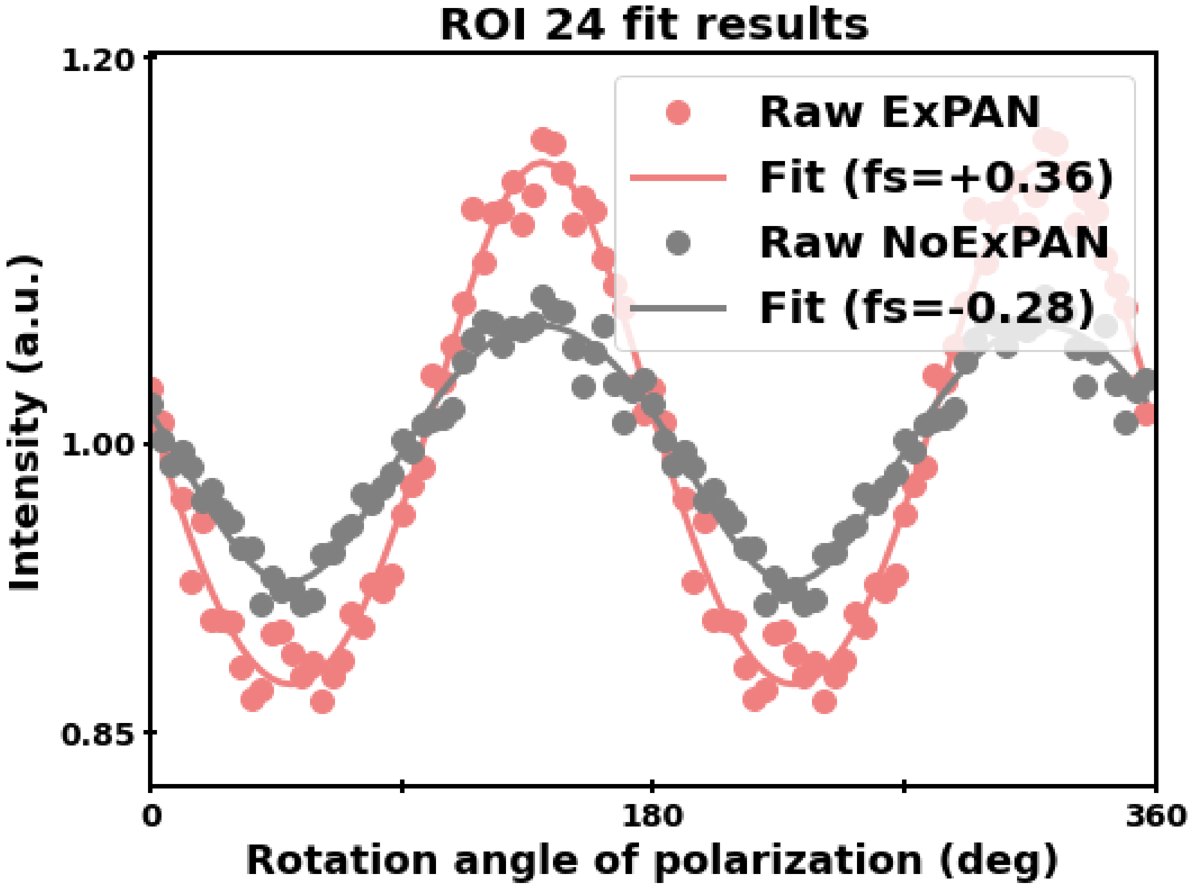
ROI 24 fit results
1.20
1.00
0.85
Intensity (a.u.)
0
180
360
Rotation angle of polarization (deg)
Raw ExPAN
Fit (fs=+0.36)
Raw NoExPAN
Fit (fs=-0.28)

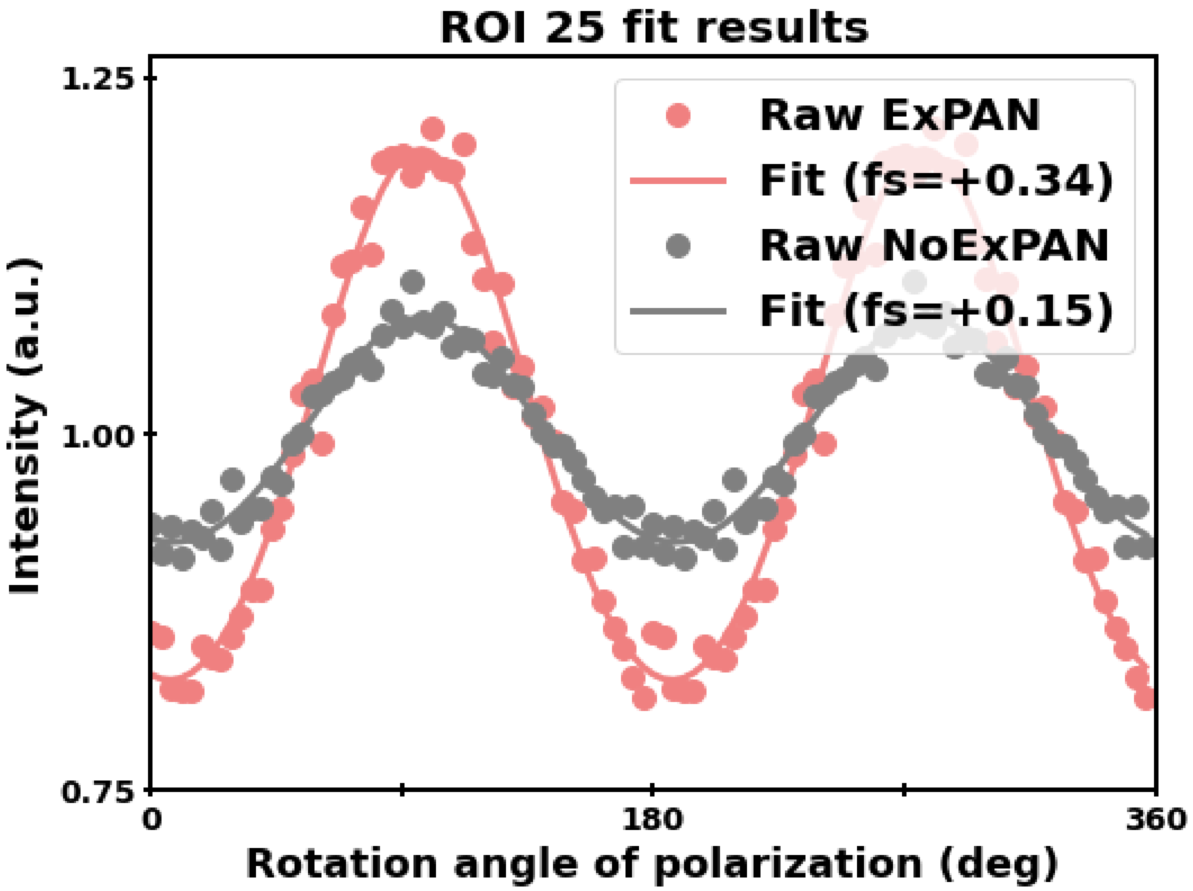
ROI 25 fit results
Raw ExPAN
Fit (fs=+0.34)
Raw NoExPAN
Fit (fs=+0.15)
Intensity (a.u.)
1.25
1.00
0.75
0
180
360
Rotation angle of polarization (deg)

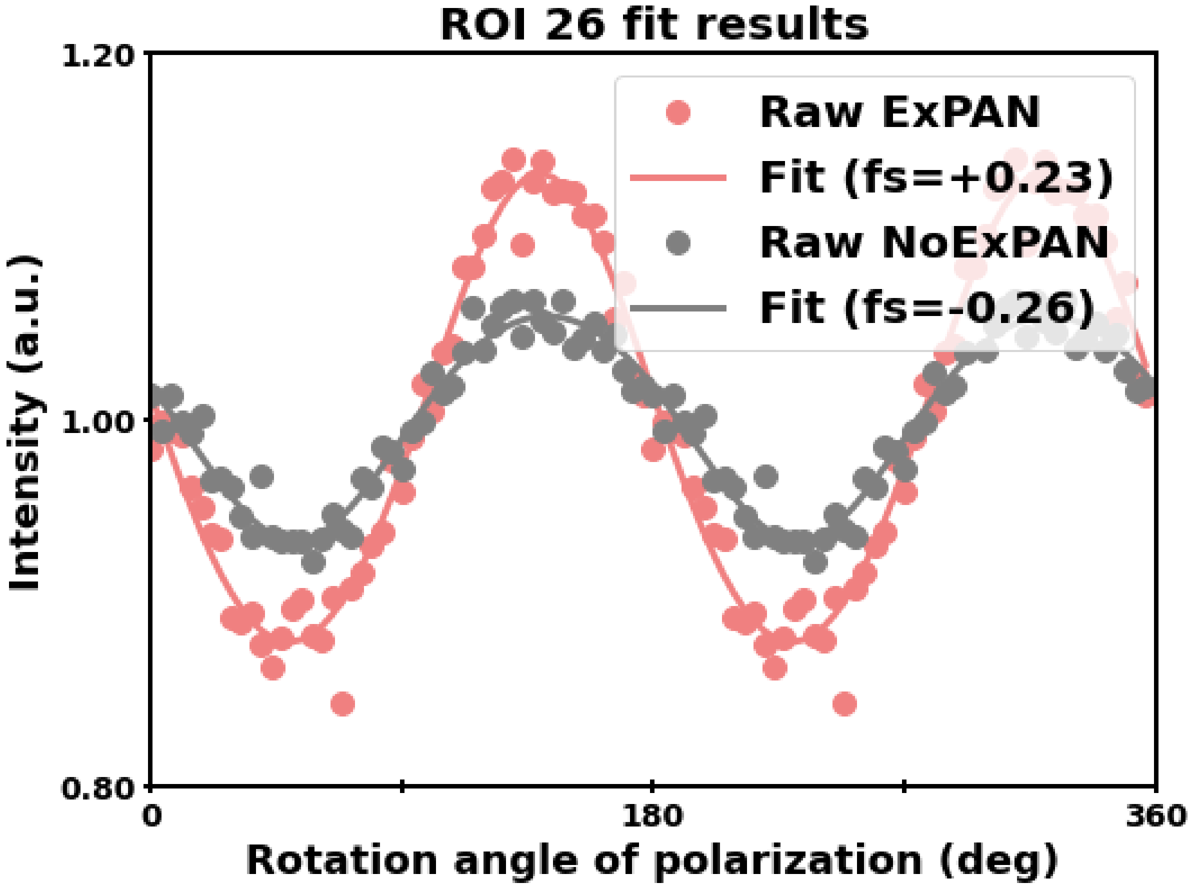
ROI 26 fit results
Raw ExPAN
Fit (fs=+0.23)
Raw NoExPAN
Fit (fs=-0.26)
Intensity (a.u.)
1.20
1.00
0.80
0
180
360
Rotation angle of polarization (deg)

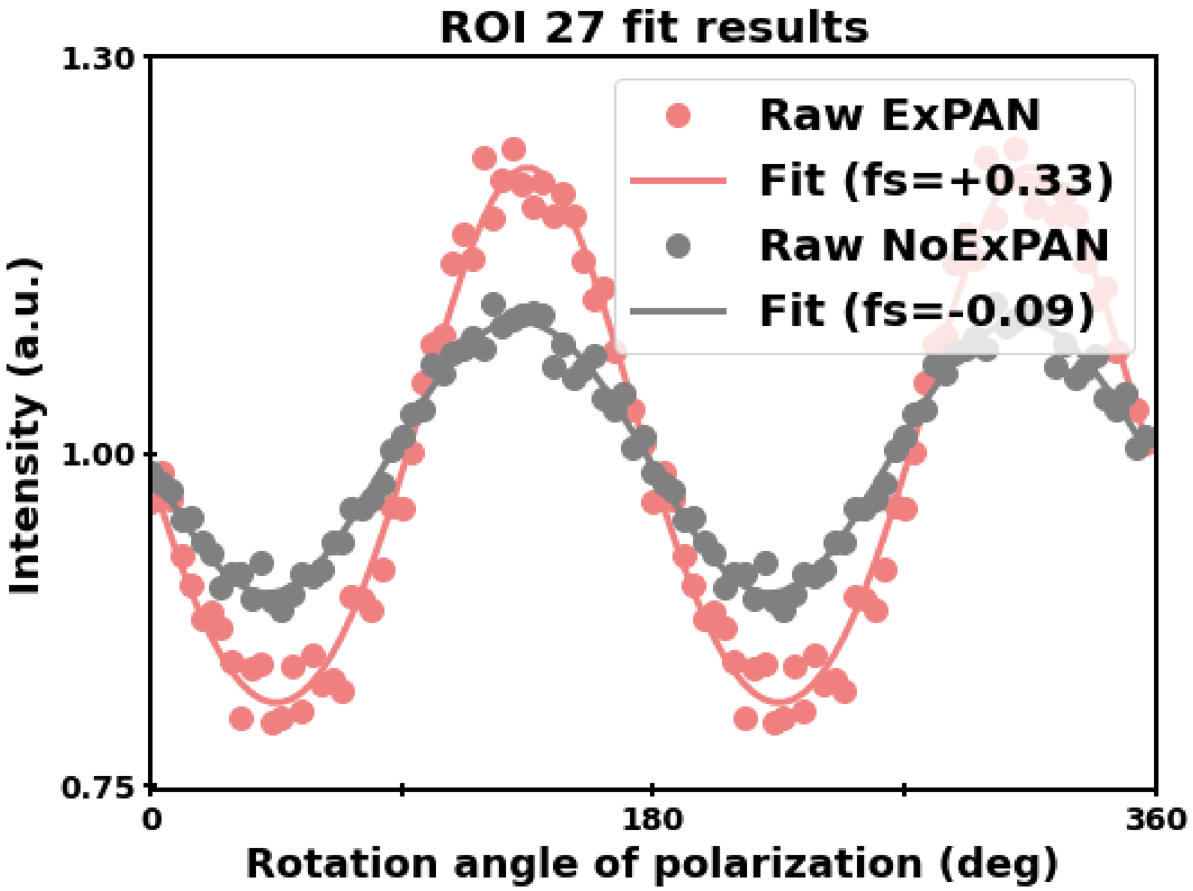
ROI 27 fit results
1.30
1.00
0.75
Intensity (a.u.)
0
180
360
Rotation angle of polarization (deg)
Raw ExPAN
Fit (fs=+0.33)
Raw NoExPAN
Fit (fs=-0.09)

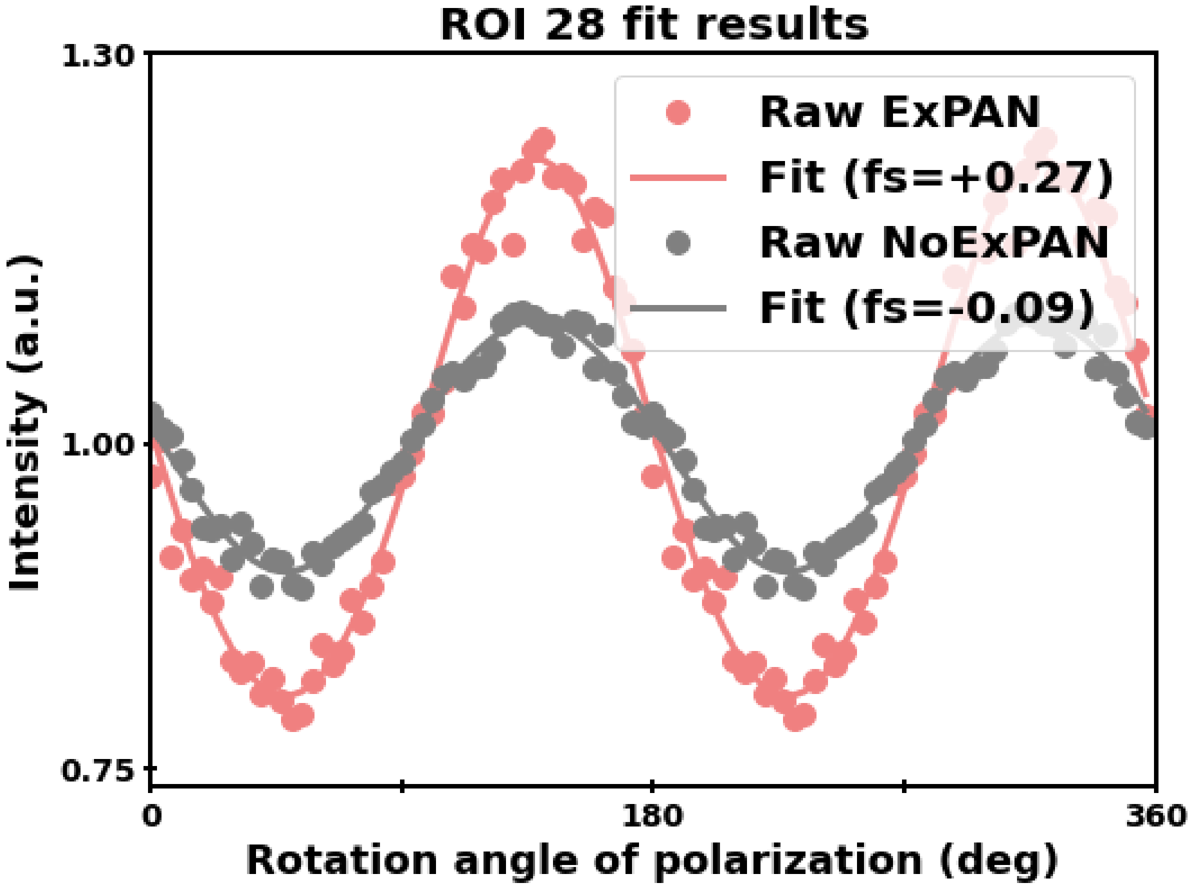
ROI 28 fit results
1.30
1.00
0.75
Intensity (a.u.)
0
180
360
Rotation angle of polarization (deg)
Raw ExPAN
Fit (fs=+0.27)
Raw NoExPAN
Fit (fs=-0.09)

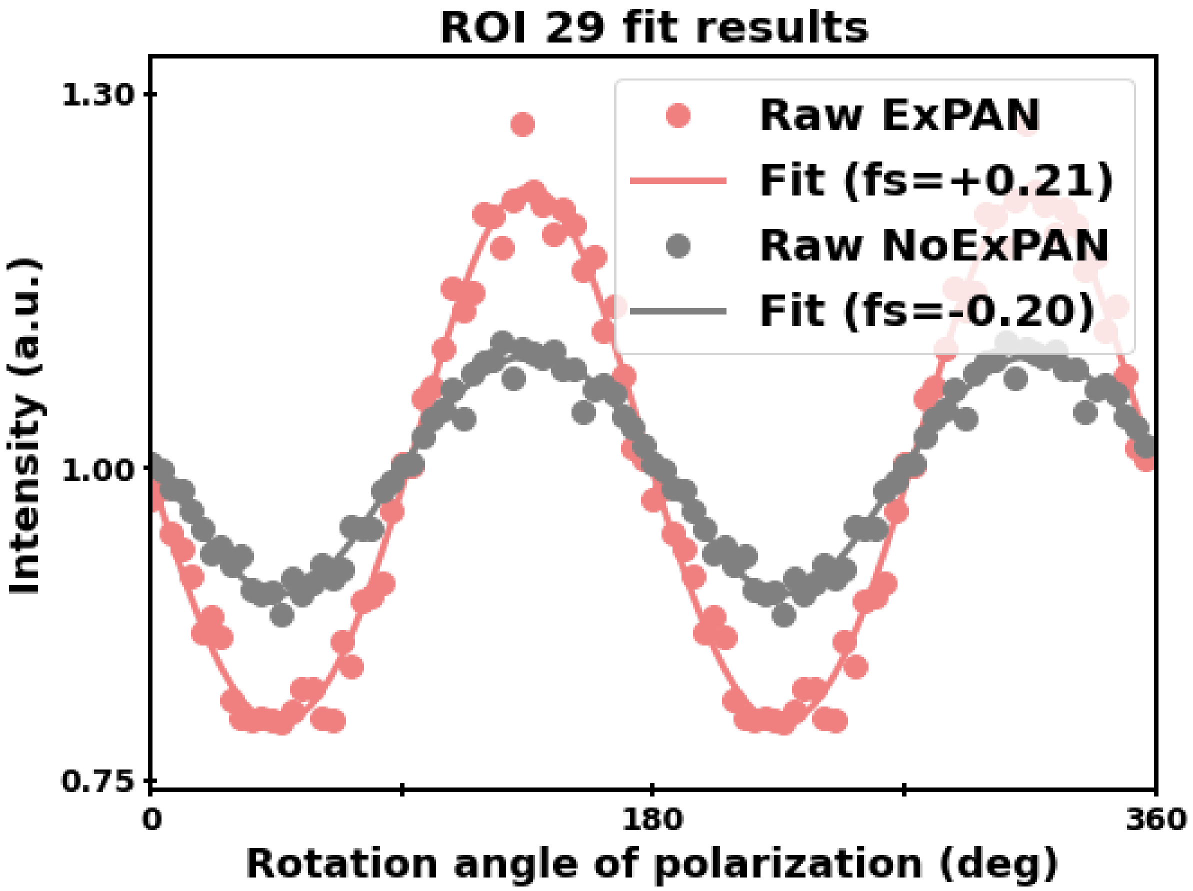
ROI 29 fit results
Raw ExPAN
Fit (fs=+0.21)
Raw NoExPAN
Fit (fs=-0.20)
Intensity (a.u.)
1.30
1.00
0.75
0
180
360
Rotation angle of polarization (deg)

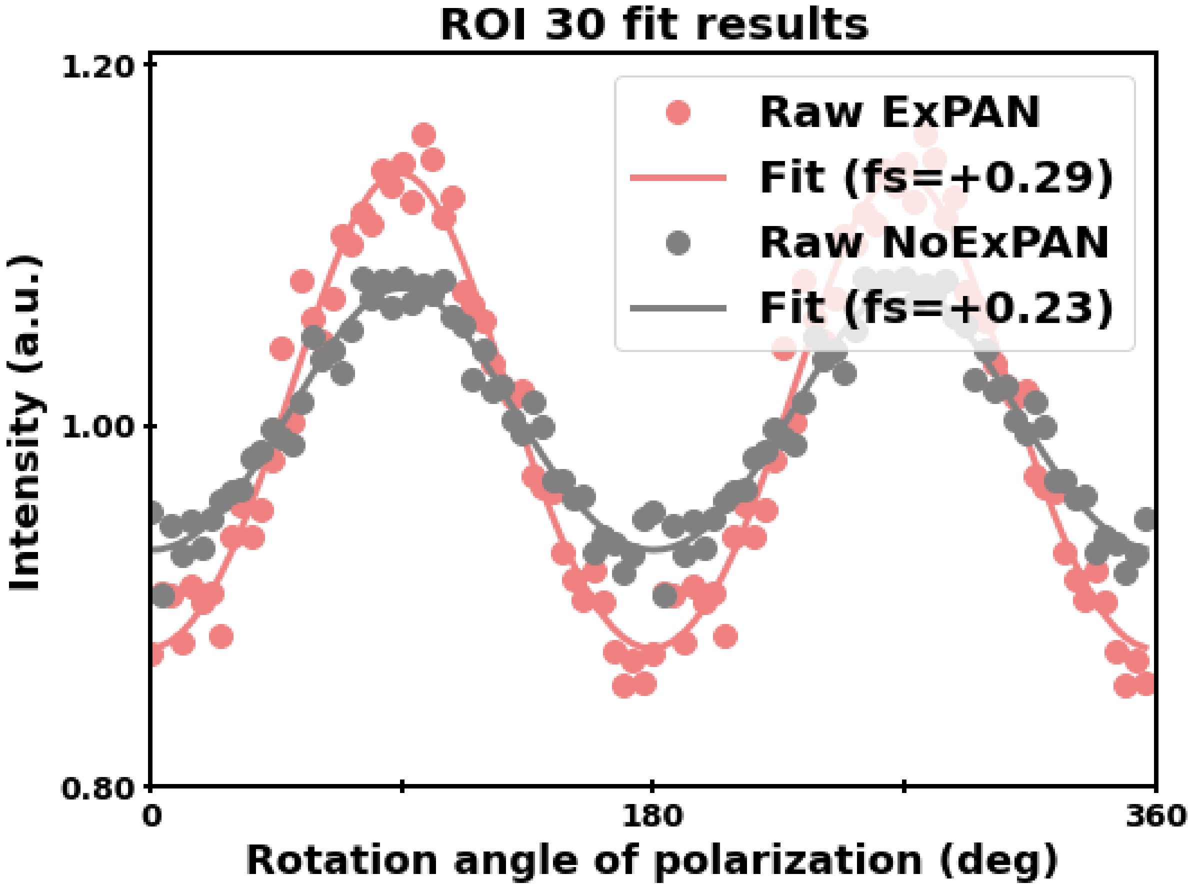
ROI 30 fit results
Raw ExPAN
Fit (fs=+0.29)
Raw NoExPAN
Fit (fs=+0.23)
Intensity (a.u.)
1.20
1.00
0.80
0
180
360
Rotation angle of polarization (deg)

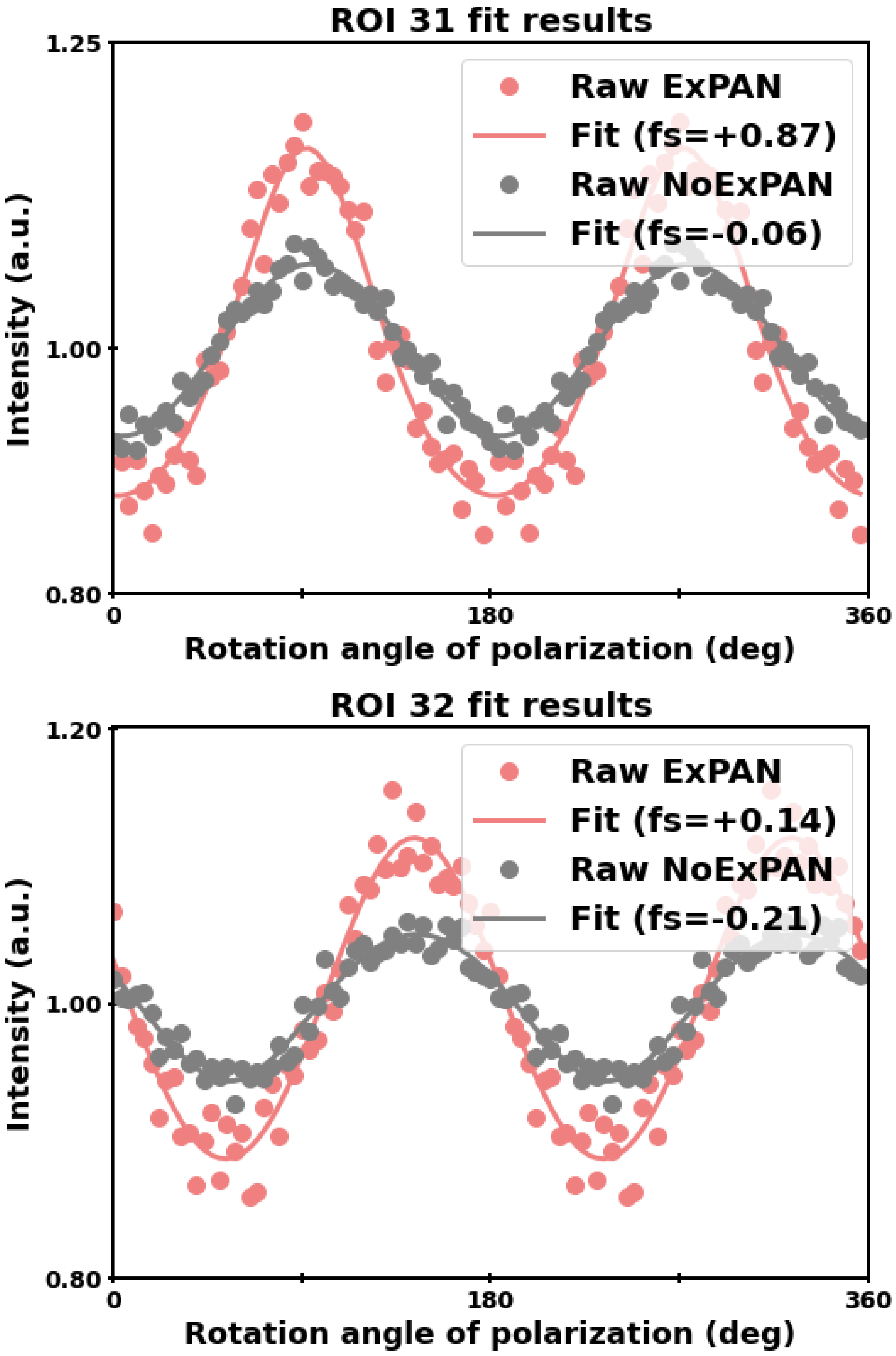
ROI 31 fit results
Raw ExPAN
Fit (fs=+0.87)
Raw NoExPAN
Fit (fs=-0.06)
Intensity (a.u.)
1.25
1.00
0.80
0
180
360
Rotation angle of polarization (deg)
ROI 32 fit results
Raw ExPAN
Fit (fs=+0.14)
Raw NoExPAN
Fit (fs=-0.21)
Intensity (a.u.)
1.20
1.00
0.80
0
180
360
Rotation angle of polarization (deg)

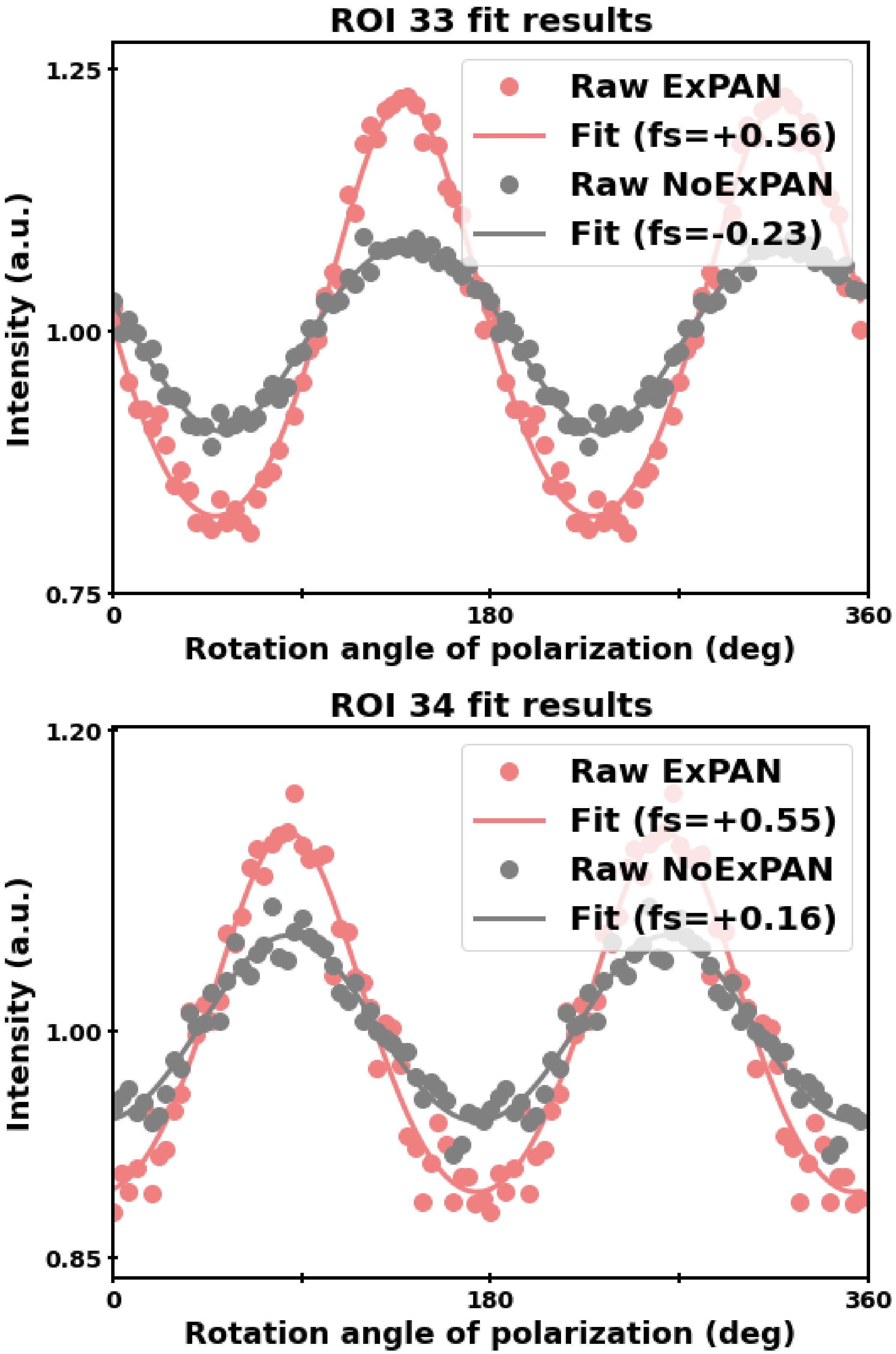
ROI 33 fit results
Raw ExPAN
Fit (fs=+0.56)
Raw NoExPAN
Fit (fs=-0.23)
Intensity (a.u.)
1.25
1.00
0.75
0
180
360
Rotation angle of polarization (deg)
ROI 34 fit results
Raw ExPAN
Fit (fs=+0.55)
Raw NoExPAN
Fit (fs=+0.16)
Intensity (a.u.)
1.20
1.00
0.85
0
180
360
Rotation angle of polarization (deg)

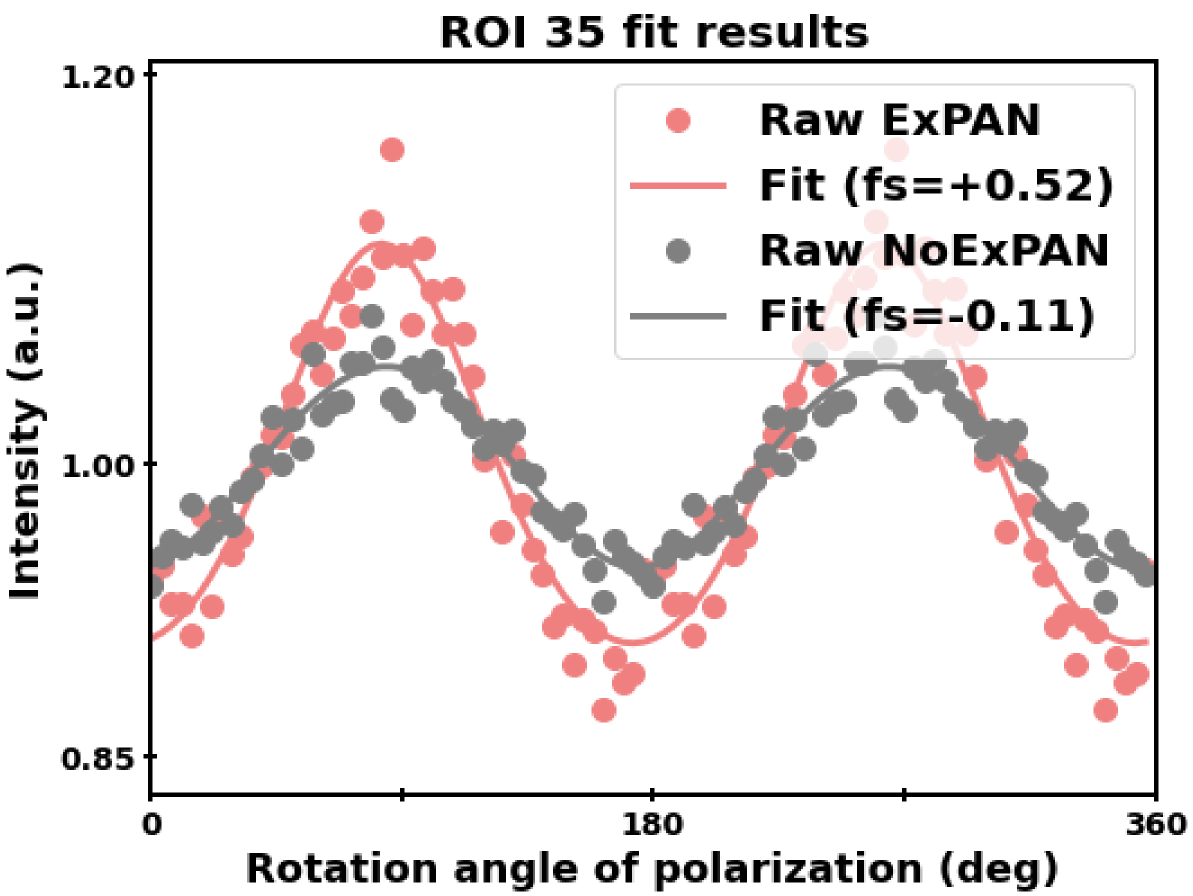
ROI 35 fit results
1.20
1.00
0.85
Intensity (a.u.)
0
180
360
Rotation angle of polarization (deg)
Raw ExPAN
Fit (fs=+0.52)
Raw NoExPAN
Fit (fs=-0.11)

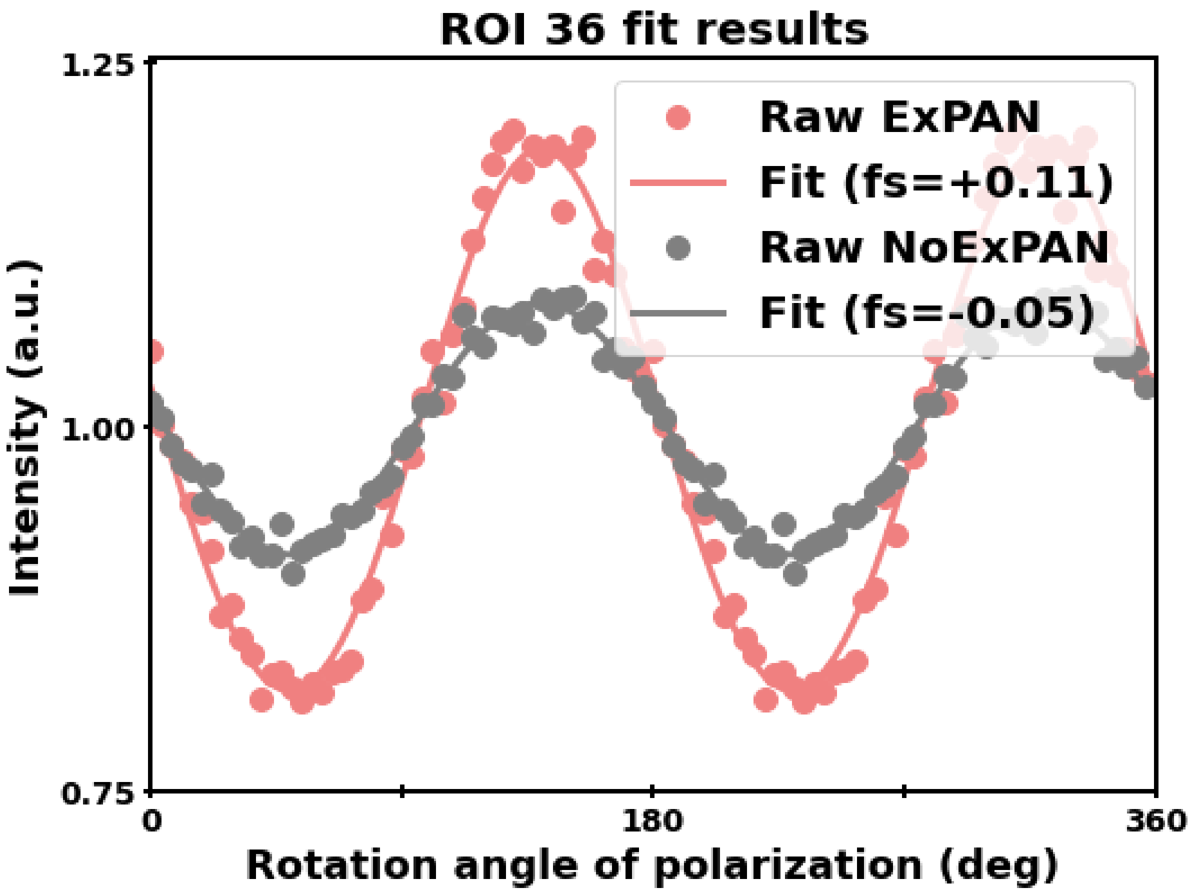
ROI 36 fit results
1.25
1.00
0.75
Intensity (a.u.)
0
180
360
Rotation angle of polarization (deg)
Raw ExPAN
Fit (fs=+0.11)
Raw NoExPAN
Fit (fs=-0.05)

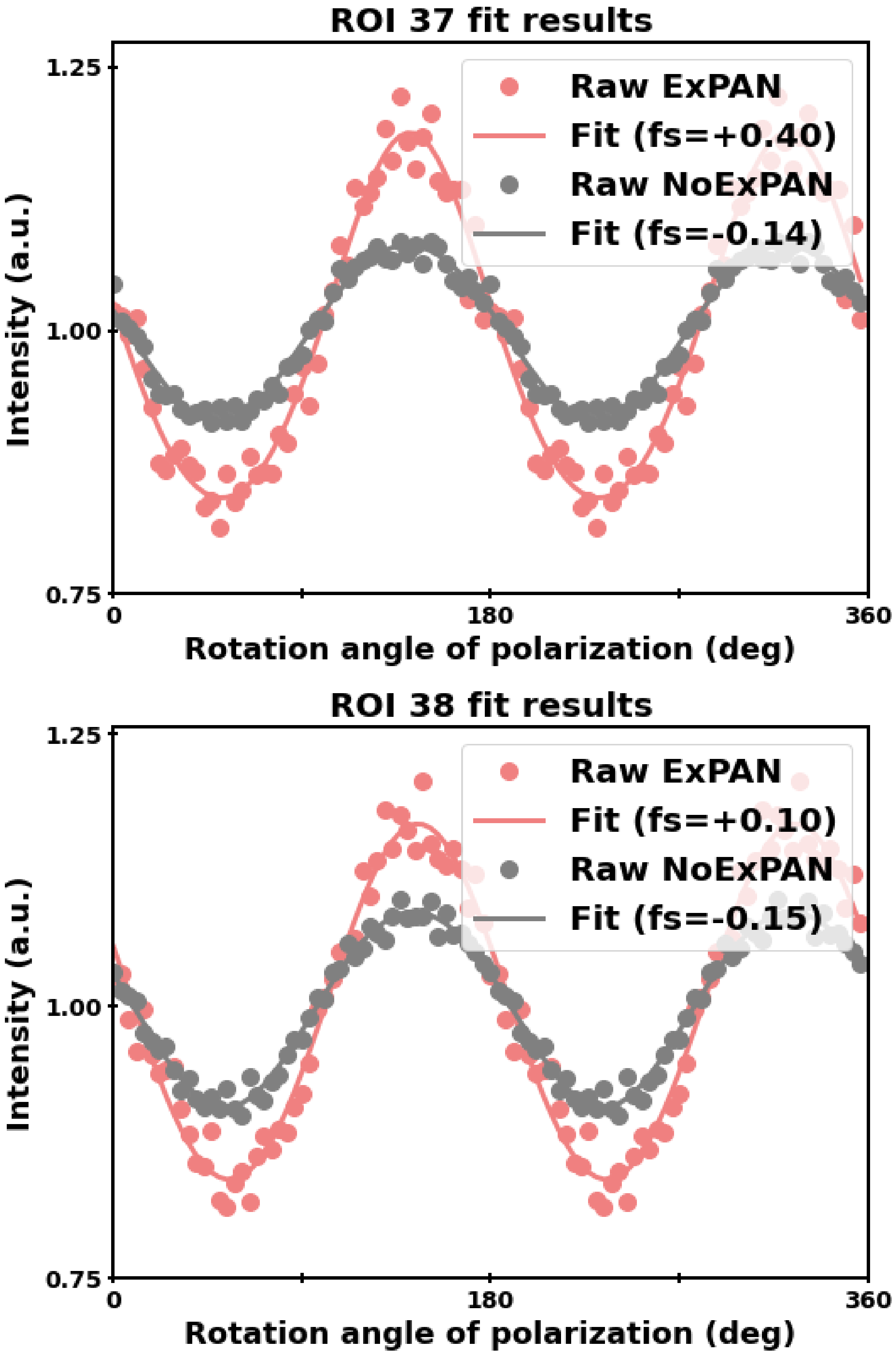

ROI 37 fit results
Raw ExPAN
Fit (fs=+0.40)
Raw NoExPAN
Fit (fs=-0.14)
1.25
1.00
0.75
Intensity (a.u.)
0
180
360
Rotation angle of polarization (deg)
ROI 38 fit results
Raw ExPAN
Fit (fs=+0.10)
Raw NoExPAN
Fit (fs=-0.15)
1.25
1.00
0.75
Intensity (a.u.)
0
180
360
Rotation angle of polarization (deg)

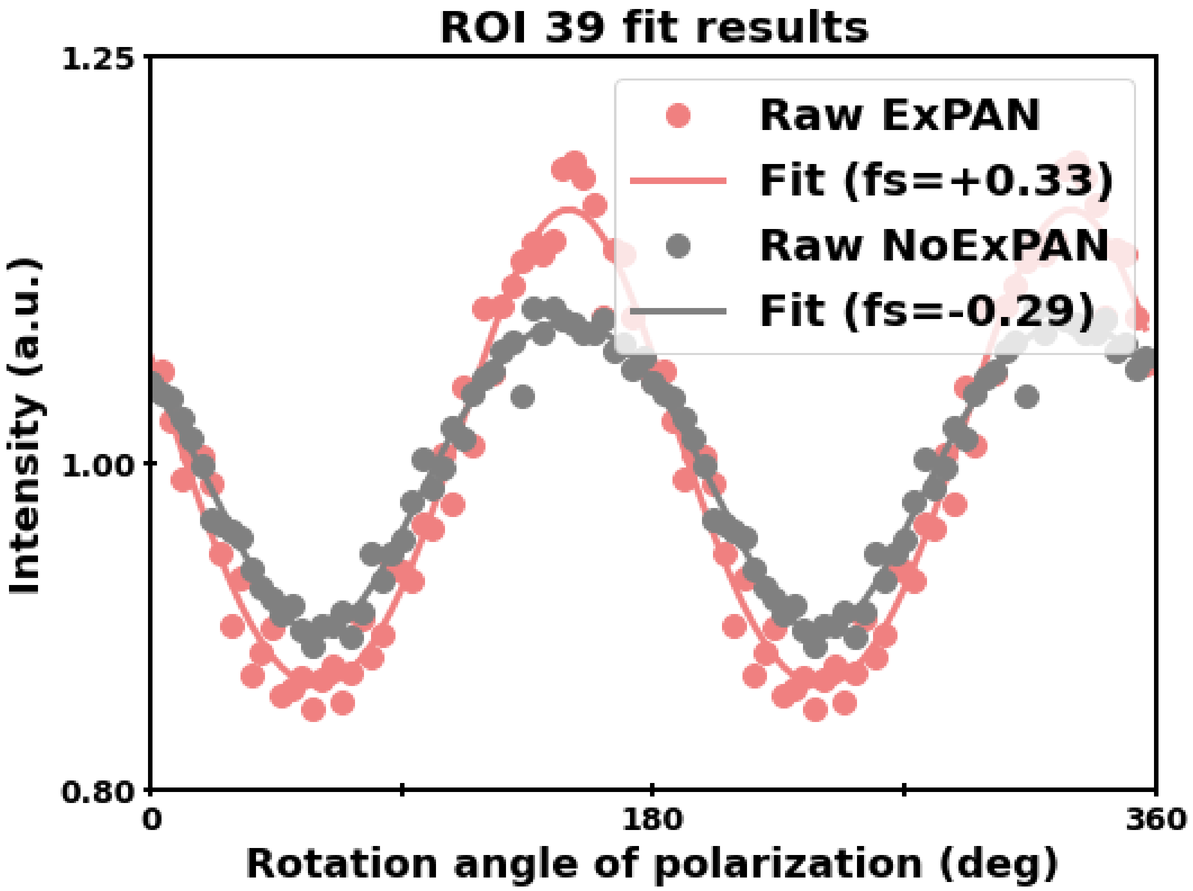

ROI 39 fit results
1.25
1.00
0.80
Intensity (a.u.)
0
180
360
Rotation angle of polarization (deg)
Raw ExPAN
Fit (fs=+0.33)
Raw NoExPAN
Fit (fs=-0.29)

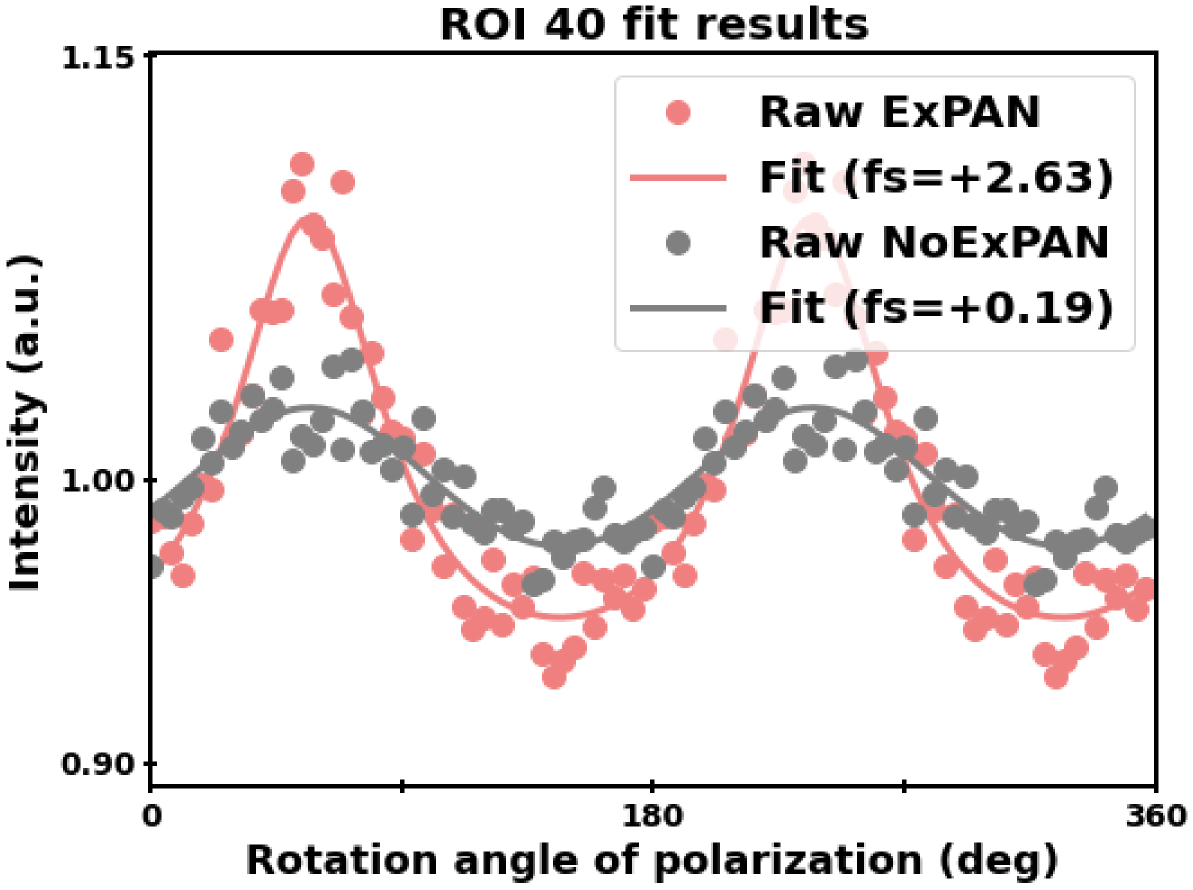

ROI 40 fit results
1.15
1.00
0.90
Intensity (a.u.)
0
180
360
Rotation angle of polarization (deg)
Raw ExPAN
Fit (fs=+2.63)
Raw NoExPAN
Fit (fs=+0.19)

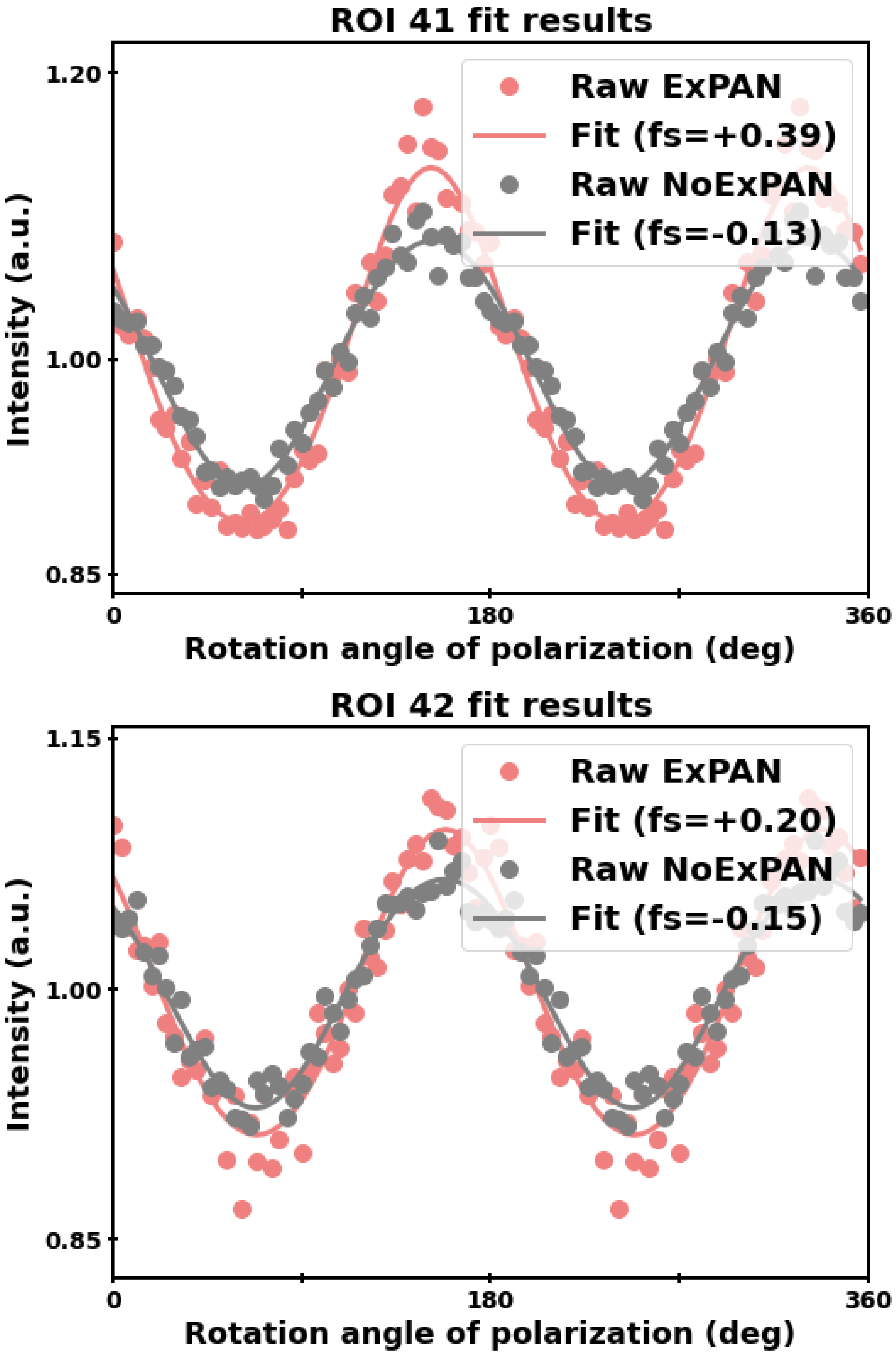
ROI 41 fit results
1.20
1.00
0.85
Intensity (a.u.)
0
180
360
Rotation angle of polarization (deg)
Raw ExPAN
Fit (fs=+0.39)
Raw NoExPAN
Fit (fs=-0.13)
ROI 42 fit results
1.15
1.00
0.85
Intensity (a.u.)
0
180
360
Rotation angle of polarization (deg)
Raw ExPAN
Fit (fs=+0.20)
Raw NoExPAN
Fit (fs=-0.15)

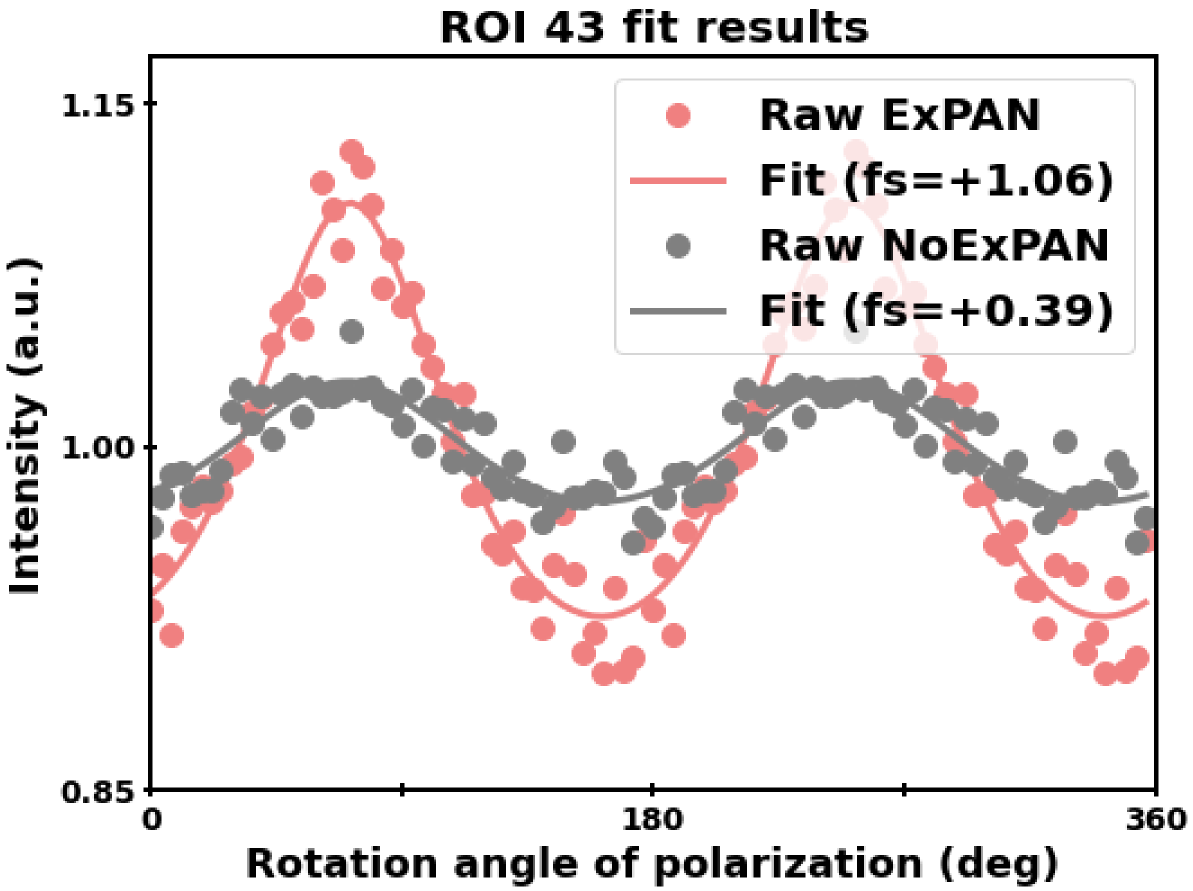
ROI 43 fit results
Intensity (a.u.)
1.15
1.00
0.85
0
180
360
Rotation angle of polarization (deg)
Raw ExPAN
Fit (fs=+1.06)
Raw NoExPAN
Fit (fs=+0.39)

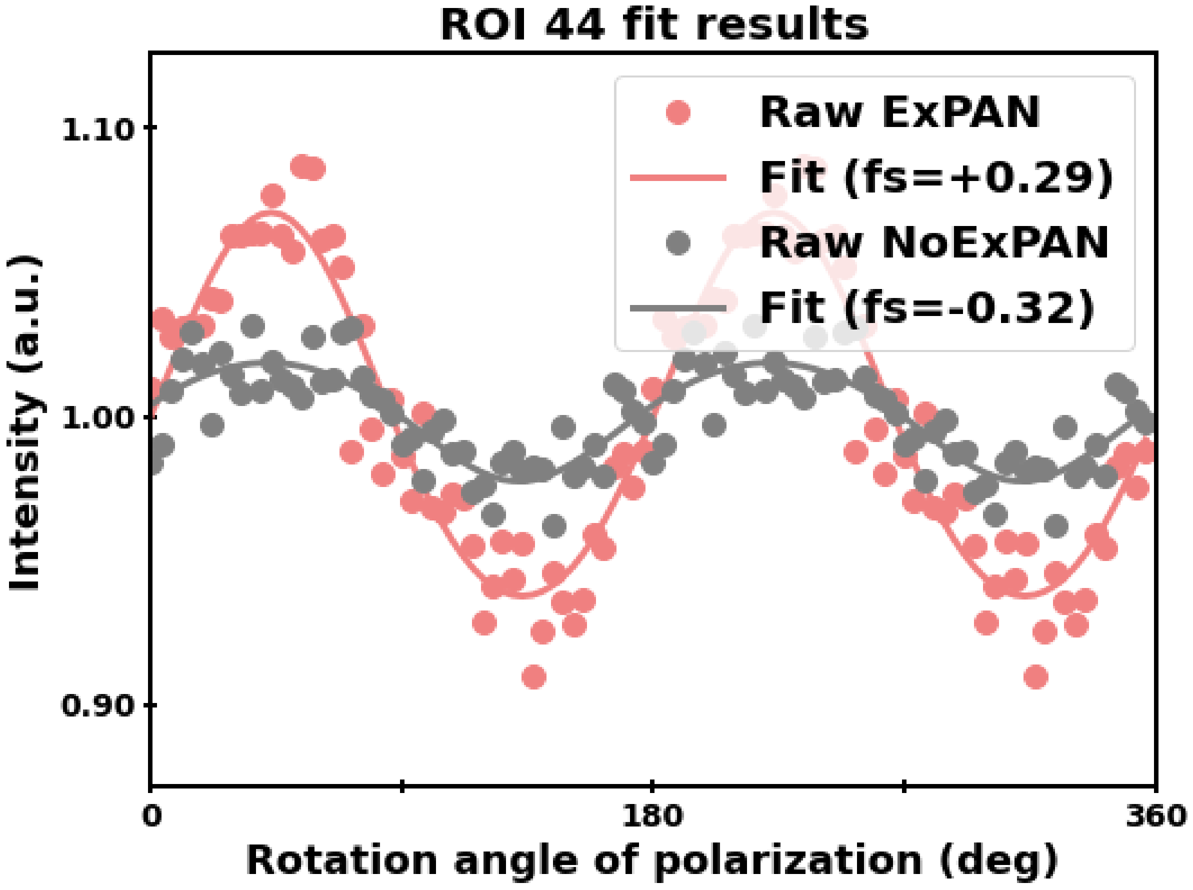
ROI 44 fit results
Intensity (a.u.)
1.10
1.00
0.90
0
180
360
Rotation angle of polarization (deg)
Raw ExPAN
Fit (fs=+0.29)
Raw NoExPAN
Fit (fs=-0.32)

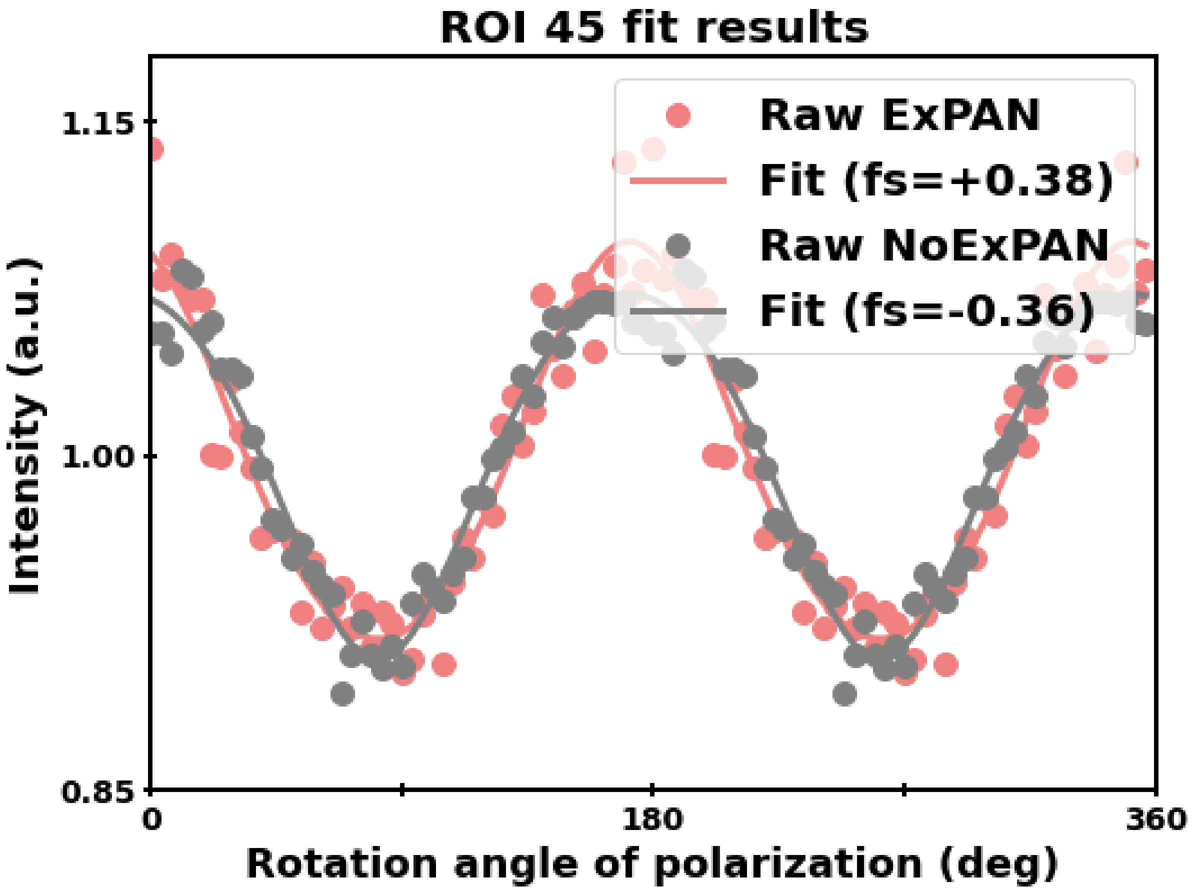

ROI 45 fit results
Raw ExPAN
Fit (fs=+0.38)
Raw NoExPAN
Fit (fs=-0.36)
Intensity (a.u.)
1.15
1.00
0.85
0
180
360
Rotation angle of polarization (deg)

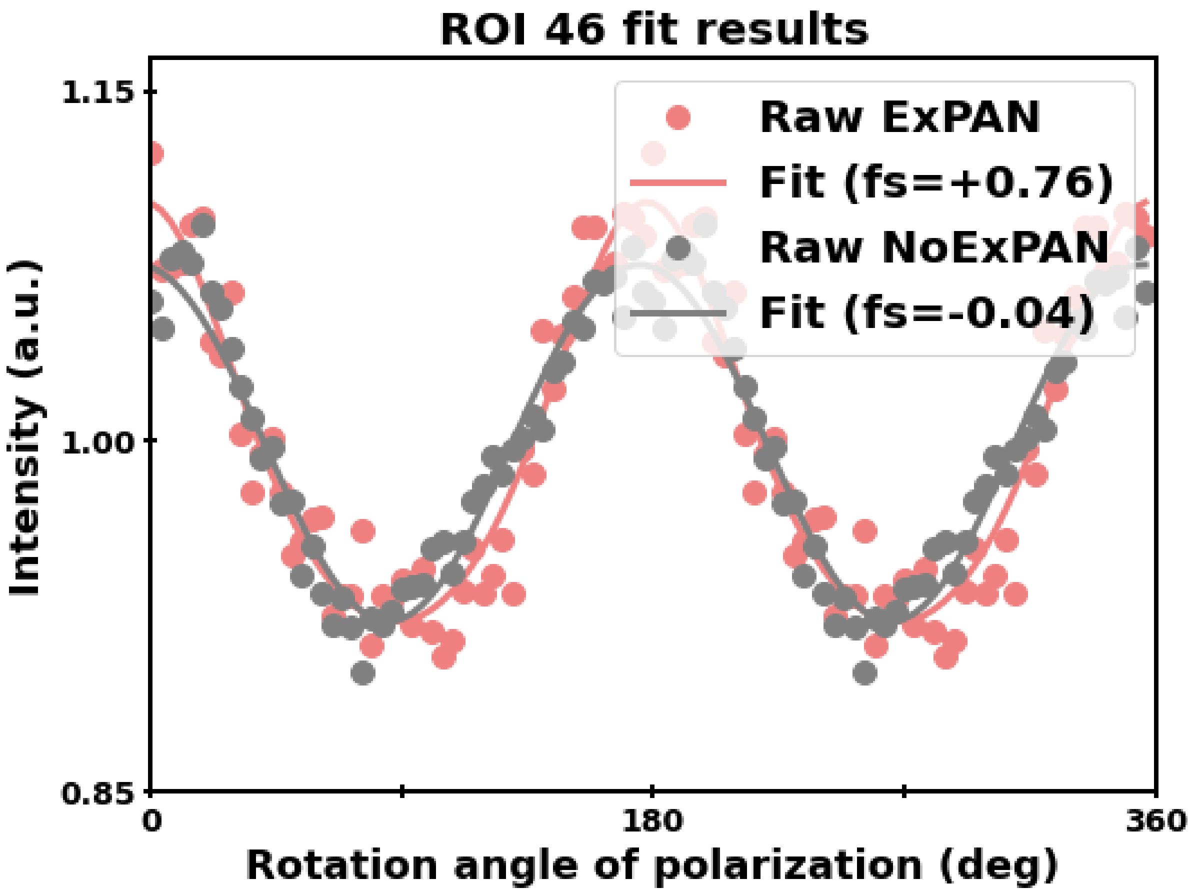

ROI 46 fit results
Raw ExPAN
Fit (fs=+0.76)
Raw NoExPAN
Fit (fs=-0.04)
Intensity (a.u.)
1.15
1.00
0.85
0
180
360
Rotation angle of polarization (deg)

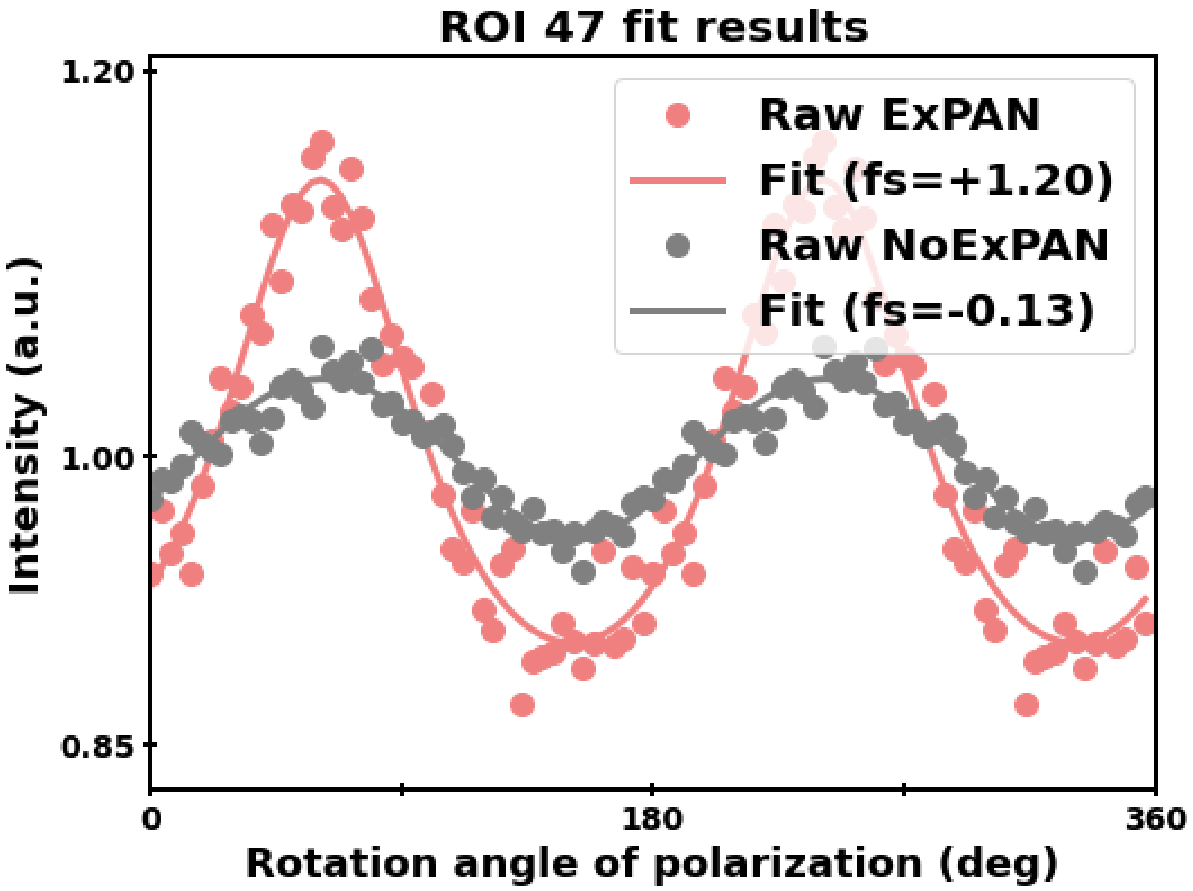
ROI 47 fit results
Raw ExPAN
Fit (fs=+1.20)
Raw NoExPAN
Fit (fs=-0.13)
1.20
1.00
0.85
Intensity (a.u.)
0
180
360
Rotation angle of polarization (deg)

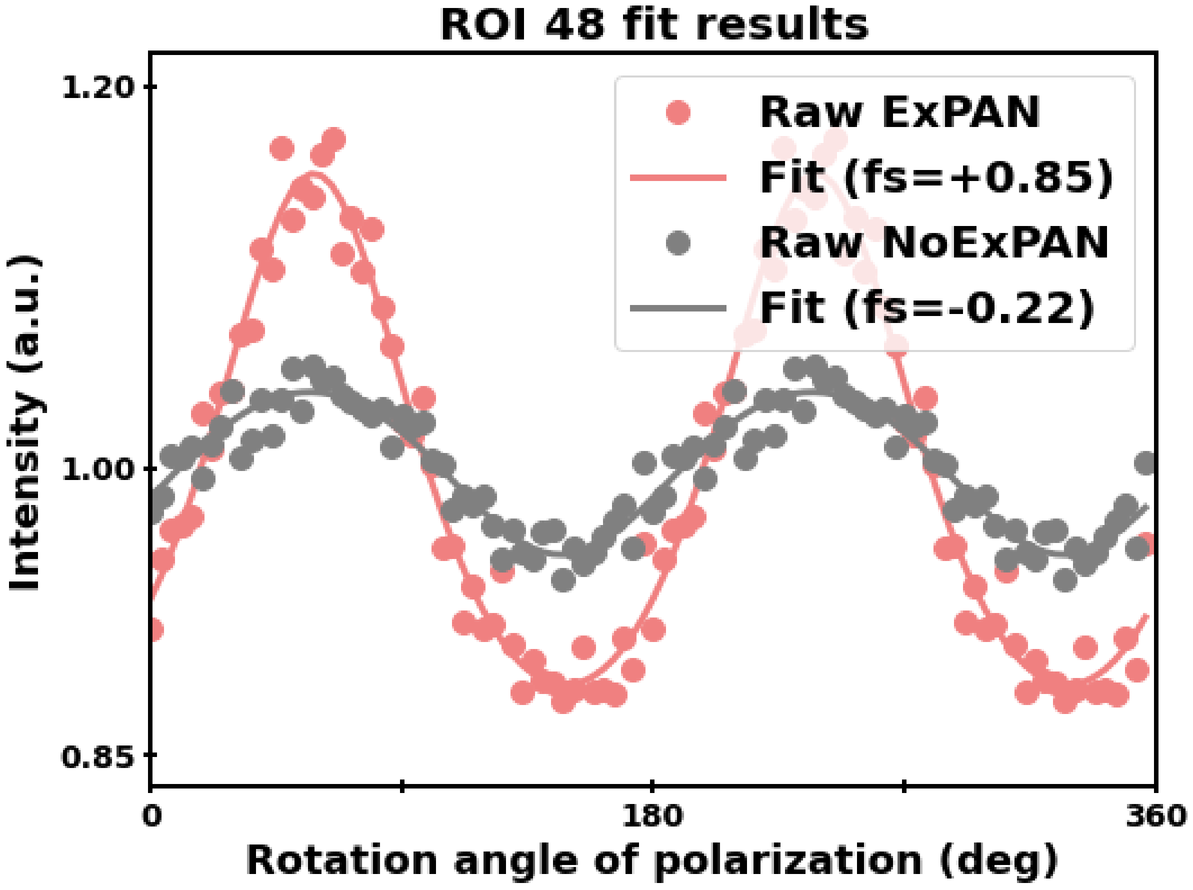
ROI 48 fit results
Raw ExPAN
Fit (fs=+0.85)
Raw NoExPAN
Fit (fs=-0.22)
1.20
1.00
0.85
Intensity (a.u.)
0
180
360
Rotation angle of polarization (deg)

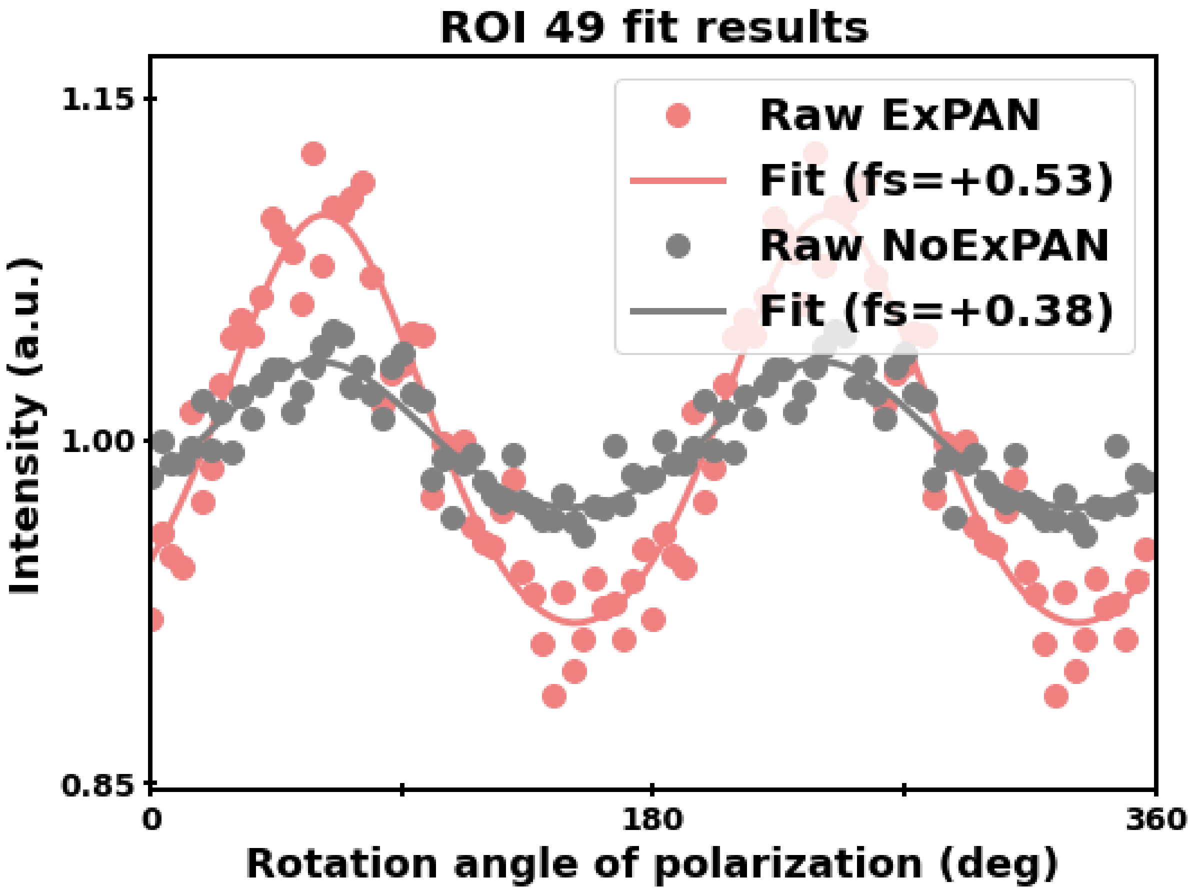

ROI 49 fit results
Raw ExPAN
Fit (fs=+0.53)
Raw NoExPAN
Fit (fs=+0.38)
Intensity (a.u.)
1.15
1.00
0.85
0
180
360
Rotation angle of polarization (deg)

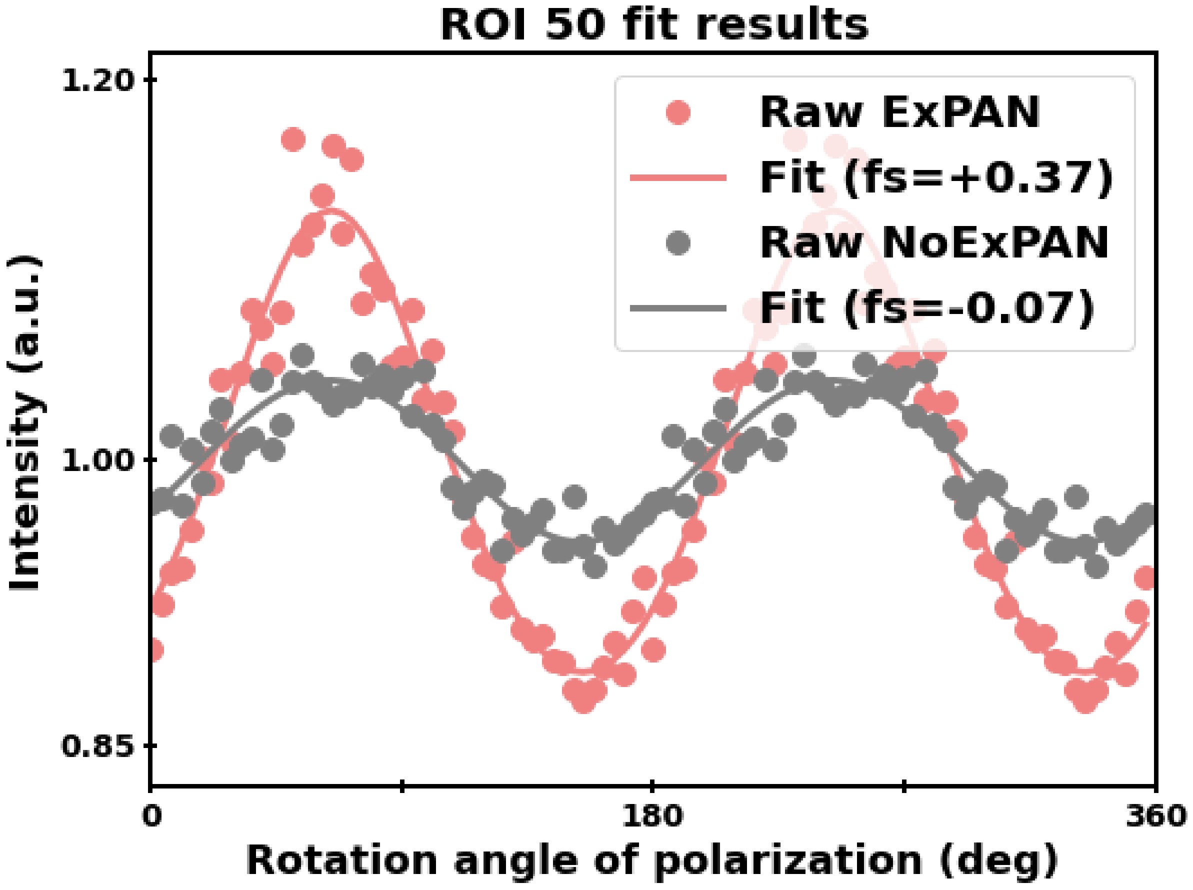

ROI 50 fit results
Raw ExPAN
Fit (fs=+0.37)
Raw NoExPAN
Fit (fs=-0.07)
Intensity (a.u.)
1.20
1.00
0.85
0
180
360
Rotation angle of polarization (deg)

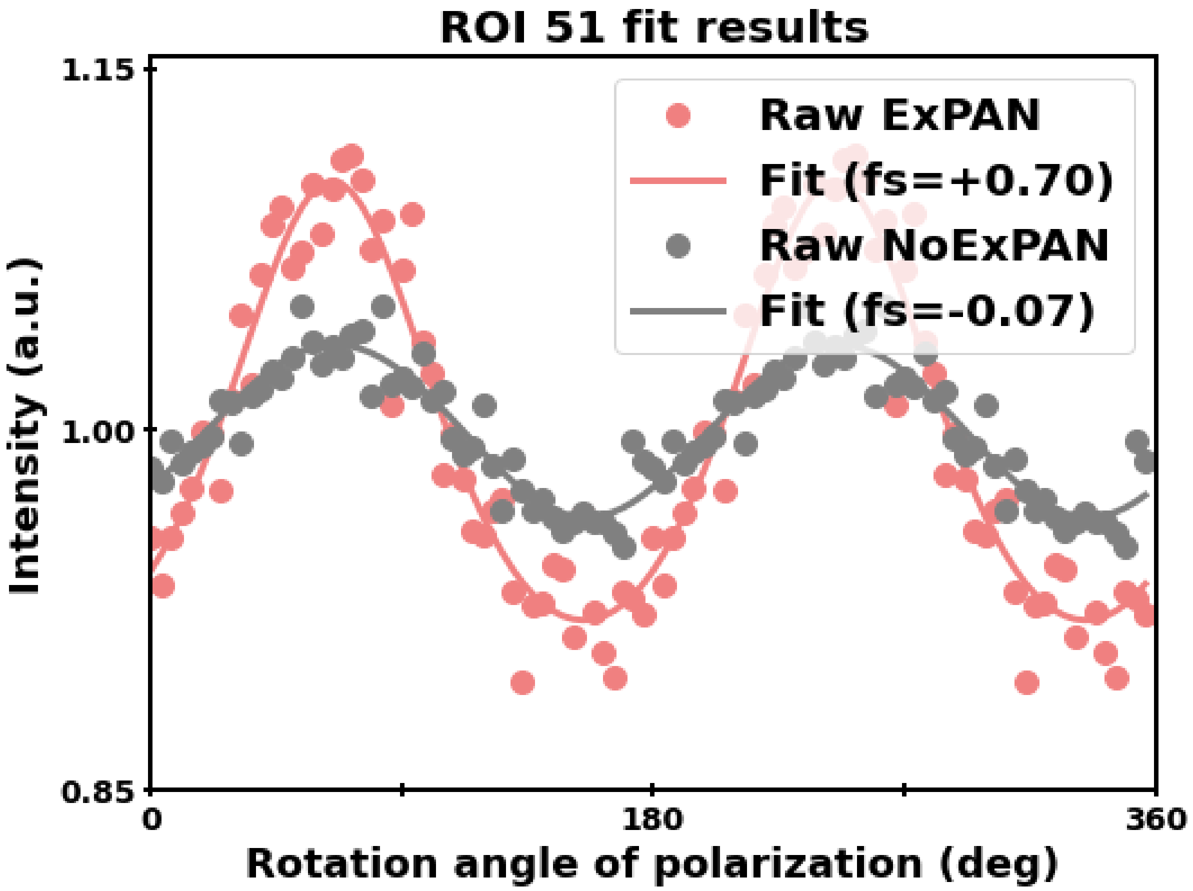
ROI 51 fit results
Raw ExPAN
Fit (fs=+0.70)
Raw NoExPAN
Fit (fs=-0.07)
Intensity (a.u.)
1.15
1.00
0.85
0
180
360
Rotation angle of polarization (deg)

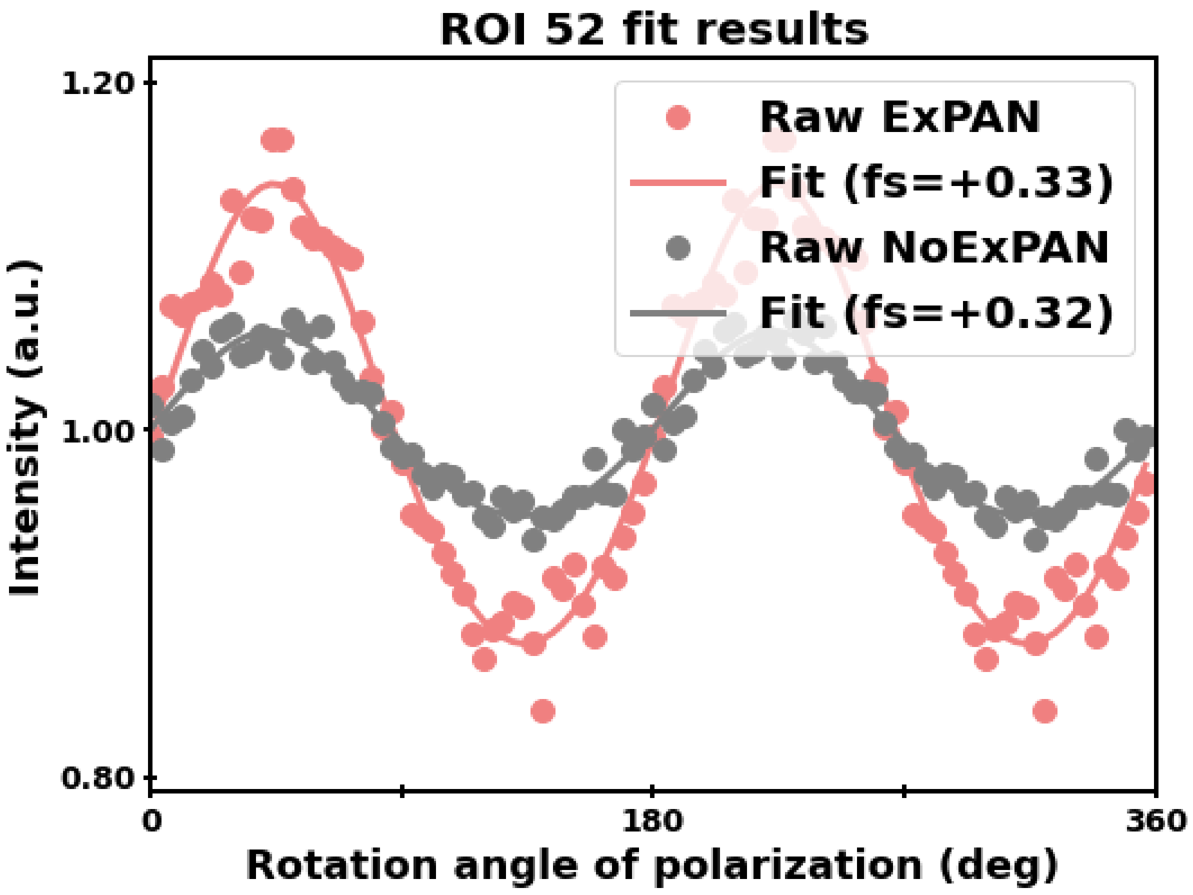
ROI 52 fit results
Raw ExPAN
Fit (fs=+0.33)
Raw NoExPAN
Fit (fs=+0.32)
Intensity (a.u.)
1.20
1.00
0.80
0
180
360
Rotation angle of polarization (deg)

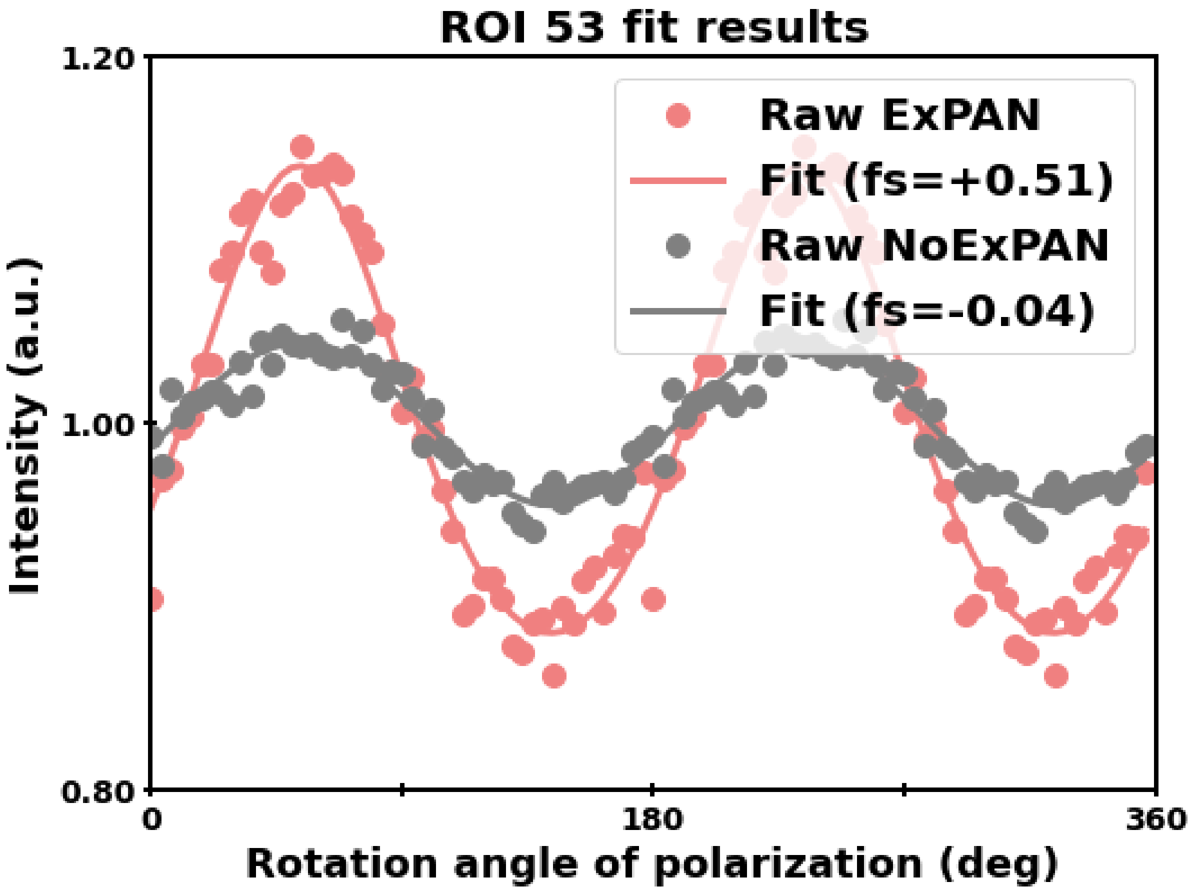
ROI 53 fit results
1.20
1.00
0.80
0
180
360
Intensity (a.u.)
Rotation angle of polarization (deg)
Raw ExPAN
Fit (fs=+0.51)
Raw NoExPAN
Fit (fs=-0.04)

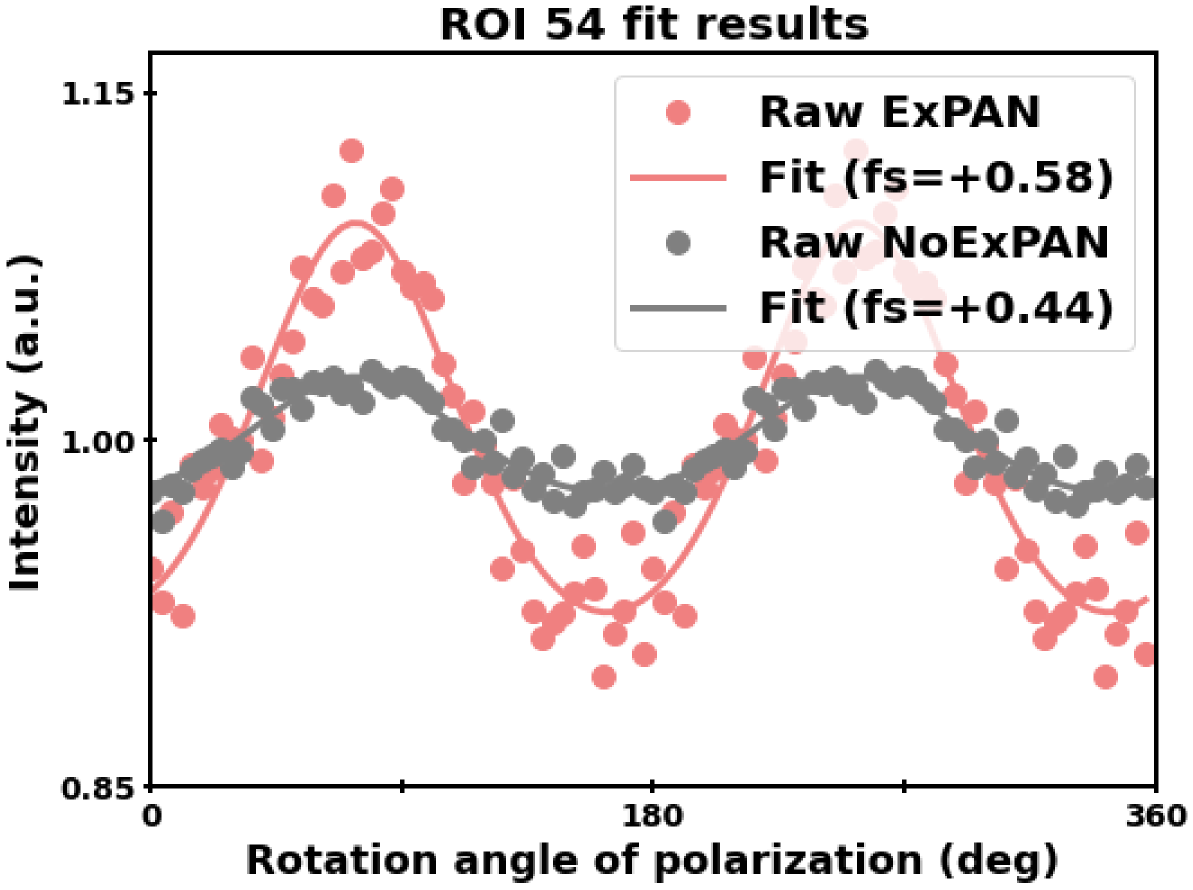
ROI 54 fit results
1.15
1.00
0.85
0
180
360
Intensity (a.u.)
Rotation angle of polarization (deg)
Raw ExPAN
Fit (fs=+0.58)
Raw NoExPAN
Fit (fs=+0.44)

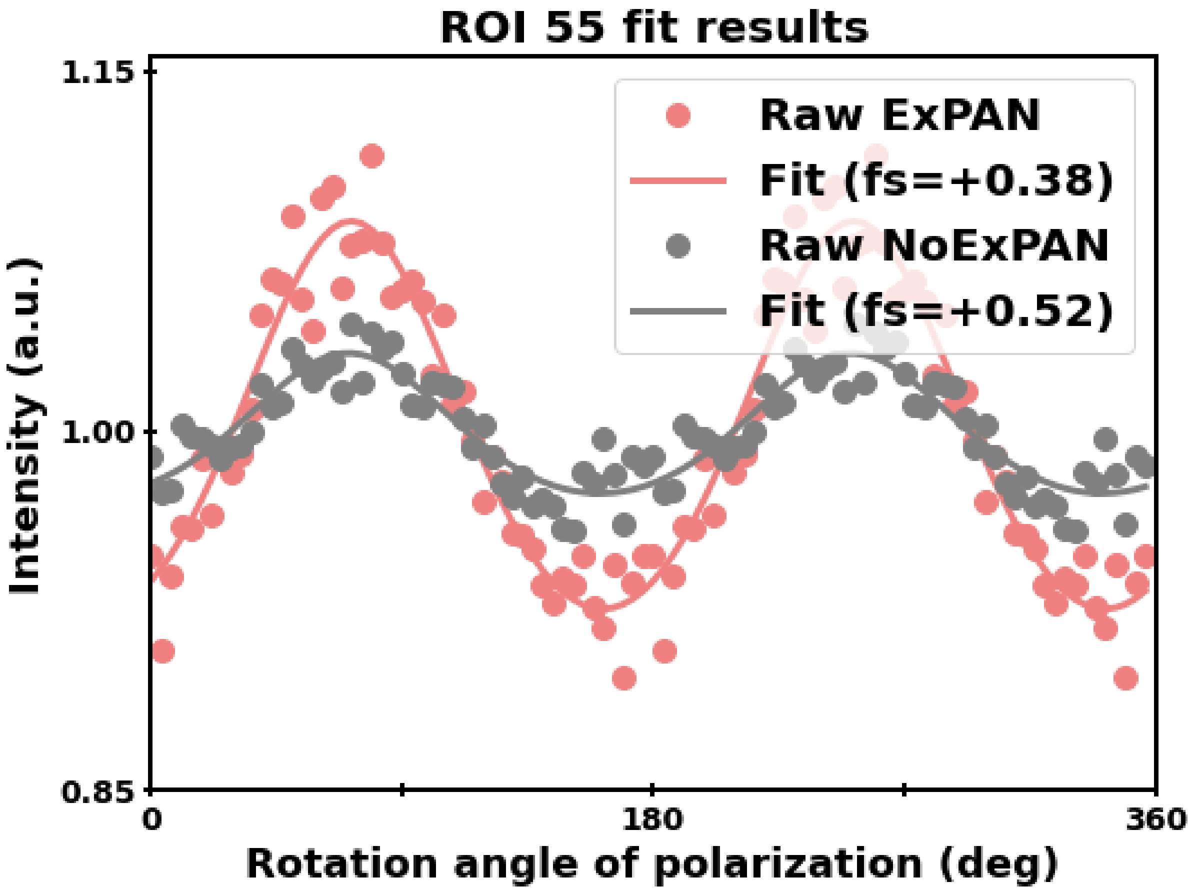
ROI 55 fit results
Raw ExPAN
Fit (fs=+0.38)
Raw NoExPAN
Fit (fs=+0.52)
Intensity (a.u.)
1.15
1.00
0.85
0
180
360
Rotation angle of polarization (deg)

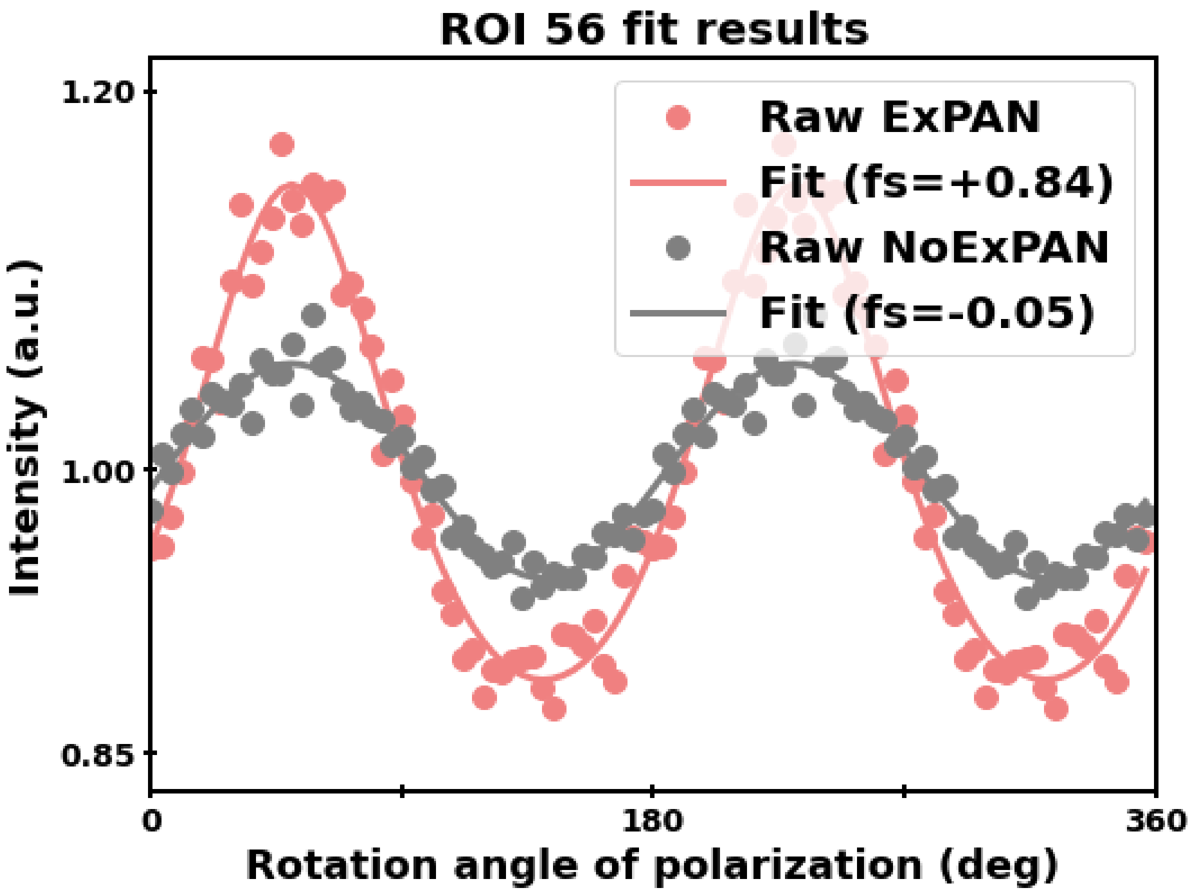
ROI 56 fit results
Raw ExPAN
Fit (fs=+0.84)
Raw NoExPAN
Fit (fs=-0.05)
Intensity (a.u.)
1.20
1.00
0.85
0
180
360
Rotation angle of polarization (deg)

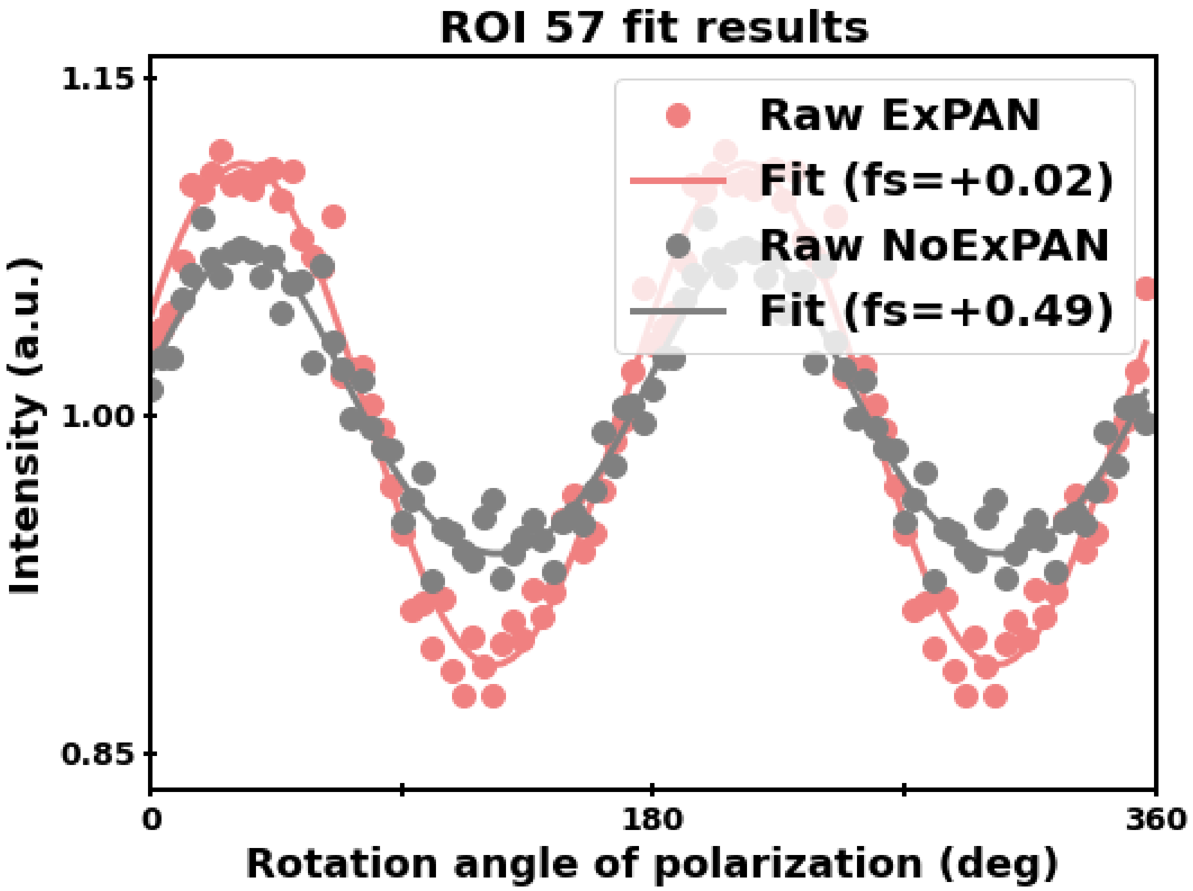
ROI 57 fit results
Raw ExPAN
Fit (fs=+0.02)
Raw NoExPAN
Fit (fs=+0.49)
Intensity (a.u.)
1.15
1.00
0.85
0
180
360
Rotation angle of polarization (deg)

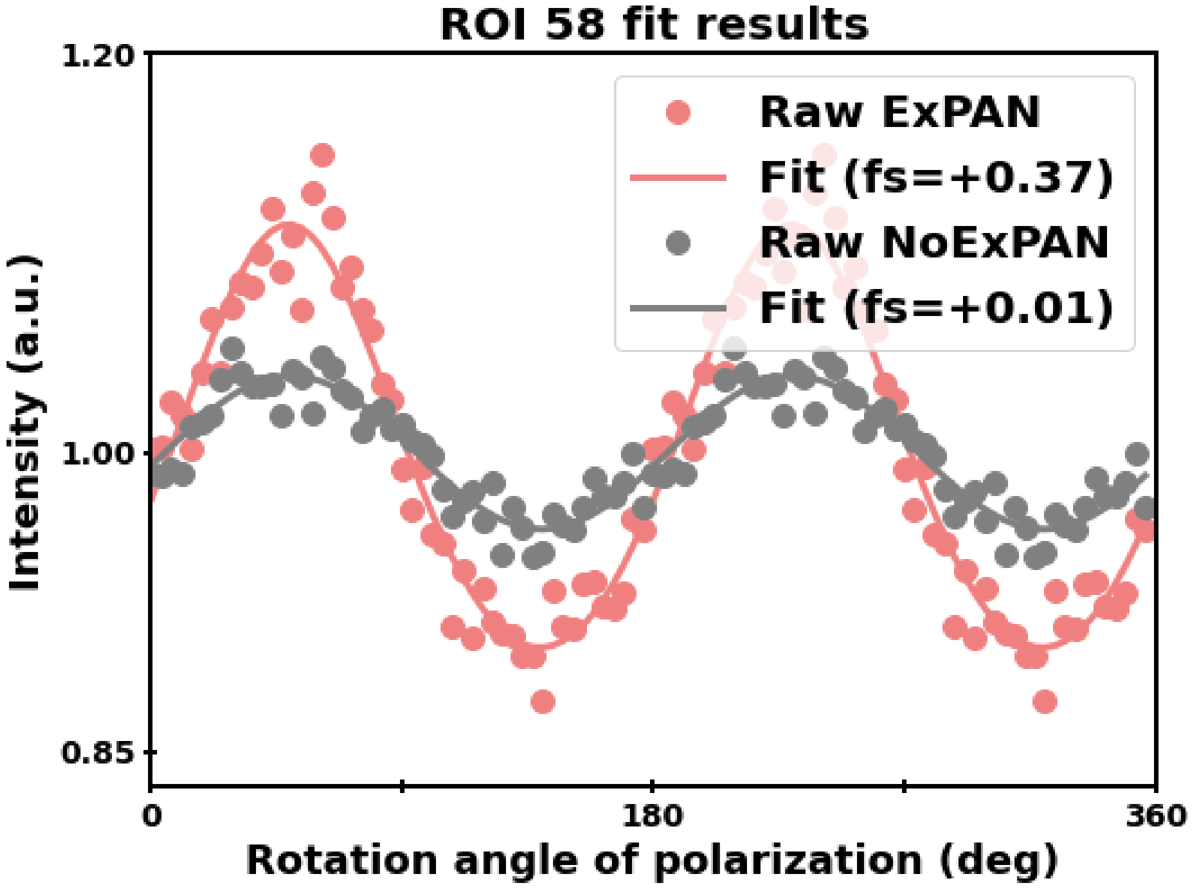
ROI 58 fit results
Raw ExPAN
Fit (fs=+0.37)
Raw NoExPAN
Fit (fs=+0.01)
Intensity (a.u.)
1.20
1.00
0.85
0
180
360
Rotation angle of polarization (deg)

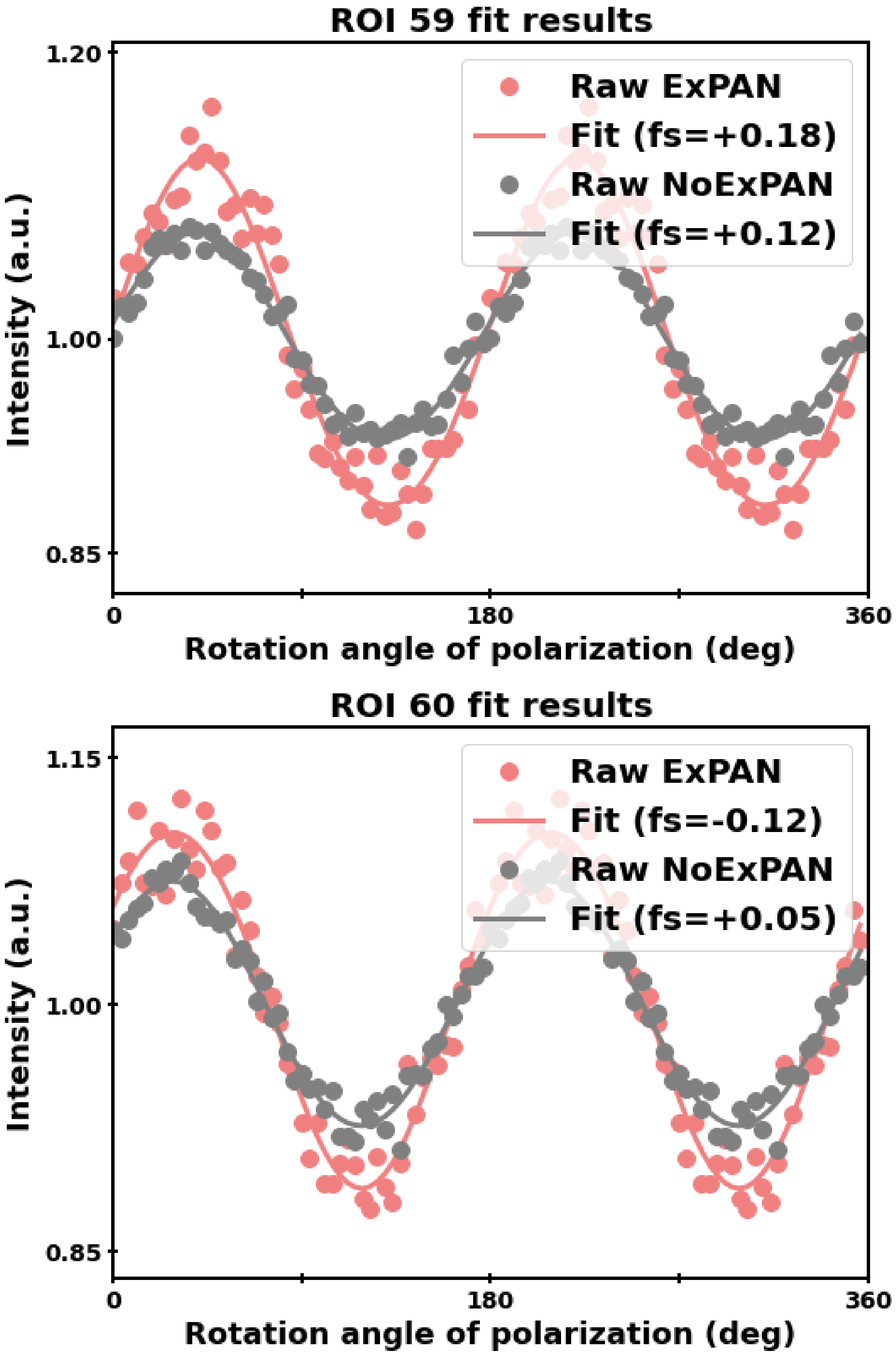
ROI 59 fit results
Raw ExPAN
Fit (fs=+0.18)
Raw NoExPAN
Fit (fs=+0.12)
Intensity (a.u.)
1.20
1.00
0.85
0
180
360
Rotation angle of polarization (deg)
ROI 60 fit results
Raw ExPAN
Fit (fs=-0.12)
Raw NoExPAN
Fit (fs=+0.05)
Intensity (a.u.)
1.15
1.00
0.85
0
180
360
Rotation angle of polarization (deg)

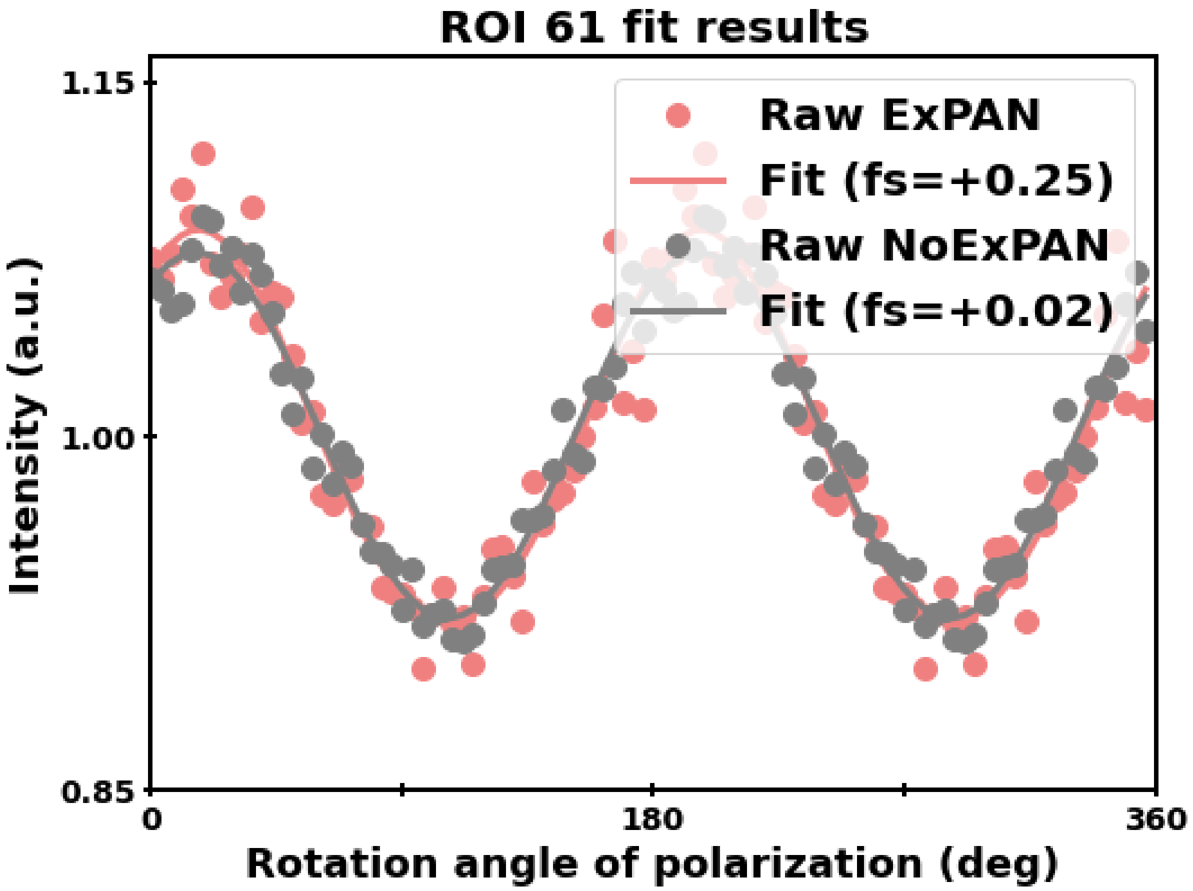
ROI 61 fit results
Raw ExPAN
Fit (fs=+0.25)
Raw NoExPAN
Fit (fs=+0.02)
Intensity (a.u.)
1.15
1.00
0.85
0
180
360
Rotation angle of polarization (deg)

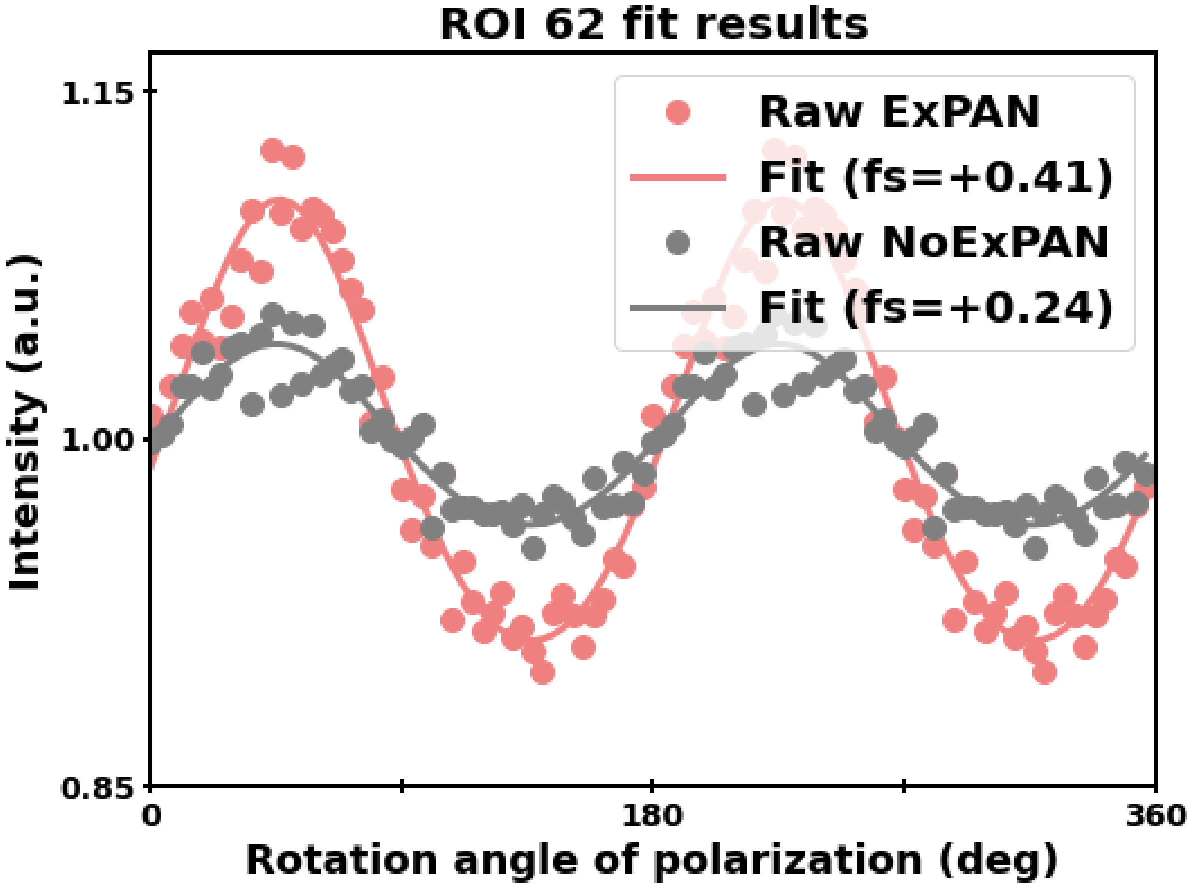
ROI 62 fit results
Raw ExPAN
Fit (fs=+0.41)
Raw NoExPAN
Fit (fs=+0.24)
Intensity (a.u.)
1.15
1.00
0.85
0
180
360
Rotation angle of polarization (deg)

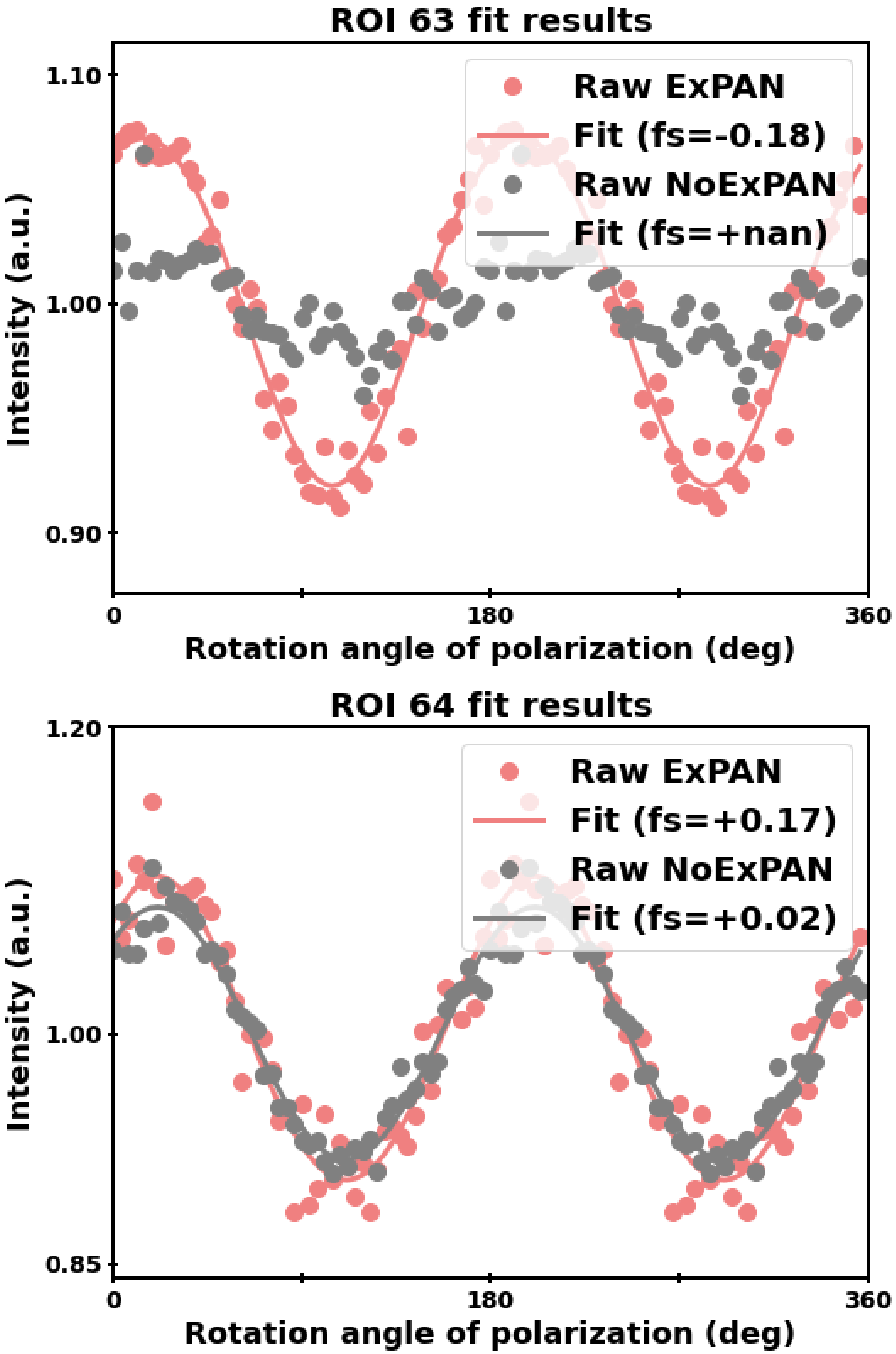
ROI 63 fit results
1.10
1.00
0.90
Intensity (a.u.)
Raw ExPAN
Fit (fs=-0.18)
Raw NoExPAN
Fit (fs=+nan)
0
180
360
Rotation angle of polarization (deg)
ROI 64 fit results
1.20
1.00
0.85
Intensity (a.u.)
Raw ExPAN
Fit (fs=+0.17)
Raw NoExPAN
Fit (fs=+0.02)
0
180
360
Rotation angle of polarization (deg)

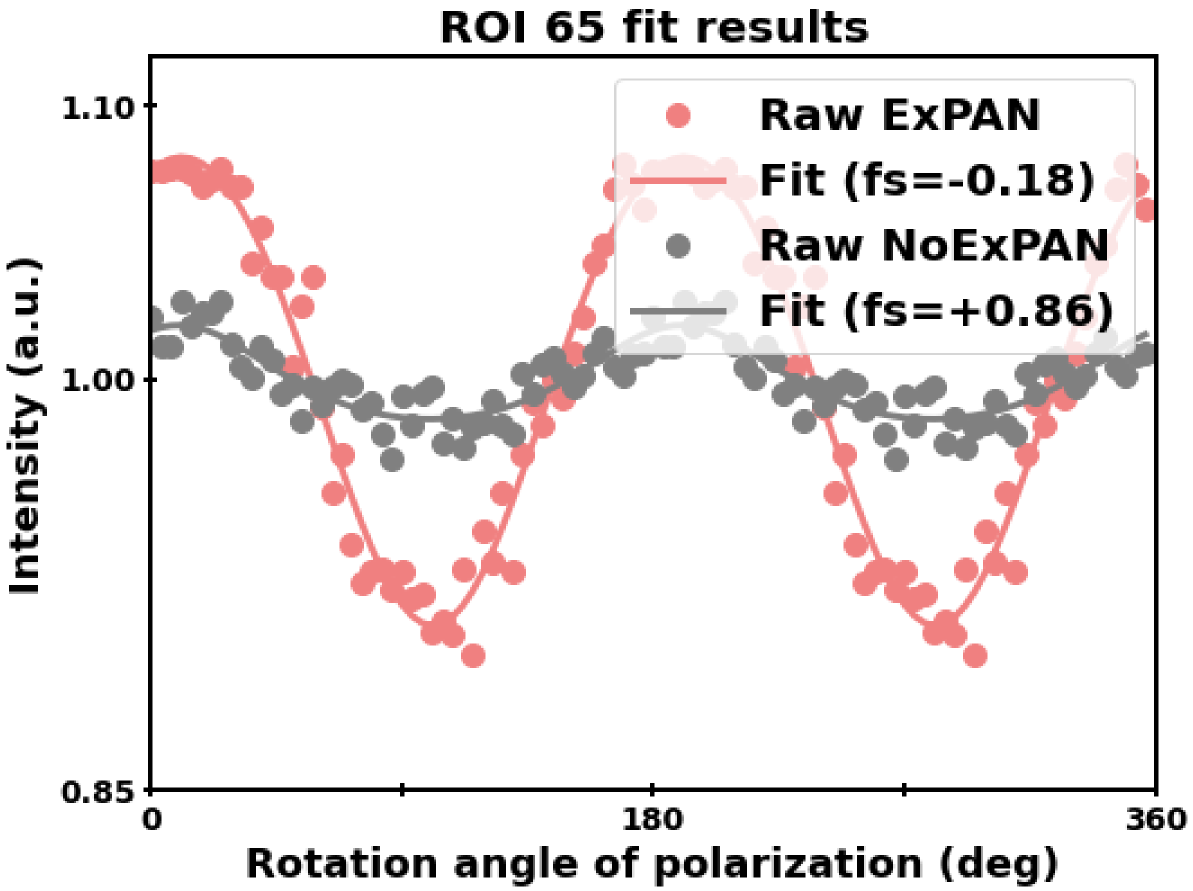
ROI 65 fit results
Raw ExPAN
Fit (fs=-0.18)
Raw NoExPAN
Fit (fs=+0.86)
1.10
1.00
0.85
0
180
360
Intensity (a.u.)
Rotation angle of polarization (deg)

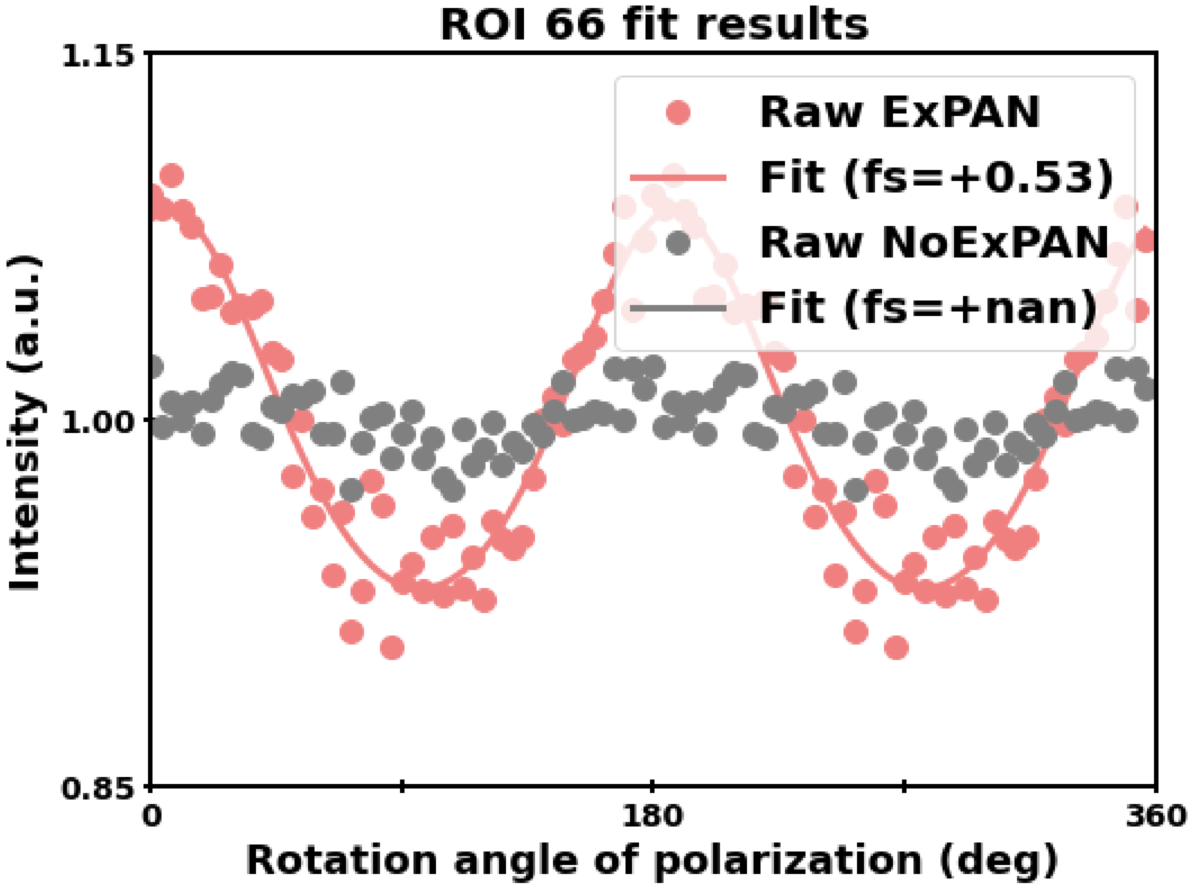
ROI 66 fit results
Raw ExPAN
Fit (fs=+0.53)
Raw NoExPAN
Fit (fs=+nan)
1.15
1.00
0.85
0
180
360
Intensity (a.u.)
Rotation angle of polarization (deg)

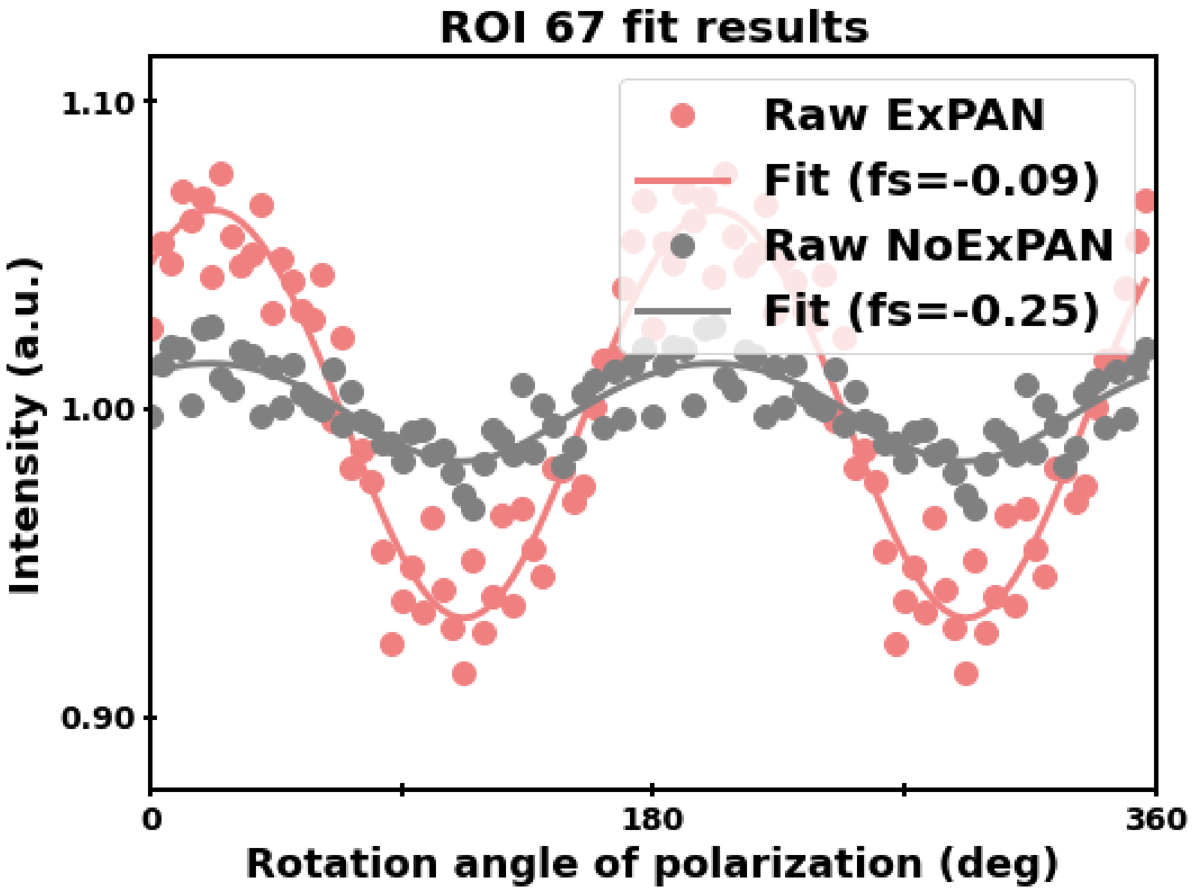
ROI 67 fit results
Raw ExPAN
Fit (fs=-0.09)
Raw NoExPAN
Fit (fs=-0.25)
Intensity (a.u.)
1.10
1.00
0.90
0
180
360
Rotation angle of polarization (deg)

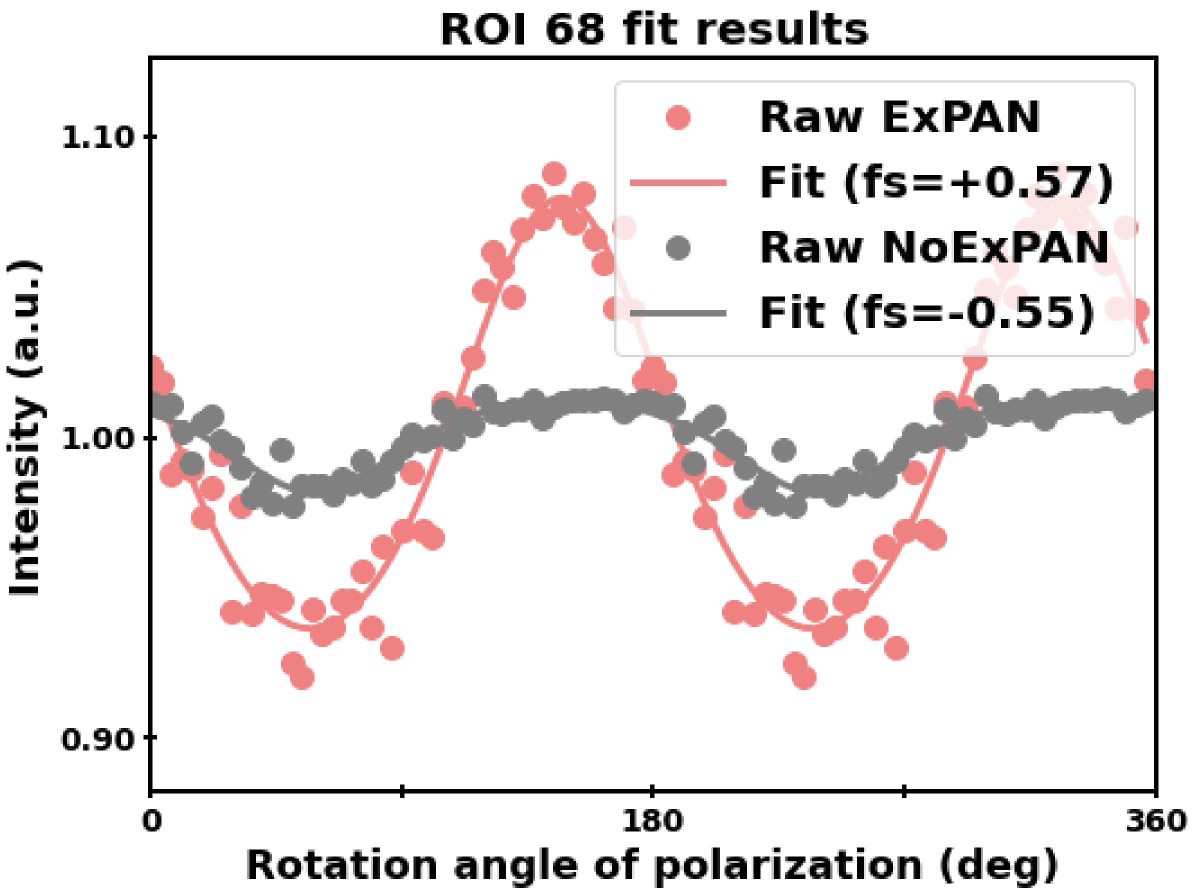
ROI 68 fit results
Raw ExPAN
Fit (fs=+0.57)
Raw NoExPAN
Fit (fs=-0.55)
Intensity (a.u.)
1.10
1.00
0.90
0
180
360
Rotation angle of polarization (deg)

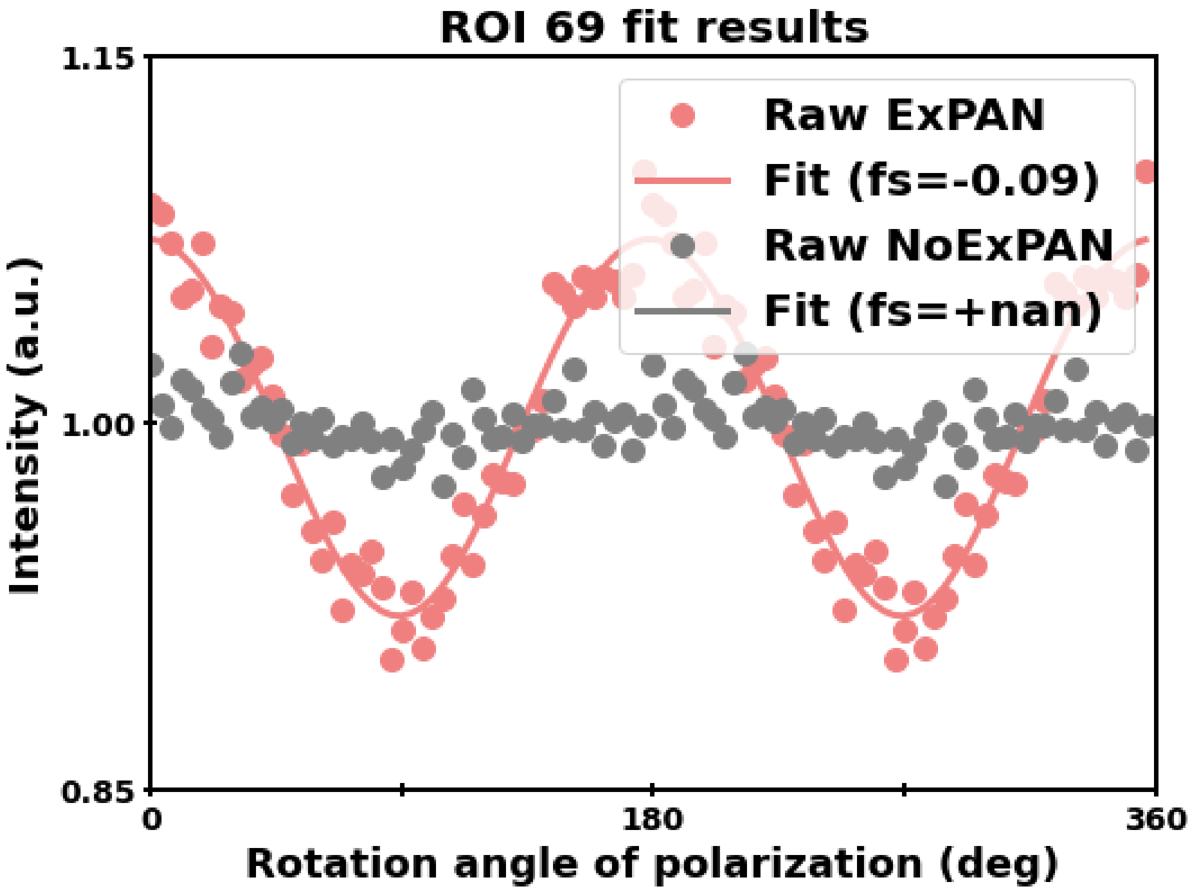
ROI 69 fit results
Raw ExPAN
Fit (fs=-0.09)
Raw NoExPAN
Fit (fs=+nan)
Intensity (a.u.)
1.15
1.00
0.85
0
180
360
Rotation angle of polarization (deg)

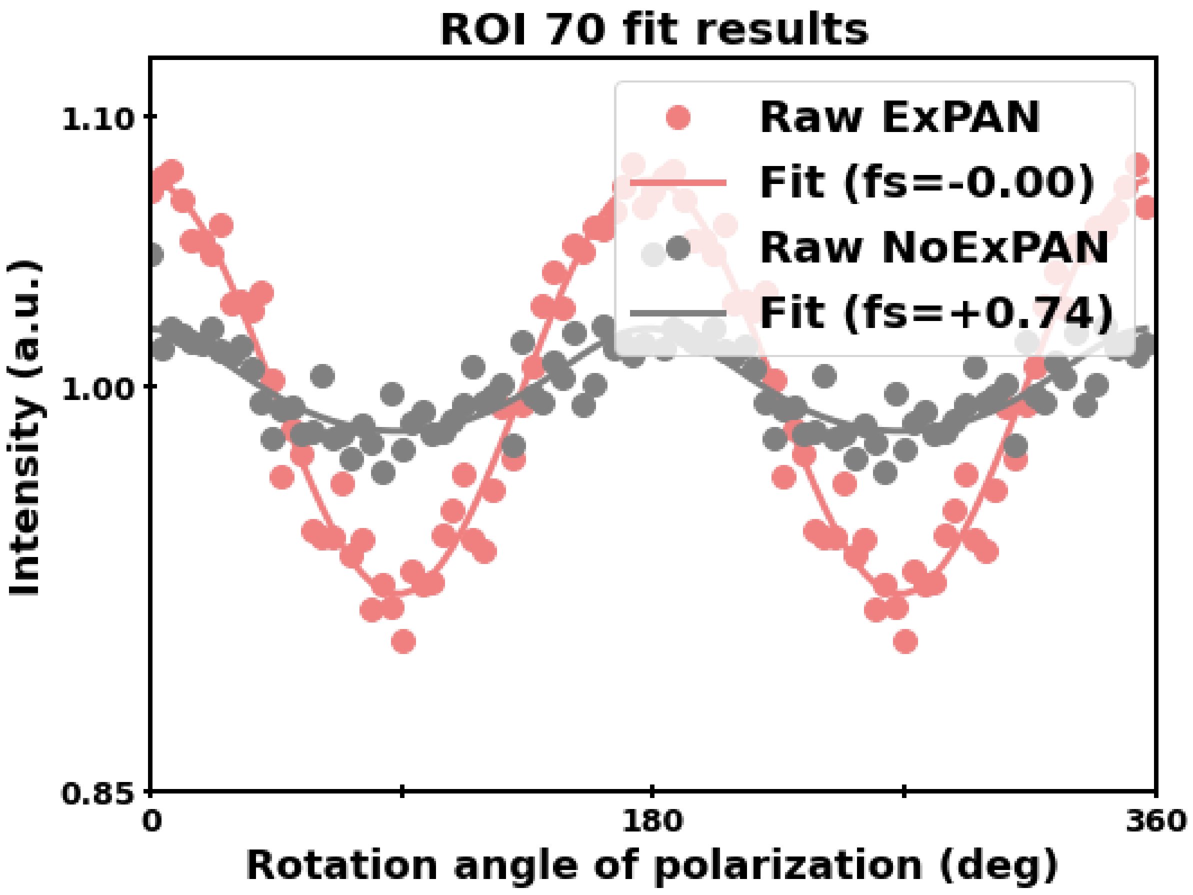
ROI 70 fit results
Raw ExPAN
Fit (fs=-0.00)
Raw NoExPAN
Fit (fs=+0.74)
Intensity (a.u.)
1.10
1.00
0.85
0
180
360
Rotation angle of polarization (deg)

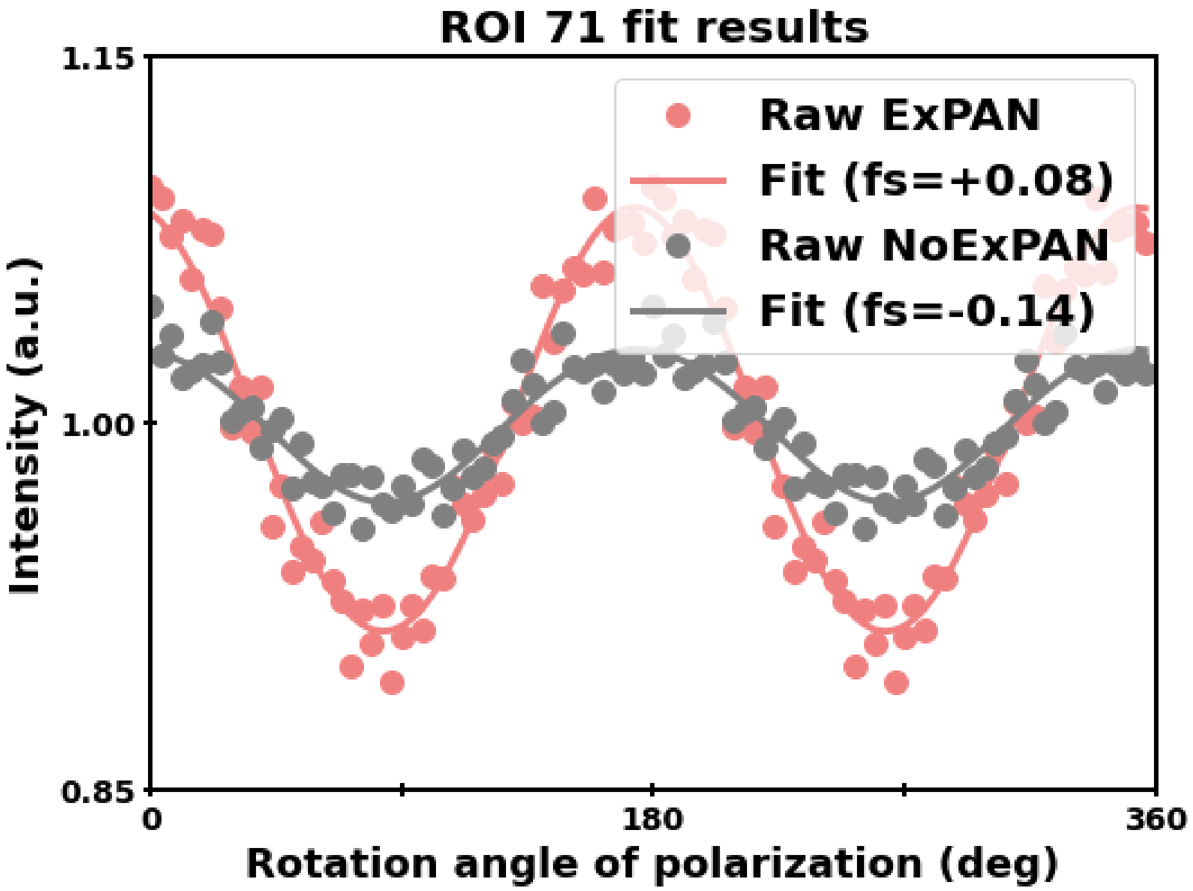

ROI 71 fit results
1.15
1.00
0.85
Intensity (a.u.)
0
180
360
Rotation angle of polarization (deg)
Raw ExPAN
Fit (fs=+0.08)
Raw NoExPAN
Fit (fs=-0.14)

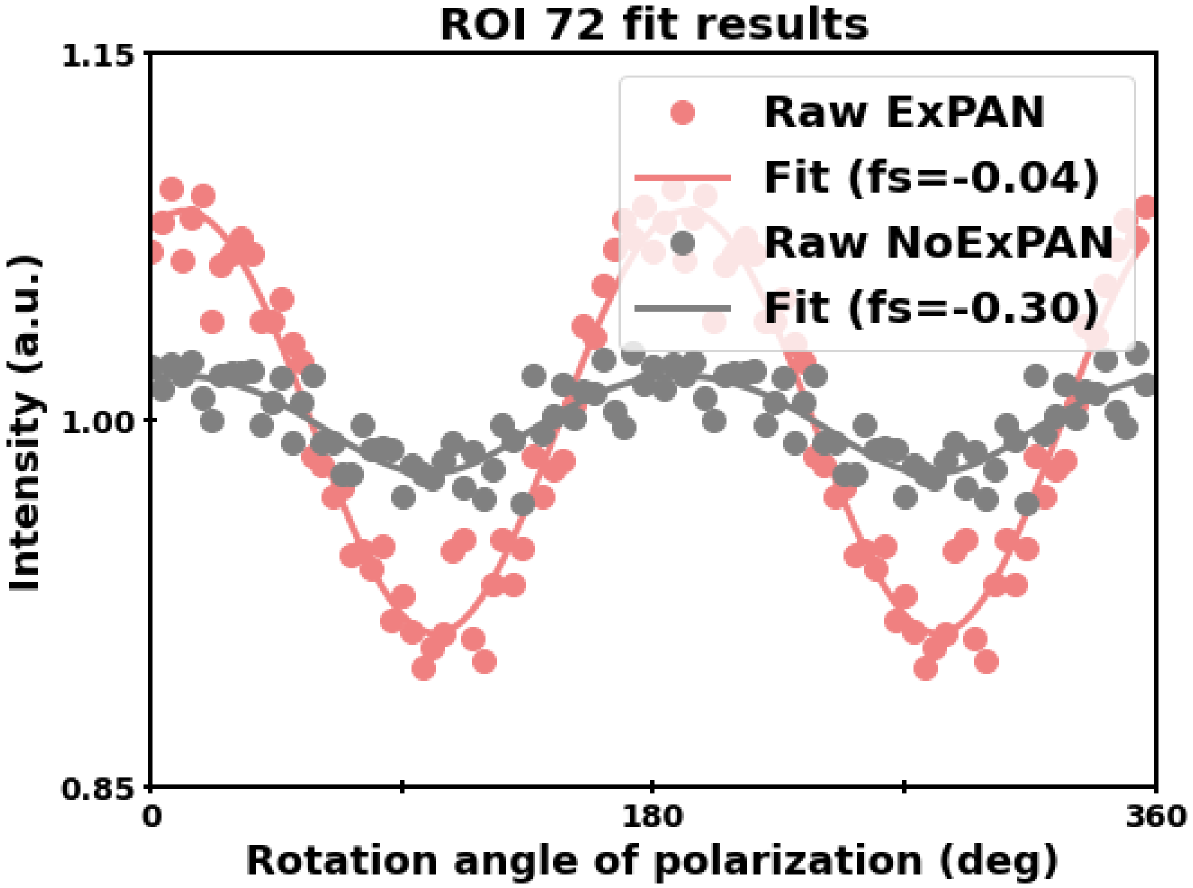

ROI 72 fit results
1.15
1.00
0.85
Intensity (a.u.)
0
180
360
Rotation angle of polarization (deg)
Raw ExPAN
Fit (fs=-0.04)
Raw NoExPAN
Fit (fs=-0.30)

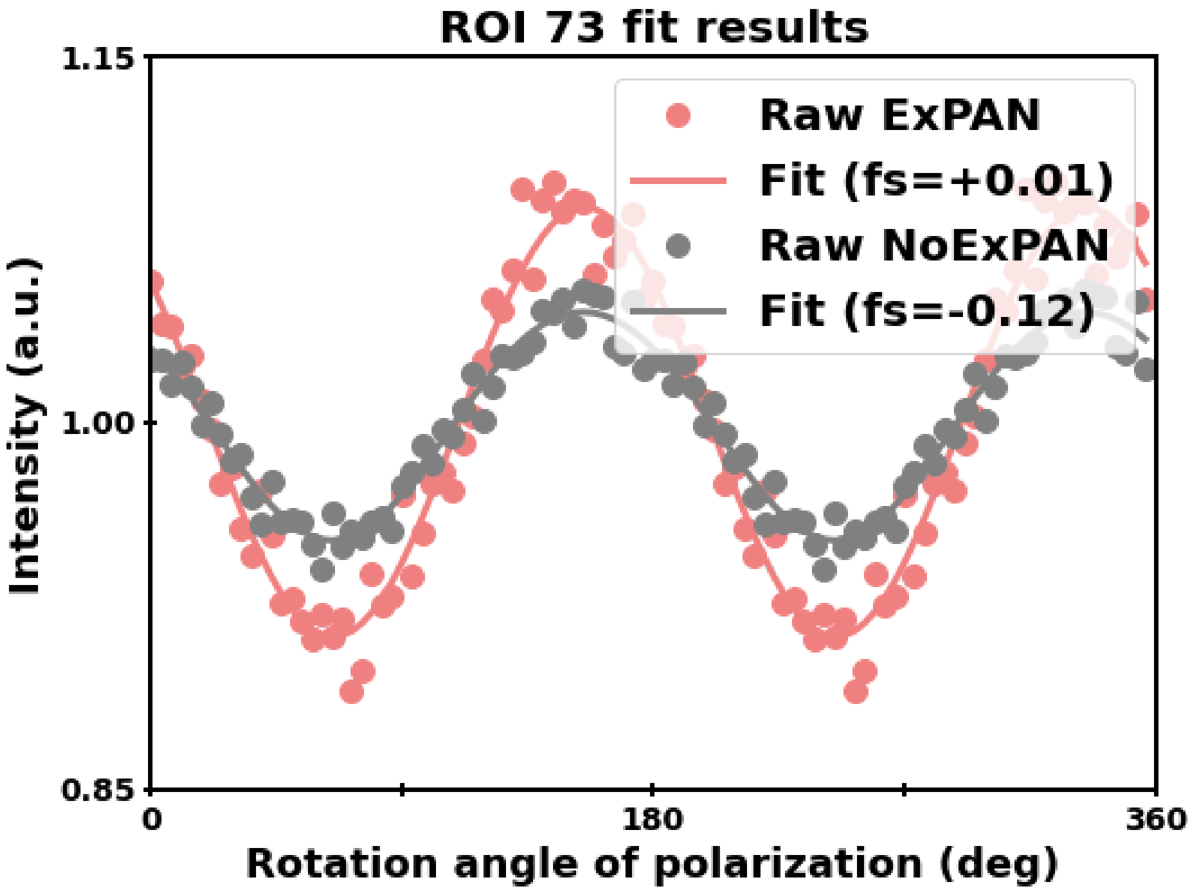

ROI 73 fit results
1.15
1.00
0.85
Intensity (a.u.)
0
180
360
Rotation angle of polarization (deg)
Raw ExPAN
Fit (fs=+0.01)
Raw NoExPAN
Fit (fs=-0.12)

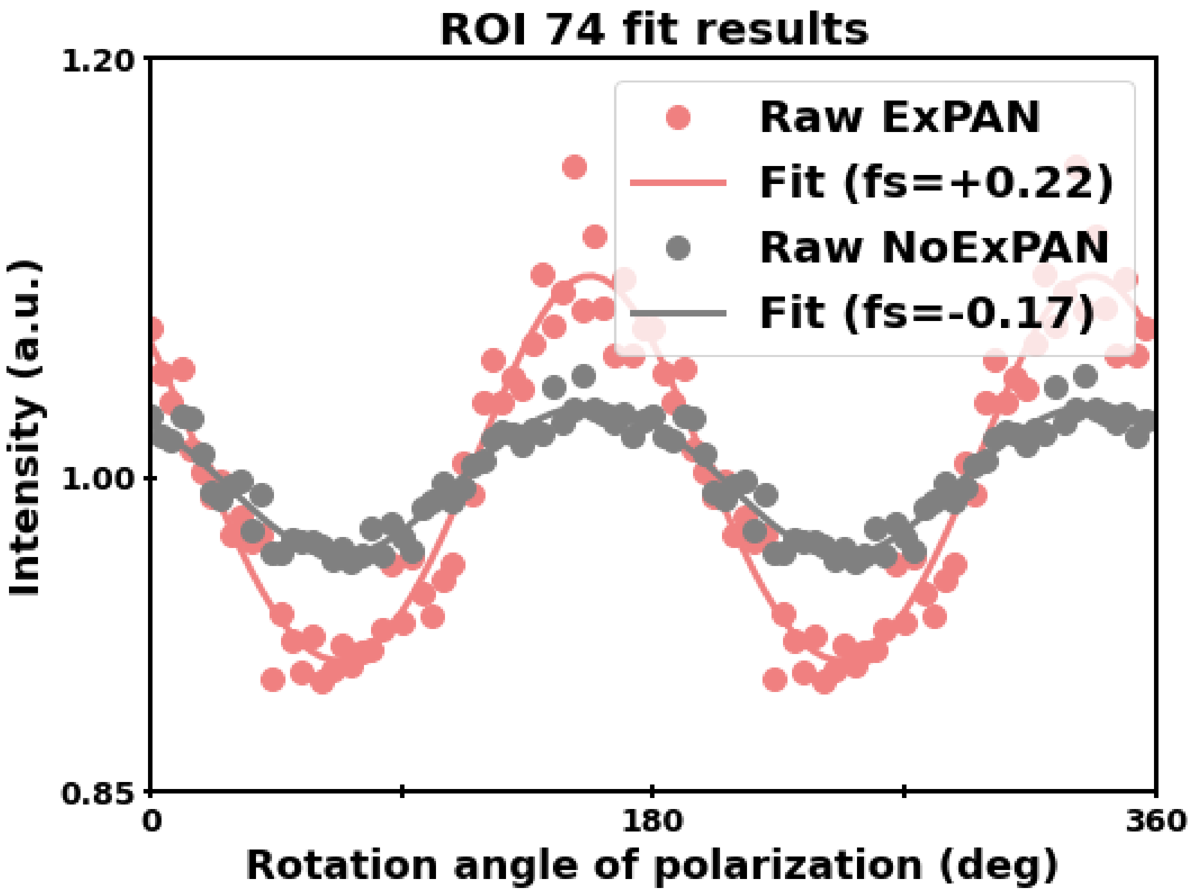

ROI 74 fit results
1.20
1.00
0.85
Intensity (a.u.)
0
180
360
Rotation angle of polarization (deg)
Raw ExPAN
Fit (fs=+0.22)
Raw NoExPAN
Fit (fs=-0.17)

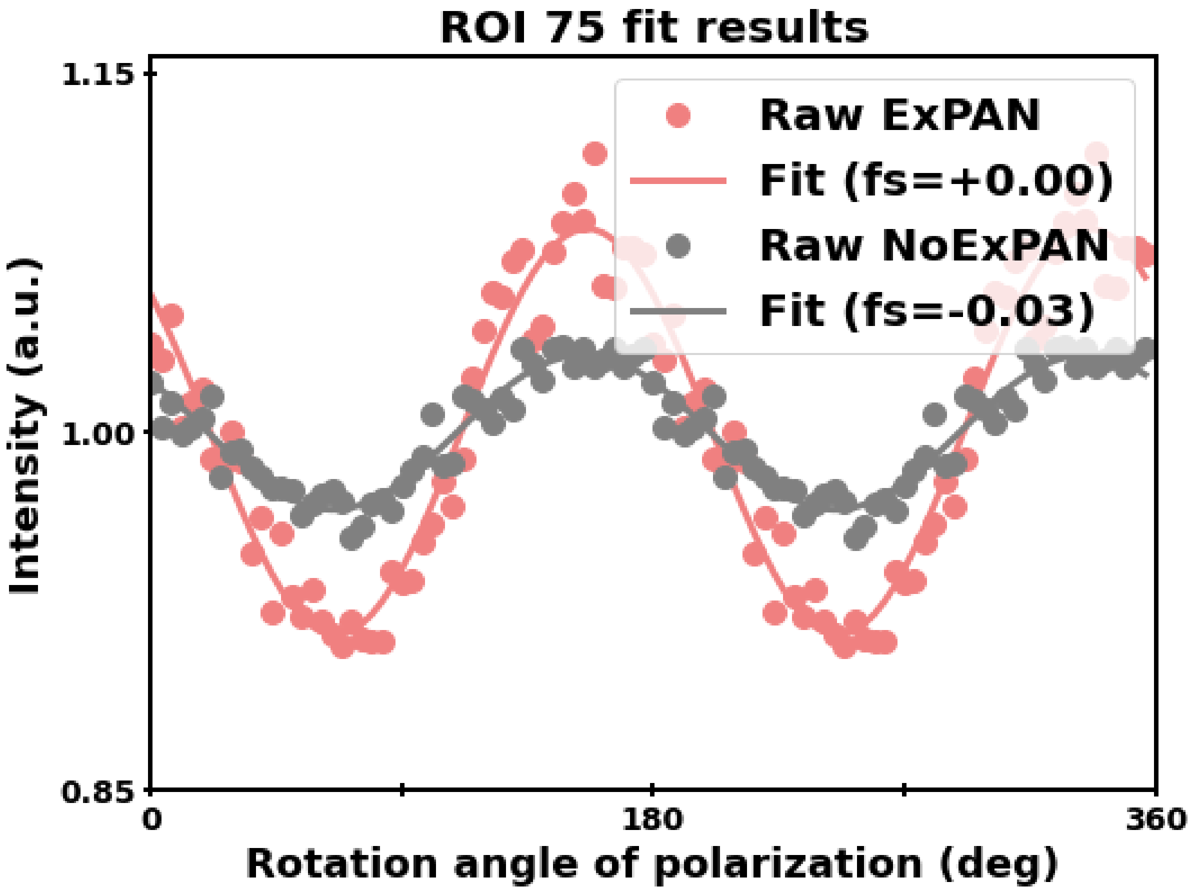
ROI 75 fit results
1.15
1.00
0.85
Intensity (a.u.)
0
180
360
Rotation angle of polarization (deg)
Raw ExPAN
Fit (fs=+0.00)
Raw NoExPAN
Fit (fs=-0.03)

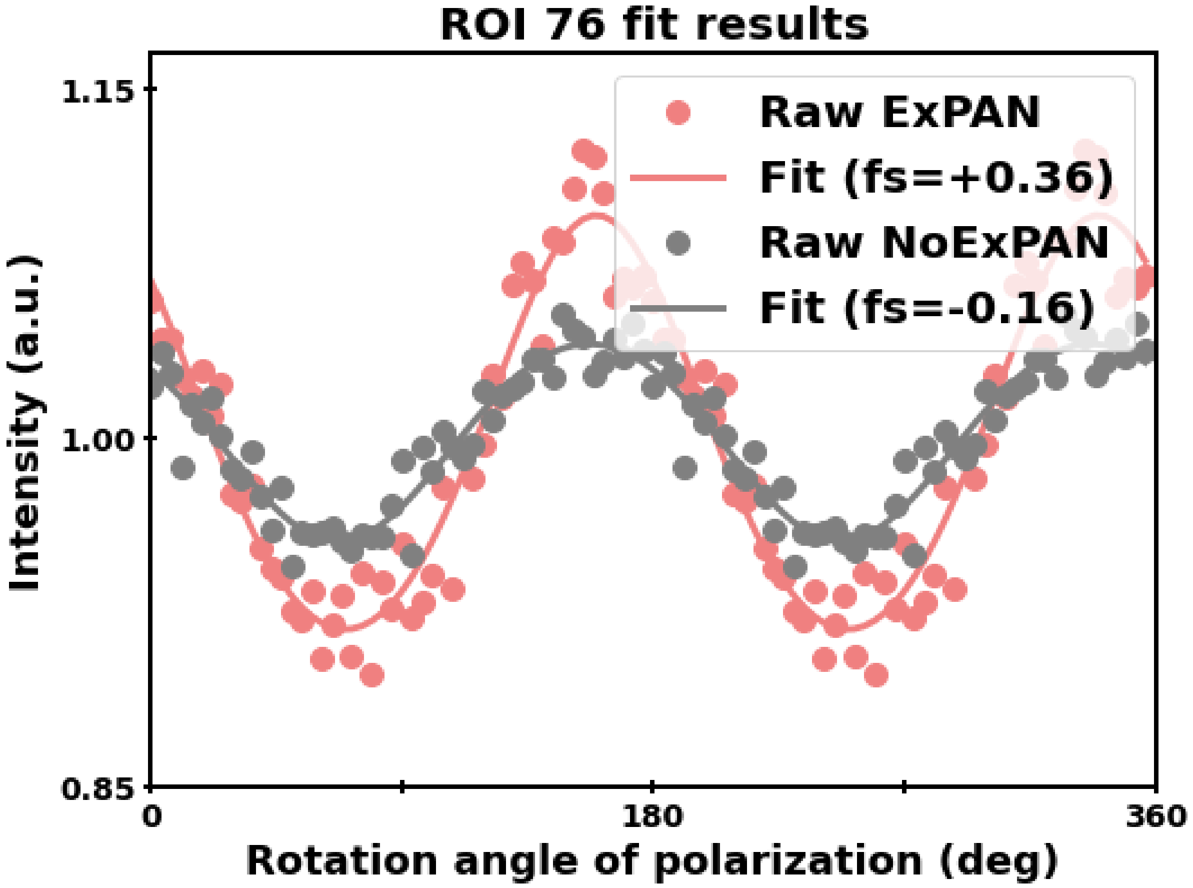
ROI 76 fit results
1.15
1.00
0.85
Intensity (a.u.)
0
180
360
Rotation angle of polarization (deg)
Raw ExPAN
Fit (fs=+0.36)
Raw NoExPAN
Fit (fs=-0.16)

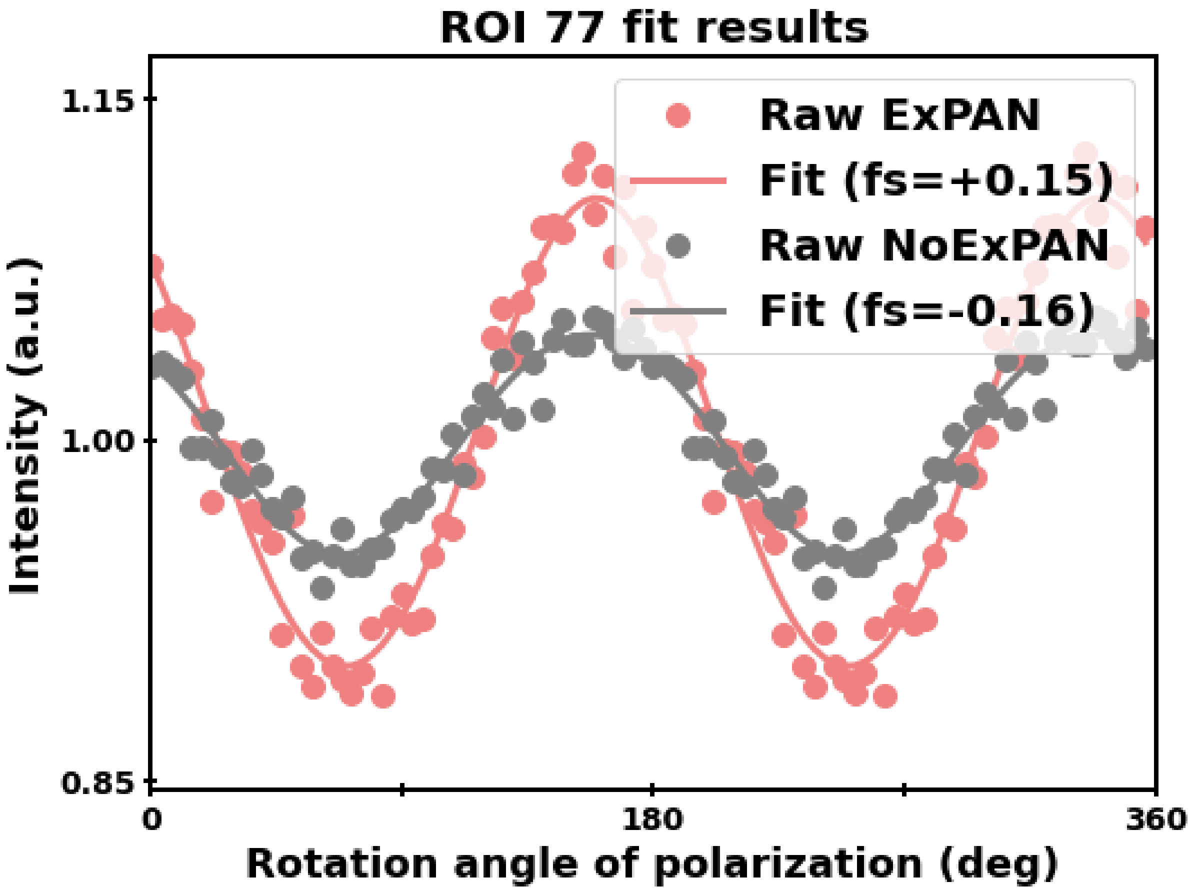
ROI 77 fit results
Raw ExPAN
Fit (fs=+0.15)
Raw NoExPAN
Fit (fs=-0.16)
1.15
1.00
0.85
Intensity (a.u.)
0
180
360
Rotation angle of polarization (deg)

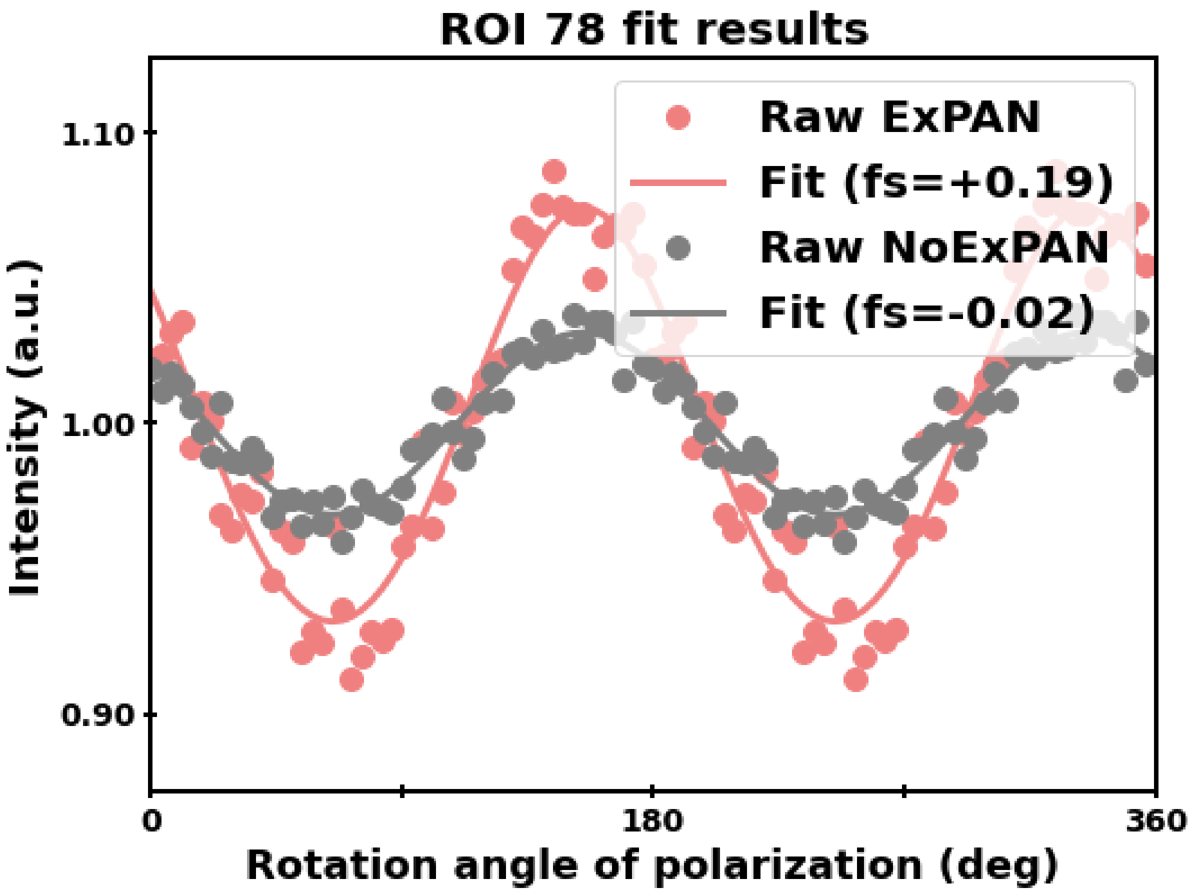
ROI 78 fit results
Raw ExPAN
Fit (fs=+0.19)
Raw NoExPAN
Fit (fs=-0.02)
1.10
1.00
0.90
Intensity (a.u.)
0
180
360
Rotation angle of polarization (deg)

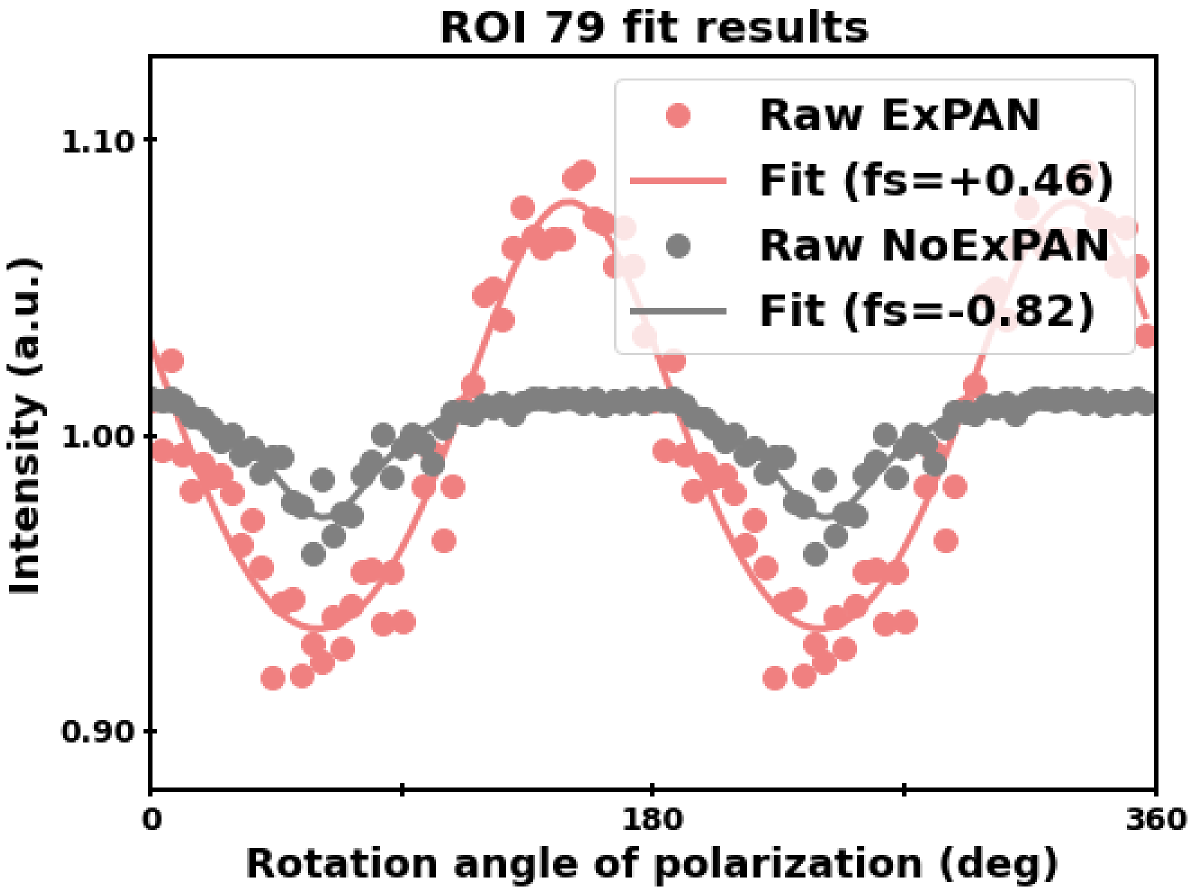
ROI 79 fit results
Raw ExPAN
Fit (fs=+0.46)
Raw NoExPAN
Fit (fs=-0.82)
Intensity (a.u.)
1.10
1.00
0.90
0
180
360
Rotation angle of polarization (deg)

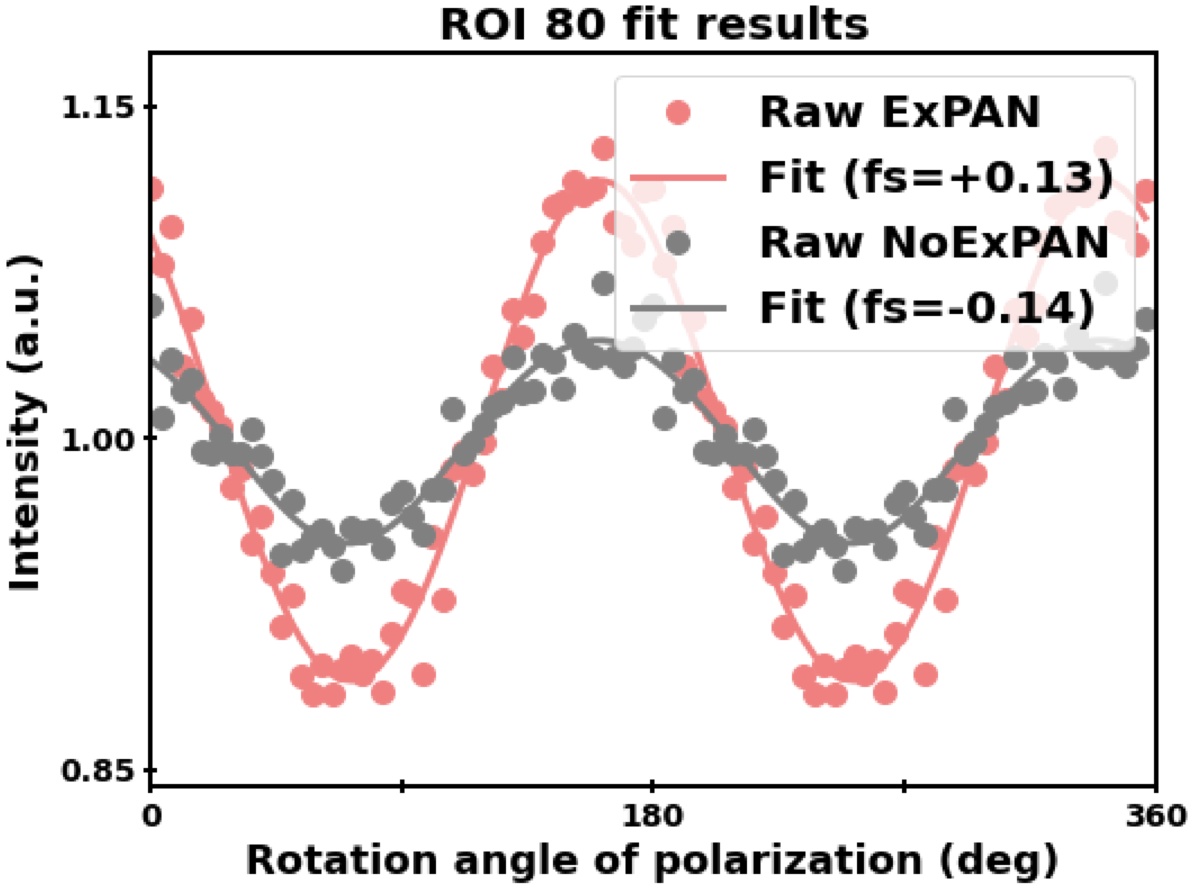
ROI 80 fit results
Raw ExPAN
Fit (fs=+0.13)
Raw NoExPAN
Fit (fs=-0.14)
Intensity (a.u.)
1.15
1.00
0.85
0
180
360
Rotation angle of polarization (deg)

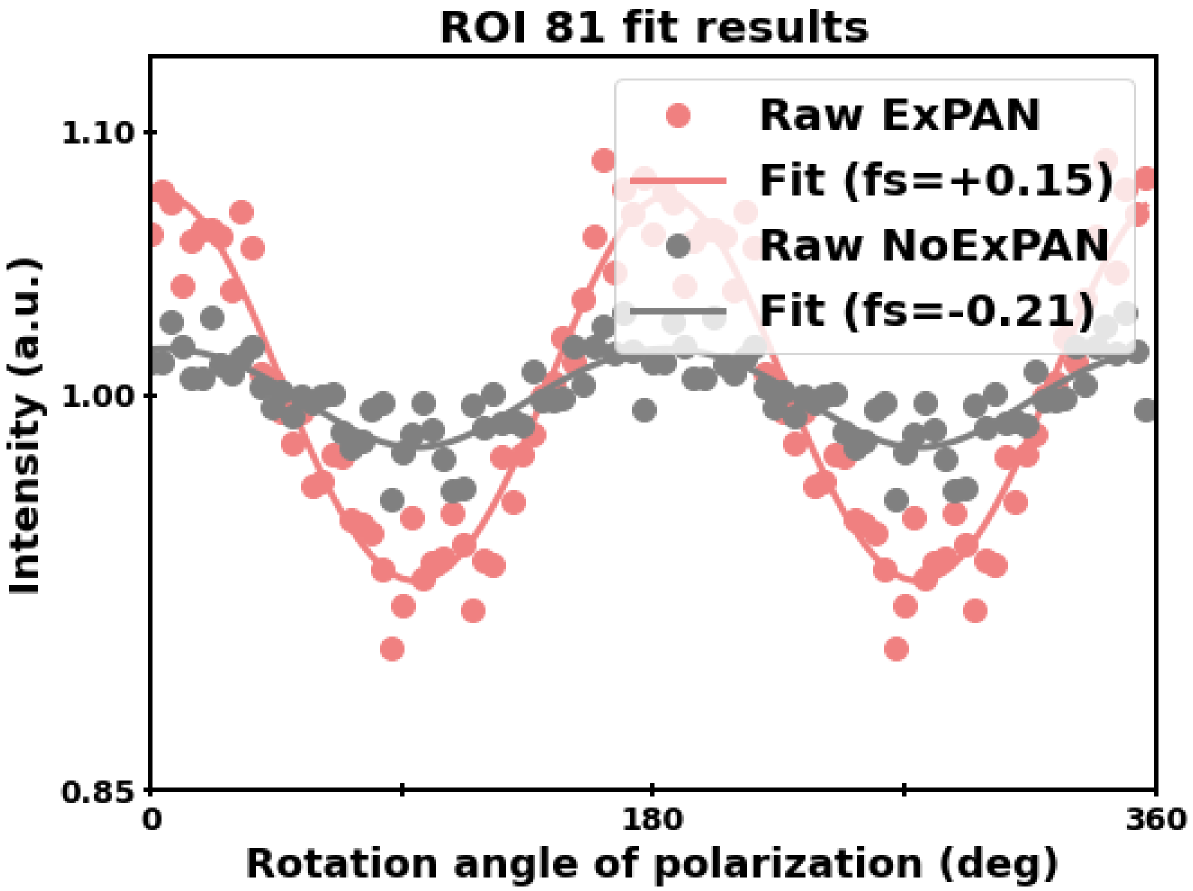
ROI 81 fit results
Raw ExPAN
Fit (fs=+0.15)
Raw NoExPAN
Fit (fs=-0.21)
1.10
1.00
0.85
0
180
360
Intensity (a.u.)
Rotation angle of polarization (deg)

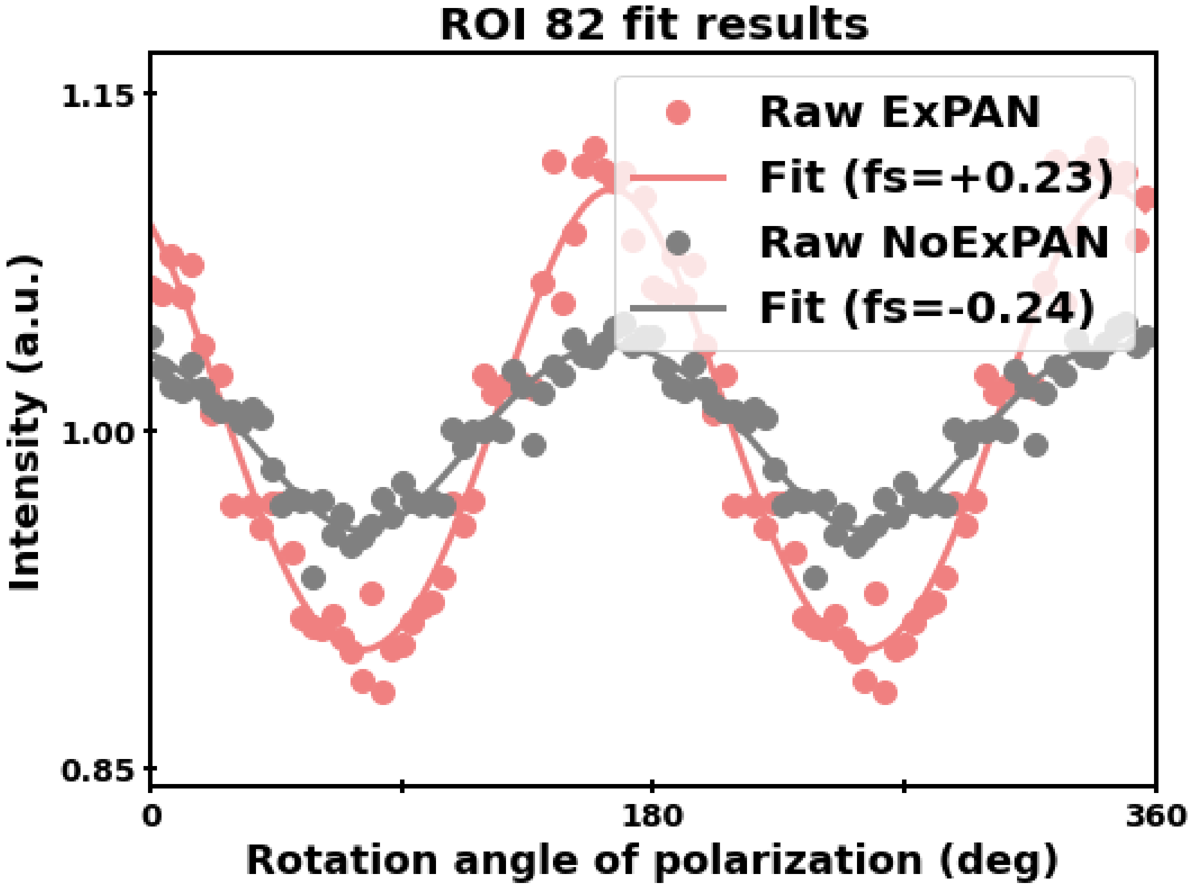
ROI 82 fit results
Raw ExPAN
Fit (fs=+0.23)
Raw NoExPAN
Fit (fs=-0.24)
1.15
1.00
0.85
0
180
360
Intensity (a.u.)
Rotation angle of polarization (deg)

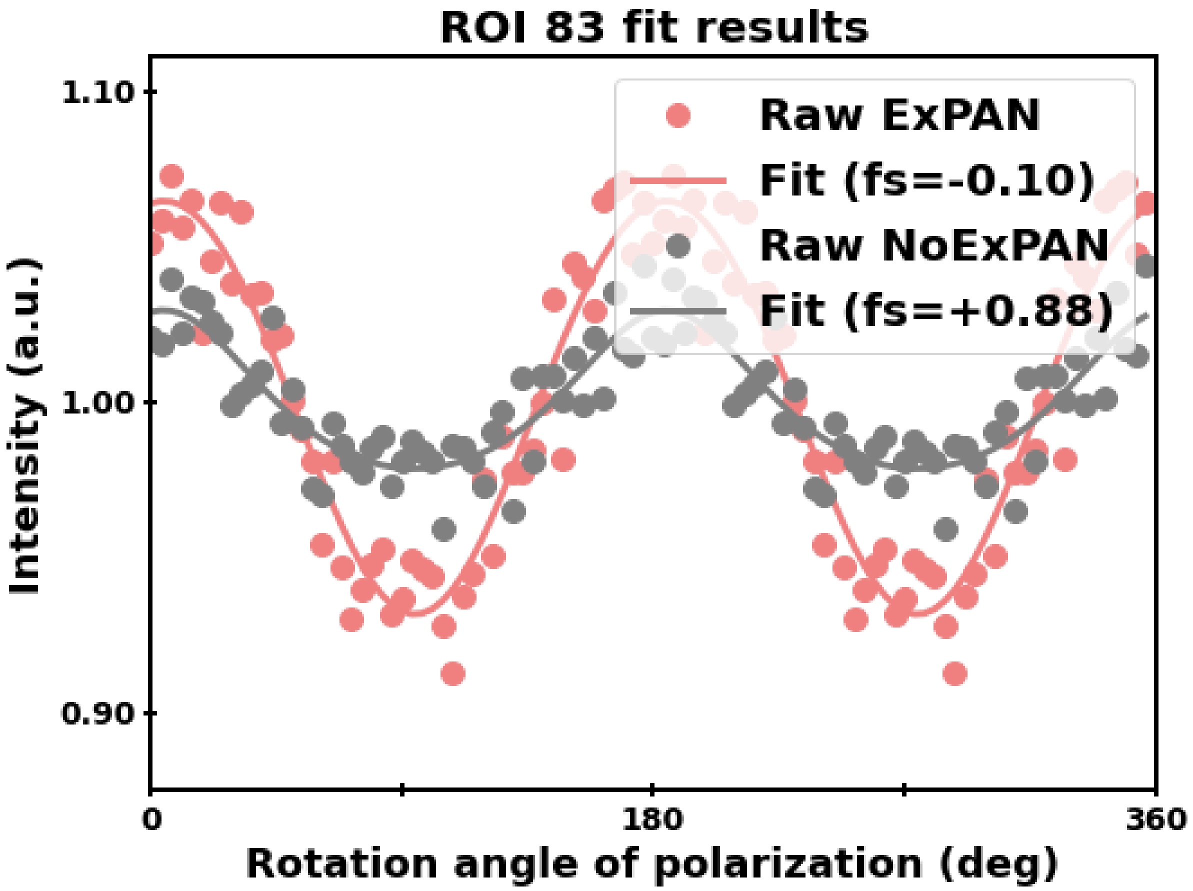
ROI 83 fit results
1.10
1.00
0.90
Intensity (a.u.)
0
180
360
Rotation angle of polarization (deg)
Raw ExPAN
Fit (fs=-0.10)
Raw NoExPAN
Fit (fs=+0.88)

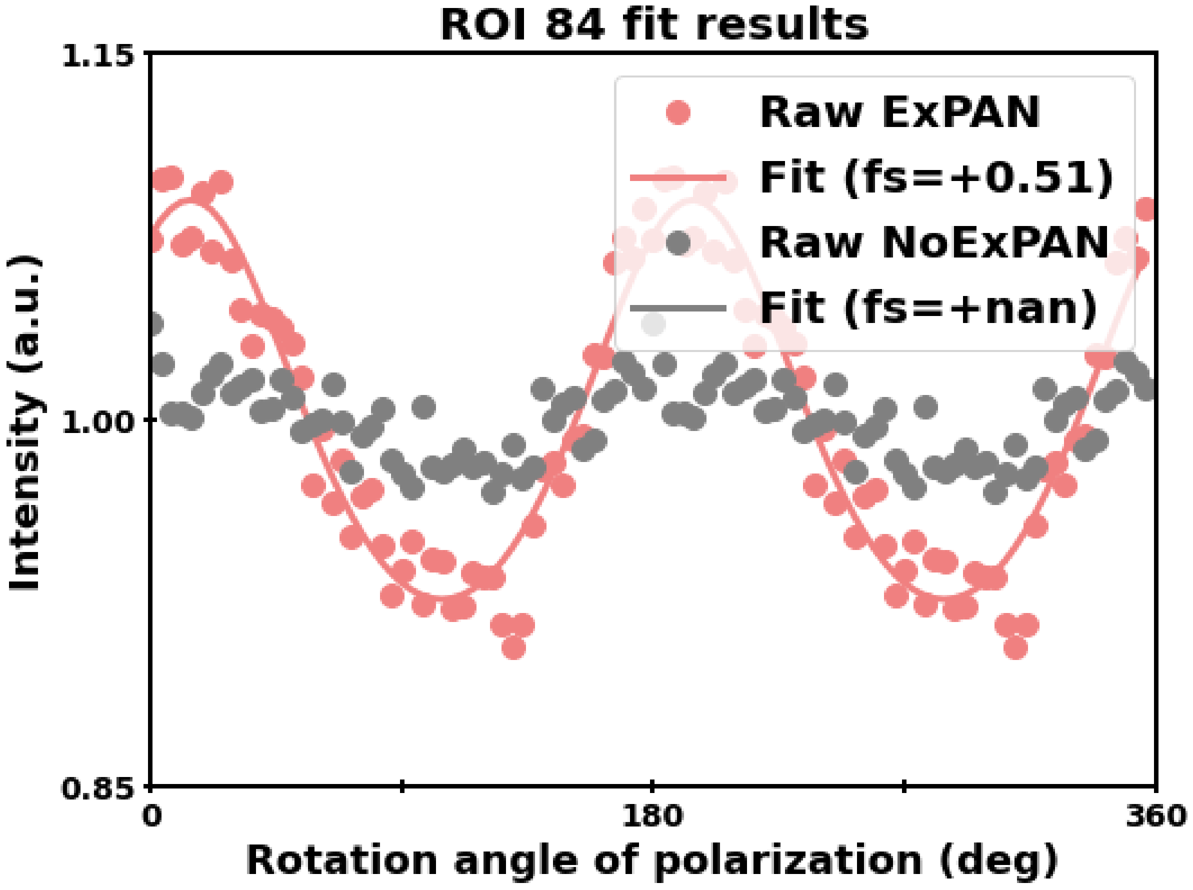
ROI 84 fit results
1.15
1.00
0.85
Intensity (a.u.)
0
180
360
Rotation angle of polarization (deg)
Raw ExPAN
Fit (fs=+0.51)
Raw NoExPAN
Fit (fs=+nan)

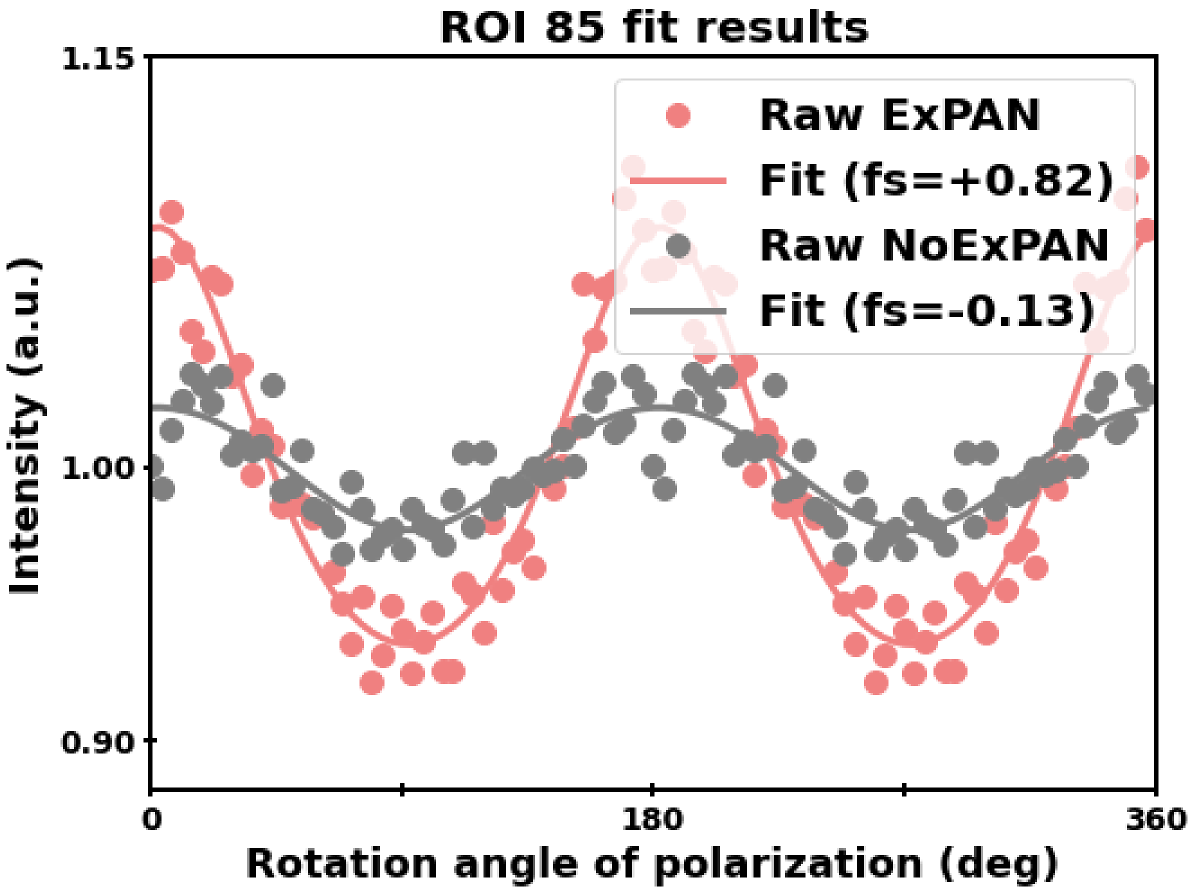
ROI 85 fit results
Raw ExPAN
Fit (fs=+0.82)
Raw NoExPAN
Fit (fs=-0.13)
1.15
1.00
0.90
Intensity (a.u.)
0
180
360
Rotation angle of polarization (deg)
